# Transcriptome-wide analysis of natural antisense transcripts shows their potential role in breast cancer

**DOI:** 10.1038/s41598-017-17811-2

**Published:** 2017-12-12

**Authors:** Stephane Wenric, Sonia ElGuendi, Jean-Hubert Caberg, Warda Bezzaou, Corinne Fasquelle, Benoit Charloteaux, Latifa Karim, Benoit Hennuy, Pierre Frères, Joëlle Collignon, Meriem Boukerroucha, Hélène Schroeder, Fabrice Olivier, Véronique Jossa, Guy Jerusalem, Claire Josse, Vincent Bours

**Affiliations:** 1 University of Liège, GIGA-Research, Laboratory of Human Genetics, Liege, Belgium; 2 University Hospital (CHU), Department of Medical Oncology, Liege, Belgium; 3 University Hospital (CHU), Center of Genetics, Liege, Belgium; 4 University of Liège, GIGA-Genomics Platform, Liege, Belgium; 5 Clinique Saint-Vincent (CHC), Department of Pathology, Liege, Belgium

## Abstract

Non-coding RNAs (ncRNA) represent 1/5 of the mammalian transcript number, and 90% of the genome length is transcribed. Many ncRNAs play a role in cancer. Among them, non-coding natural antisense transcripts (ncNAT) are RNA sequences that are complementary and overlapping to those of either protein-coding (PCT) or non-coding transcripts. Several ncNATs were described as regulating protein coding gene expression on the same loci, and they are expected to act more frequently in *cis* compared to other ncRNAs that commonly function in *trans*. In this work, 22 breast cancers expressing estrogen receptors and their paired adjacent non-malignant tissues were analyzed by strand-specific RNA sequencing. To highlight ncNATs potentially playing a role in protein coding gene regulations that occur in breast cancer, three different data analysis methods were used: differential expression analysis of ncNATs between tumor and non-malignant tissues, differential correlation analysis of paired ncNAT/PCT between tumor and non-malignant tissues, and ncNAT/PCT read count ratio variation between tumor and non-malignant tissues. Each of these methods yielded lists of ncNAT/PCT pairs that were enriched in survival-associated genes. This work highlights ncNAT lists that display potential to affect the expression of protein-coding genes involved in breast cancer pathology.

## Introduction

Over the past decade, RNA sequencing technology has enabled the determination that the non-coding part of the genome represents approximately 1/5 of the transcript number^1,2^. These non-coding RNAs (ncRNA) are less conserved between species than protein coding genes, but more conserved than introns and random intergenic regions^3,4^. It is therefore likely that these non-coding transcripts have biological roles that are progressively being deciphered, but still remain largely unknown. NATs are pairs of complementary RNA transcripts originating from the same genomic locus (*cis-*NAT) or an unlinked genomic locus (*trans-*NAT)^5^. Among *cis-*NATs, approximately 30–50% of the protein coding gene loci additionally express ncRNA in the opposite direction from the protein coding gene^4,5^. This work will focus on this particular subgroup of *cis* non-coding NATs for the reasons presented below.

NATs are less studied than other classes of RNA because their detection and quantification require the preservation of information about the transcript-originating strand along with the sequencing process. Standard RNA sequencing requires double-stranded cDNA synthesis, which erases RNA strand information, leading to expression quantification that is the sum of the expression of both transcripts of the *cis*-NAT pair. Commercial kits that can gather this information have been available for about five years and facilitate new research in the antisense transcription field, which is the subject of the present study.

NAT expression is regulated by inducible promoters and enhancers as other genes, but ncNATs accumulate preferentially in the nucleus - associated with chromatin - unlike mature coding mRNAs which are located in the cytoplasm^6–8^. NATs are also found in other cellular compartments such as mitochondria or polysomes^7–9^. ncNAT expression is described in many specific examples to affect in *cis* the expression of their sense or neighboring coding genes in biological events such as cell differentiation and carcinogenesis, with distinct molecular mechanisms being involved^8,10–12^. ncNATs can regulate gene expression in *trans* or in *cis*. Given that both the sense and antisense transcripts may be transcribed from the same genomic region, it is expected that antisense transcripts behave more frequently in *cis* than other ncRNAs that commonly function in *trans* ^8^. The fact that ncNATs may regulate their protein coding gene counterpart at the same locus is of great interest from the therapeutic point of view: ncNATs may thus provide a unique entry point for therapeutic intervention on targeted genes by the use of ASO (antisense oligonucleotides) that are drugs already FDA-approved for several diseases^10,13–16^.

To date, a few studies have been performed at the whole transcriptome scale to investigate the role of ncNATs in the context of breast cancers. These studies have demonstrated that pairs of ncNAT/PCT are globally deregulated in this pathology^16–19^. However, none of those studies compared the whole transcriptome of paired tumorous and non-malignant tissues of the same patients, with a technology that keeps the strand information of the transcripts. Yet, such an experimental design would be needed to explore if ncNAT tumor deregulations are cancer-specific, in order to better understand the role of ncNATs in the pathology. Here, we describe the results of such an experimental design, in a cohort of 22 ER+ breast cancer patients whose paired non-malignant and tumorous tissues were analyzed by stranded RNA sequencing.

This work describes co-expression patterns of ncNATs and their protein coding gene counterparts on the same locus, states the disruptions of these patterns observed in the breast cancer pathology and quantifies to what extent this phenomenon is occurring. We first defined 3 lists of ncNAT/PCT pairs that are both deregulated between adjacent non-malignant/tumorous tissues and probably related to ncNATs regulations. Next, we demonstrated that those lists are enriched with survival-associated genes. Finally, we established a list of breast cancer-related genes potentially regulated by their ncNATs that could be targeted by ASO in a therapeutic objective.

## Results

**Figure 1:**
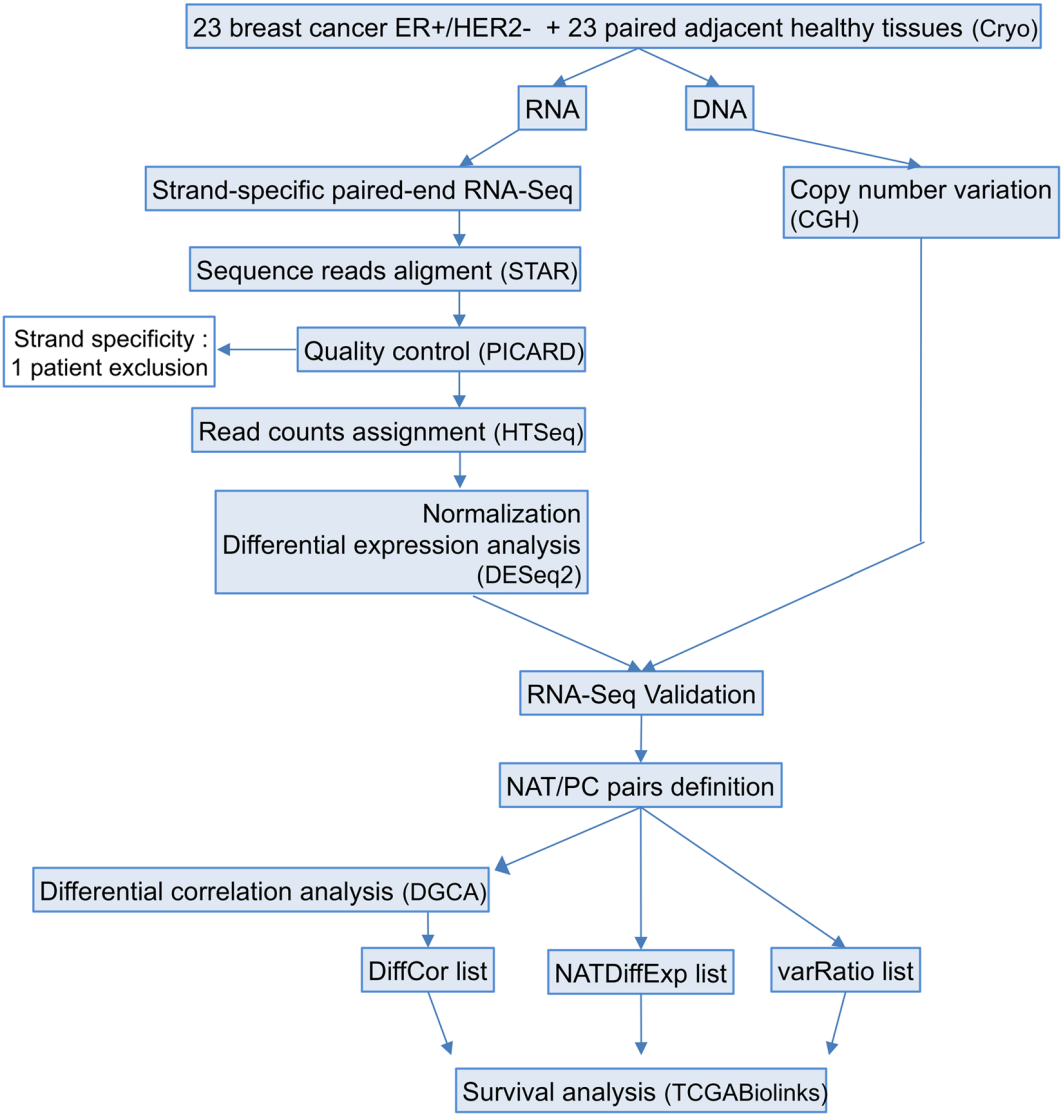
Study workflow. RNA and DNA were simultaneously extracted from 23 breast cancer ER+/HER2− tumors and their paired adjacent non-malignant tissues. Strand-specific paired-end RNA sequencing and comparative genomic hybridization (CGH) were performed. Quality control steps and RNA-Seq validation were performed and led to the elimination of one patient due to poor strand specificity in this sample. This strategy allowed the study of the differential expression of ncNATs and PCTs between tumors and non-malignant tissues and performance of differential correlation analysis of ncNAT/PCT pairs. Three lists of genes with deregulated ncNAT expression in tumors that could potentially affect the corresponding PC expression were extracted, and their coding genes were subjected to survival analysis with an external cohort (TCGA).

The role played by ncNATs on the expression regulation of their corresponding coding transcripts is still largely unknown, and this potential regulation could play a role in intermediate- and high-grade ER+ breast cancer pathology. Our study experimental design used to answer this question at the transcriptome scale is depicted in Fig. 1. Twenty-two tumorous tissues of ER+ breast cancer patients and their paired adjacent non-malignant tissues were subjected to strand-specific RNA sequencings and DNA copy number analysis by CGH array. The patient characteristics are summarized in Table 1. The cohort contains only tumors larger than 20 mm and is equally divided between luminal A and B sub-types and between highly (Ki67 > 19%) and moderately (Ki67 < 19%) proliferating tumors. Most present a Bloom grade of 2 and 3.

**Table 1:** Patient clinicopathological characteristics.

**Clinical Features**	**Criteria**	**Patients**
Age (years)	Median	62.9 years
	Range	43–83 years
Tumor size (mm)	>20	N = 22
	<20	N = 0
Ki 67 (%)	<19	N = 11
	≥19	N = 10
	Unknown	N = 1
Histology	IDC + DCIS	N = 7
	IDC	N = 15
Bloom grade	I	N = 4
	II	N = 9
	III	N = 9
T (x to 4)	1c	N = 10
	2	N = 11
	3	N = 1
N (x to 3)	0	N = 13
	1a	N = 5
	1c	N = 1
	2a	N = 2
	3a	N = 1
M (0 or 1)	0	N = 22
	1	N = 0
Molecular subtype	ER + /HER2-	N = 22
	Luminal A	N = 11
	Luminal B	N = 11
Meantime follow-up	Month	43.36

### RNA-Seq Validation

Comparison of RNA transcript expression levels between tumor and adjacent non-malignant tissues were combined with the corresponding DNA copy number variations: the overall expression levels of coding gene transcripts inside genomic amplifications or deletions that were newly acquired in the tumor were respectively increased and decreased, as expected (Supplemental File 1/Fig S1A).

Moreover, gene expression changes between non-malignant and tumor tissues obtained in our RNA-Seq dataset were compared to those obtained in an independent microarray dataset (GSE65216). Gene expression variation between 10 mammary normal tissues and 22 ER+ tumors (11 luminal A and 11 luminal B) were extracted using Geo2R^20^. The gene expression fold-changes that were differentially expressed with an adjusted p-value < 0.05 between non-malignant and tumor tissues in both our and the GSE65216 datasets were compared and presented an average Spearman correlation coefficient of 0.613 (p-value < 0.001), with 76.6% of these genes differentially modulated in the same direction (Supplemental File 1/Fig S1B). GSEA analysis using the GenePattern tool was also conducted to compare the respective gene sets enrichment in the tumor tissues of our RNAseq and the GSE65216 microarray datasets. A total of 1026 gene sets were found to be enriched in the RNASeq dataset. Among them, 870 were also enriched in the GSE65216 microarray dataset, showing an 85% homology in gene set enrichment. The 100 first enriched gene sets in tumor tissues were listed in the Supplemental file S1/Table S2. In addition, enrichment plots of several representative pathways that are differentially enriched in tumor and non-malignant adjacent tissues are presented in Supplemental File 1/Figure S3, showing comparable profiles in our RNASeq dataset and the GSE65216 microarray dataset (http://genepattern.broadinstitute.org/gp/)^21^.

At a smaller scale, RT-qPCR experiments were performed on RNA samples that were used for our RNA-Seq study to confirm variations in the expression of several transcripts between tumor and non-malignant tissues. Seventy percent of the tested transcripts showed consistent variations as measured by the two techniques. Downregulation in the tumors of the ADAMTS9 tumor suppressor and its ncNAT, ADAMTS9-AS2 are presented in Supplemental File 1/Fig S1C as representative results.

### ncNAT expression accounts for 17% of the coding counterpart in non-malignant adjacent tissues and increases to 26% in tumors

We next defined pairs of protein-coding genes and their corresponding antisenses, as detailed in the material and methods section. This list can be found in Supplemental File 2/Table 1 and contains 9632 ncNAT/PCT pairs where at least one patient has a non-null expression for PCT or ncNAT, either in the non-malignant adjacent tissue or in the tumor. As 19846 coding transcripts were expressed in mammary tissues, 49% of coding transcripts have a concomitant corresponding ncNAT expression. Globally, ncNAT read counts represent 17% of their coding counterparts in non-malignant tissues and 26% in tumors (Table 2), suggesting a global increase in the expression levels of ncNATs in mammary tumors. Moreover, the average read counts ratio between PCT/ncNAT transcript pairs expressed simultaneously by a locus is 1544 in adjacent non-malignant tissues and 1013 in tumors (Supplemental File 2/Table 1).

**Table 2:** Distribution of the relative expression intensities of ncNATs and their corresponding PCTs among the 9632 ncNAT/PCT pairs. This study was focused on ncNAT/PCT pairs where both the PCTs and the NATs were expressed in at least 7 out of the 22 patients, both in the tumor and the non-malignant tissue. This group of 4884 gene pairs contains 60% of the total reads counts, and the ncNAT/PCT ratio expression is increased in tumors.

Nber NAT/PC pairs	transcript non-null expression in more than 7/22 patients				sum of read counts			
	**PC norm tissue**	**PC tum**	**NAT norm tissue**	**NAT tum**	**PC norm tissue**	**PC tum**	**NAT norm tissue**	**NAT tum**
4884	yes	yes	yes	yes	5.56E + 07	4.73E + 07	1.73E + 07	2.26E + 07
3282	yes	yes	no	no	3.51E + 07	2.89E + 07	1.62E + 04	2.49E + 04
944	yes	yes	no	yes	1.08E + 07	1.02E + 07	1.03E + 04	2.12E + 04
149	yes	yes	yes	no	2.06E + 06	1.17E + 06	2.61E + 03	2.06E + 03
149	no	no	yes	yes	4.81E + 02	9.21E + 02	7.11E + 04	6.73E + 04
89	no	no	no	no	2.51E + 02	3.85E + 02	5.65E + 02	8.20E + 02
50	no	yes	yes	yes	9.32E + 02	1.77E + 03	2.27E + 04	4.29E + 04
29	no	yes	no	no	5.16E + 02	9.14E + 02	1.35E + 02	2.42E + 02
28	no	no	no	yes	9.85E + 01	1.64E + 02	3.23E + 02	5.69E + 02
11	yes	no	yes	yes	2.42E + 02	2.38E + 02	2.02E + 03	2.32E + 03
7	no	yes	no	yes	9.24E + 01	1.93E + 02	9.25E + 01	1.88E + 02
5	yes	no	no	no	1.50E + 02	1.43E + 02	1.61E + 01	3.80E + 01
3	no	no	yes	no	3.26E + 01	2.27E + 01	4.59E + 01	8.58E + 01
1	yes	no	yes	no	5.67E + 01	1.53E + 01	1.00E + 01	1.48E + 01
1	no	yes	yes	no	4.84E + 00	8.32E + 00	1.53E + 01	2.86E + 01
0	yes	no	no	yes	0.00E + 00	0.00E + 00	0.00E + 00	0.00E + 00
Total 9632	NAT/PCT pairs where at least one patient has a non-null expression in one of those four transcript types				1.04E + 08	8.75E + 07	1.74E + 07	2.28E + 07
					2.31E + 08			

The 9632 PCT/ncNAT transcript pairs contain 6494 unique protein-coding genes and 8861 unique ncNATs genes. Forty percent of the PCT/ncNAT transcript pairs (3888) display a unique transcript on the opposite strand, while the other pairs overlap with two to nine transcripts. The detailed overlap between PCT and ncNAT transcripts is described in the Supplemental File 1/Table S4.

**Figure 2:**
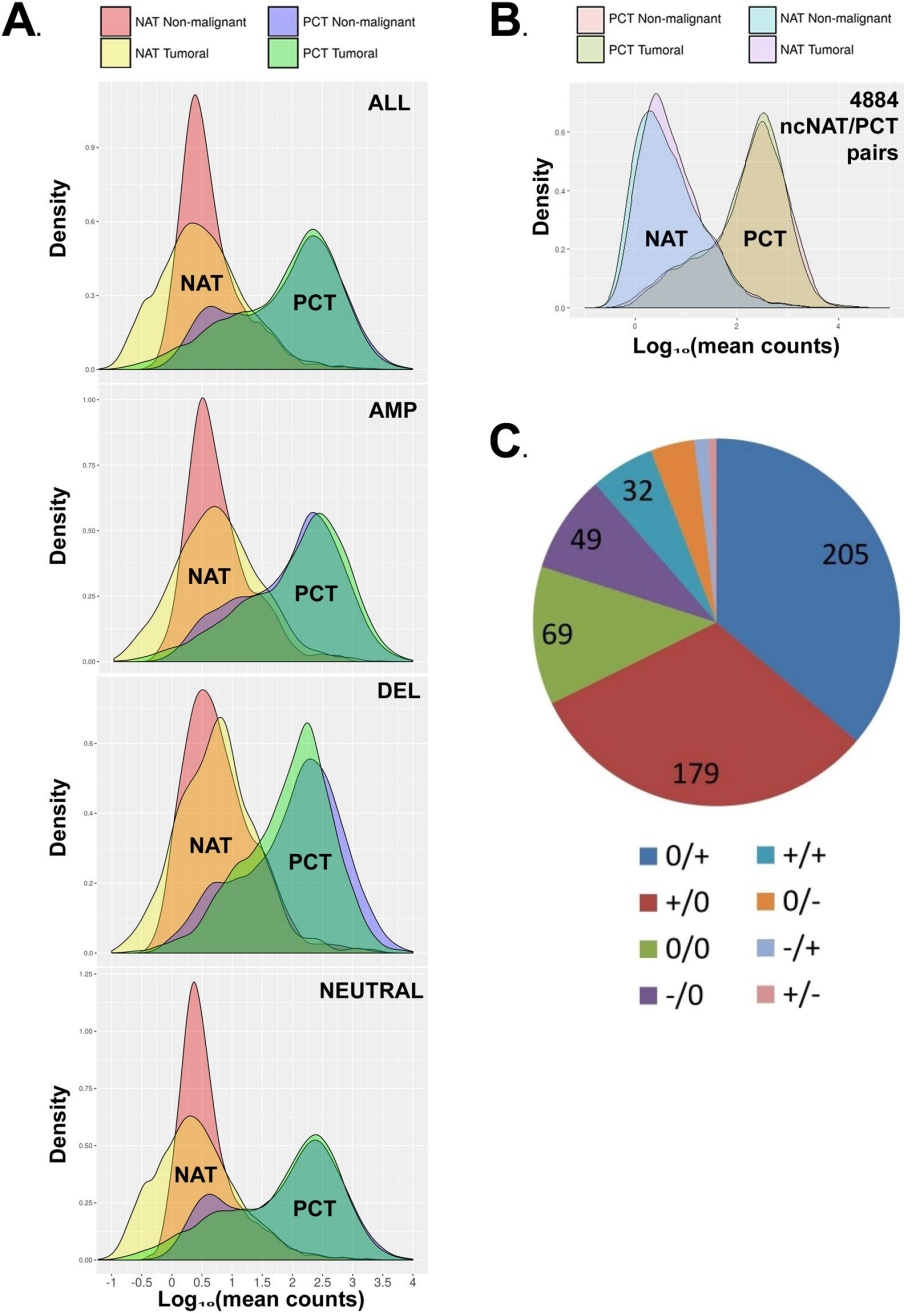
(**A**). ncNAT and PCT transcript distributions in adjacent non-malignant healthy tissue and tumors. Density plot of log_10_ (mean counts) of the four transcript types (ncNAT tumor; ncNAT non-malignant, PCT tumor, PCT non-malignant). The upper panel contains the complete list of 9632 ncNAT/PCT pairs; the second panel contains ncNAT/PCT pairs in amplified genomic regions; the third panel contains ncNAT/PCT pairs in deleted genomic regions; and the last panel contains ncNAT/PCT pairs in copy-number-neutral genomic regions. The ncNAT distributions are clearly different in non-malignant and tumor tissues, with a higher number of ncNATs displaying reduced expression in the tumors than the number of ncNATs that are increased in the tumors. (**B**) Presents the same information as panel A, but in the highly expressed ncNAT subgroup of the 4882 ncNAT/PCT pairs. (**C**) Schematic representation of proportion of different classes of differential correlations between tumors and non-malignant tissues. Primarily positive correlations of expression between ncNAT and their corresponding PCT are created or lost in tumorous tissues. The numbers indicated in the graph are the number of ncNAT/PCT pairs in this category; +/+= significant positive correlation between ncNAT and PCT in adjacent non-malignant tissue and conserved in the tumor; +/−= significant positive correlation between ncNAT and PCT in the adjacent non-malignant tissue that becomes negative in the tumor; +/0= significant positive correlation between ncNAT and PCT in the adjacent non-malignant tissue that is lost in the tumor; −/−= significant negative correlation between ncNAT and PCT in adjacent non-malignant tissue that is conserved in the tumor; −/+= significant negative correlation between ncNAT and PCT exists in adjacent non-malignant tissue that becomes positive in the tumor; −/0= significant negative correlation between ncNAT and PCT in the adjacent non-malignant tissue that is lost in the tumor; 0/0 = no significant correlation between ncNAT and PCT exists in the adjacent non-malignant tissue or the tumor.

The reads count distributions of the four transcript types among the 9632 ncNAT/PCT pairs are represented in Fig. 2A. The ncNAT distributions are clearly different in non-malignant and tumor tissues (Mann-Whitney Wilcoxon pval = 2.2E-16 in ALL genomic variation status; pval = 0.145 in AMP; pval = 0.0022 in DEL; pval = 2.2E-16 in NEUTRAL), with a higher number of ncNATs displaying reduced expression in the tumors (yellow surface at the left of red peak) compared to the number of ncNATs that are increased in the tumors (yellow surface at the right of the red peak). The genomic amplifications and deletions affect the ncNAT transcripts distributions less than the cancerous or non-malignant status of the tissue (comparing the AMP or DEL distribution to the NEUTRAL distribution). Indeed, genomic alterations are relatively occasional in ER+ breast cancers.

Based on the levels of expression in the different tissue types, we chose to focus on ncNAT/PCT pairs where both the PCT and NAT were expressed in at least seven of the 22 patients, both in the tumor and the non-malignant tissue. This represents more than 60% of the total read counts of ncNAT/PCT pairs (Table 2). This choice was made because the next gene analysis is mainly based on transcript expression correlations and expression ratios. A total of 4748 ncNAT/PCT pairs present low expression in at least one of the four categories compared (PCT tumor, PCT non-malignant, ncNAT tumor, ncNAT non-malignant), leading to aberrant Spearman correlation values or an invalid expression ratio. To avoid this pitfall, we only kept ncNAT/PCT pairs with at least seven non-null values in the four categories, corresponding to one third of the patient cohort. This choice ensures that the statistical tests are accurate and restricts the finding to recurrent observations, which are more interesting from medical and therapeutic standpoints. In this group of 4884 transcript pairs, the number of tumor ncNAT read counts increased compared to the adjacent non-malignant tissues (Table 2). Moreover, the ncNAT/PCT ratio of 31% in adjacent non-malignant tissues is increased to 47.8% in tumors. The broken balance of expression between ncNAT and PCT observed in the tumor could increase the probability of the occurrence of deregulated mechanisms, and hence this gene sub-group could contain PC transcripts that display a stronger potential for regulation by their ncNAT counterparts.

The discordant ncNAT/PCT pairs can occur either due to variations in ncNAT transcript expression between tumors and non-malignant tissues, or PCT variations, or variations in both. To determine which case is more frequently observed in the 4884 ncNAT/PCT pairs, the mean counts of the four categories of transcripts (PCT tumor, PCT non-malignant, ncNAT tumor, ncNAT non-malignant) for the ncNAT/PCT pairs in the 22-patient cohort were plotted (Fig. 2B). The results show that ncNAT was expressed approximately 1000-fold less than PCT. The distribution of PCT read counts is quite comparable in the tumor and adjacent non-malignant tissue. However, ncNAT read counts appeared to be slightly increased in the tumors compared to the adjacent non-malignant tissues (shift of the curve to the right). This suggests that the discordant ncNAT/PCT pairs mainly occur via modifications of the ncNAT expression in the tumors.

### Positive correlations of expression between ncNAT and corresponding PCT are created in tumorous tissues

To highlight newly appearing or disappearing correlations between ncNATs and their corresponding PCTs in tumors, differential correlation analysis between all pairs of PCTs and ncNATs was performed using DGCA software (v. 1.0.1)^22^. Complete results can be found in Supplemental File 2 / Table 1, showing a global positive correlation of expression between ncNATs and their corresponding PCTs: the mean Spearman correlation coefficients were 0.431 and 0.533, respectively, in non-malignant tissues and tumors when a significant correlation was observed (p-value < 0.05), namely, in 20% of the 4884 ncNAT/PCT pairs. The number of significantly correlated ncNAT/PCT pairs does not differ in non-malignant and tumorous tissues. A positive mean z-score (0.460) is observed in the case of a significant differential correlation of ncNAT/PCT between tumor and non-malignant tissues (p-value < 0.05), meaning that globally, in the 11% of ncNAT/PCT pair correlations that are deregulated in tumors compared to non-malignant tissues (567/4884 pairs), the correlations become more positive. The proportion of different classes of differential correlations between tumors and non-malignant tissues is depicted in Fig. 2C, highlighting the fact that mainly positive correlations of expression between ncNATs and their corresponding PCTs are created or lost in tumorous tissues. Very few inversions of correlation were observed.

Some examples of ncNAT/PCT pairs demonstrating deregulated correlation of expression in the tumor tissue are presented in Table 3, which shows that genes well-known in the breast cancer field display deregulated correlation of expression with their antisense transcripts.

**Table 3:** Examples of spearman correlations between ncNAT and cancer-associated coding genes that are altered in tumors when compared to adjacent non-malignant tissues.

Coding Gene Name	ncNAT_ID_Ensembl	ncNAT-Name	ncNAT/PCT spearman correlation adj non-malignant tissue	ncNAT/PCT cor. p-val adj non-malignant tissue	ncNAT/PCT spearman correlation tumor tissue	ncNAT/PCT cor. p-val tumor tissue	zScore_DiffCor_NAT/PCT	pVal_DiffCor_NAT/PCT	Classes_DiffCor
ACSL6	ENSG00000234758	AC034228.4	−0.353	1.07E-01	0.441	4.01E-02	2.59	9.48E-03	0/+
AFF1	ENSG00000235043	TECRP1	0.762	3.74E-05	0.247	2.69E-01	−2.31	2.08E-02	+/0
BRCA1	ENSG00000240828	RPL21P4	0.625	1.87E-03	−0.041	8.57E-01	−2.39	1.70E-02	+ /0
CAMTA1	ENSG00000225126	RP4-549F15.1	−0.093	6.82E-01	0.698	3.06E-04	2.95	3.22E-03	0/+
CAMTA1	ENSG00000269978	RP11-338N10.3	0.817	3.41E-06	0.139	5.36E-01	−3.11	1.87E-03	+/0
CDKN2B	ENSG00000240498	CDKN2B-AS1	−0.541	9.31E-03	0.269	2.27E-01	2.72	6.61E-03	−/0
CEBPA	ENSG00000267727	CTD-2540B15.7	0.788	1.31E-05	−0.013	9.55E-01	−3.33	8.74E-04	+/0
CTCF	ENSG00000237718	AC009095.4	−0.249	2.63E-01	0.577	4.96E-03	2.81	4.93E-03	0/+
GNA11	ENSG00000267139	AC005262.3	−0.135	5.49E-01	0.483	2.28E-02	2.04	4.11E-02	0/+
GNAS	ENSG00000235590	GNAS-AS1	−0.599	3.22E-03	0.261	2.41E-01	2.96	3.12E-03	−/0
HLF	ENSG00000263096	RP11-515O17.2	−0.494	1.95E-02	0.142	5.29E-01	2.11	3.50E-02	−/0
HNF1A	ENSG00000241388	HNF1A-AS1	0.023	9.18E-01	0.668	6.87E-04	2.41	1.58E-02	0/+
HOXC11	ENSG00000228630	HOTAIR	0.078	7.29E-01	0.625	1.87E-03	2.02	4.36E-02	0/+
MAP2K1	ENSG00000269999	CTD-3185P2.2	−0.523	1.26E-02	0.099	6.62E-01	2.09	3.64E-02	−/0
MSH2	ENSG00000236558	AC138655.6	0.762	3.74E-05	0.156	4.88E-01	−2.60	9.26E-03	+/0
MSH6	ENSG00000224058	AC006509.7	0.567	5.89E-03	−0.100	6.58E-01	−2.29	2.18E-02	+/0
MYH11	ENSG00000263065	AF001548.6	0.662	7.94E-04	0.030	8.95E-01	−2.36	1.82E-02	+/0
PPP6C	ENSG00000232630	PRPS1P2	−0.187	4.05E-01	0.453	3.41E-02	2.09	3.66E-02	0/+
RAP1GDS1	ENSG00000214559	RP11-323J4.1	0.159	4.78E-01	0.911	3.99E-09	4.23	2.38E-05	0/+
UBR5	ENSG00000246263	KB-431C1.4	0.164	4.65E-01	0.813	4.16E-06	2.99	2.76E-03	0/+
UBR5	ENSG00000272037	KB-431C1.5	0.235	2.91E-01	0.735	9.92E-05	2.15	3.13E-02	0/+
WT1	ENSG00000183242	WT1-AS	0.392	7.09E-02	0.885	4.46E-08	3.03	2.42E-03	0/+
ZFHX3	ENSG00000259901	RP5-991G20.4	−0.322	1.44E-01	0.503	1.71E-02	2.73	6.26E-03	0/+

### Protein-coding genes exhibiting corresponding deregulated ncNAT expression in tumors are preferentially related to survival of breast cancer patients

Three different analytical methods were used to select ncNAT/PCT pairs that were potentially related to breast cancer pathology out of the 4884 pairs.

First, the previously described DiffCor is based on the differential correlation of ncNAT/PCT read counts between non-malignant and tumor tissues. A list of ncNAT/PCT pairs whose correlation was significantly different between normal and tumor tissues (p-value < 0.05) and whose correlation class differed between tumor and normal tissues (i.e., 0/0, +/+, −/− classes are removed) was selected and contained 441 ncNAT/PCT pairs.

The second method is based on the differential expression of ncNATs between tumors and non-malignant tissues. A list of 738 ncNAT/PCT pairs was determined, for which ncNATs were significantly differentially expressed (adjusted p-value < 0.05) between normal and tumor tissues.

The third method is based on the variation in the ncNAT/PCT ratio between non-malignant and tumor tissues, and allows the definition of a third list, called VarRatio, that contains ncNAT/PCT pairs with extreme values in the distribution of the VarRatio (Supplemental File 1/Figure S5). This VarRatio list can be subdivided into leftmost and rightmost parts. The leftmost 610 ncNAT/PCT pairs have a PCT/ncNAT ratio that decreases in the tumor, either because of a down-regulation in PCT expression, or an up-regulation in ncNAT expression, or both; the reverse is observed for the 540 ncNAT/PCT pairs on the rightmost end of the distribution.

The three lists of genes can be found in the Supplemental File 1/Tables S6 to S9, and as expected, many ncNAT/PCT pairs appear in several of these lists, which contain a total of 1784 unique ncNAT/PCT pairs that are deregulated in breast cancers.

To ascertain if the protein coding genes in the DiffCor, ncNATDiffExp, and VarRatio lists are implicated in breast cancer pathology, their association with survival was computed based on RNA-Seq samples from the TCGA dataset. Each of these three lists present a proportion of genes associated with survival in the TCGA dataset greater than the proportion obtained in a list of randomly chosen protein coding genes (Table 4). This means that PCT exhibiting corresponding deregulated ncNAT expression in tumors are enriched in genes related to the survival of breast cancer patients. A Pearson’s chi-squared test yielded statistically significant p-values for each of the three lists compared to both control lists.

**Table 4:** PC genes exhibiting deregulated corresponding ncNAT expression in tumor are preferentially related to survival of breast cancer patients.

**PC Gene list**	**DiffCor**	**ncNATDiffExp**	**VarRatio Left**	**VarRatio Right**	**Random protein-coding genes with antisense overlap**	**Random protein-coding genes without antisense overlap**
Nb genes in list	441	738	610	540	582	582
Nb genes also present in TCGA dataset	440	729	604	533	582	582
Nb genes w/ log-rank p-val <= 0.05	71	118	96	84	57.38 ± 7.32	41.48 ± 6.61
Genes % w/ log-rank p-val <= 0.05	16.1%	16.2%	15.9%	15.8%	13.83 ± 1.78%	12.27 ± 1.91%
Average log-rank p-val	0.392	0.387	0.388	0.391	0.404 ± 0.0315	0.483 ± 0.357

Protein coding genes of the 3 gene lists DiffCor, ncNATDiffExp and VarRatio (left and right) were tested for association with survival by means of a TCGA RNA-Seq dataset of breast cancers. The percentage of genes associated with survival in each list has been compared with 2 control distributions containing randomly selected protein coding genes.

Analyses were conducted to explore the relationship of the protein coding genes of DiffCor, ncNATDiffExp, and VarRatio lists to known prognostic factors, but no significant results were found (Supplemental File 1/Table S10). In the same way, enrichment analyses in pathways genes were conducted, without any noticeable results. The proportion of various different ncNAT biotypes in the (i) DiffCor classes of ncNAT/PCT pairs, and (ii) the DiffCor, ncNATDiffExp, and VarRatio lists were calculated and did not show any statistically significant differences. The mean p-value of ncNAT/PCT expression correlation in tumors and adjacent non-malignant tissues were also similar in the different ncNAT biotypes.

### 72 cancer genes present a deregulated profile of ncNAT expression in breast cancer samples

When the Cancer Gene Census list of genes from the COSMIC database (http://cancer.sanger.ac.uk/census) was compared to our three lists of genes that are potentially regulated by their ncNATs and implicated in breast cancer pathology, 72 genes were shared (Supplemental File 1/Table S11). This list of 72 genes contains cancer genes that could be targeted by ASO and designed to interact with the corresponding ncNATs of those genes to specifically regulate their expression.

## Discussion

Breast cancer constitutes a public health problem: approximately 1 in 8 women will suffer from breast cancer during her lifetime in industrialized countries. The most frequent subtype is estrogen-receptor-expressing breast cancer (ER+/HER2−), which accounts for 75% of occurrences^23^. In primary disease, most patients are treated with surgery with or without radiotherapy and endocrine therapy. However, a large number of these patients will suffer from a relapse and develop metastases^24^—a major life-threatening event that is strongly associated with poor outcomes—and will require chemotherapy in the case of symptomatic visceral disease^25^. New therapies are thus needed, as are biomarkers that would give a better prediction of the relapse risk^24,26^. Our study explores the still new field of antisense transcription to define potential target gene lists and will lead to further work to define predictive markers and/or tailor targeted treatment using antisense oligonucleotides (ASO)^15^.

This is the first time that a whole transcriptome strand-specific RNA-Seq study focusing on antisense transcription has been performed in paired tumor and adjacent non-malignant mammary tissues. This experimental design allows the detection of deregulation of ncNAT expression that occurs in cancer tissues and statistically connects them to changes in the corresponding coding transcript expression. A global increase in transcription due to sustained cell proliferation is described in cancers, and the observed increased ncNAT expression could be attributed to this phenomenon or to impaired antisense transcript degradation. However, as the respective proportional rise of ncNAT and PCT in the transcript pairs encoded on the same loci differs in tumors and adjacent non-malignant tissues, deregulated mechanisms of gene expression control could arise. In particular, we revealed that many positive correlations between ncNATs and their PCT counterparts were appearing or fading in the tumor, suggesting newly acquired or lost regulations of protein-coding transcripts in the cancerous tissues. Moreover, the association of these ncNATs with survival was evaluated through the use of their protein-coding counterparts as a proxy in a large independent cohort. The detected associations suggest that the dysregulation observed within the landscape of ncNATs is not merely a random byproduct of the tumoral process. However, this work is based on correlation analysis, and does not demonstrate any causative aspect. As a result, further functional molecular studies will be needed to confirm the existence of such regulation of PCTs by their ncNATs in the list of cancer gene pairs highlighted in this work. Enrichment analyses were conducted to explore the lists of ncNAT/PCT pair genes defined in this study, but no noticeable result was obtained. In the same way, no enrichment in transcription-factor binding sites was observed, in contrast with a previous report stating that the GABPA transcription factor is associated with antisense promoters in breast cancer^16^.

Several studies have already explored the role of antisense transcription in breast cancer^16–19,27^. Grinchuk *et al*. analyzed ncNAT/PCT pairs that are deregulated in breast cancer to define the pathways in which they are particularly involved; these researchers defined ncNAT/PCT-based prognosis signatures that were validated in additional cohorts. Affymetrix microarray datasets were used to identify the natural antisense gene pairs, but normal breast epithelium and mammary tumor tissues analyzed were not matched^16^. Moreover, Balbin *et al*. performed a large scale, genome wide, stranded RNA-Seq study of 376 cancers samples with 60 primary breast cancers among them^17^. However, as in Grinchuck’s study, tumorous tissues were not matched with non-malignant tissues and thus these studies did not explore, patient by patient, whether the ncNAT/PC expression correlations were already present in the normal tissue, or whether they were newly acquired in the tumor. Despite the relatively small number of patients and samples, this particularity in our experimental design did allow us to highlight the fact that ncNAT expression is increased in tumorous tissues compared to their coding counterparts. Indeed, the proportion of ncNAT read counts in ncNAT/PCT pairs in ER-positive intermediate- and high-grade estrogen-positive breast cancers is globally increased in tumors compared to adjacent non-malignant tissues.

As Balbin *et al*. have stated before, at any locus where PCT and ncNAT are simultaneously transcribed, PCT is expressed approximately 1000-fold more than ncNAT, but we have additionally observed that this difference in expression is lower in tumors than in non-malignant tissues. We also found that, globally, 10% of transcripts come from the antisense strand in non-malignant tissues and that this proportion is increased to 13% in tumors (8% were described by Balbin *et al*.). However, some patients presented a much higher increase in the ncNAT/PCT proportion in the tumor than others. This heterogeneity in ncNAT expression deregulation across patients could be used to stratify patients into subgroups with different prognoses. One limitation of our study is the small size and the short follow-up of our cohort, which did not allow such analysis and weakened the statistical power needed to show a potential relationship between ncNAT expression and known prognostic factors, which is commonly recommended for biomarker selection. Another biological limitation is that, as NAT expression is low, false positive and negative results in the correlation analysis are frequent when applied to the complete 9632 NAT/PCT pairs list. This issue was circumvented by limiting the analyses to the highly expressed NAT subgroup of 4884 NAT/PCT pairs.

Our results confirmed the observation made by Grinchuk *et al*., who showed that the NAT content in breast tumors globally increases and that the expression correlations between ncNAT and PCT were different in tumors compared to unrelated non-malignant tissues^16,19^. We refined this observation using paired tissues from the same patient and showed that globally these correlations become more significant and more positive in the tumors. Moreover, we highlighted the gene pairs where potential new PCT/ncNAT expression regulation occurs in cancerous tissues. After performing a survival analysis with gene expression data from an external cohort (TCGA), it appears that these ncNAT/PCT gene pairs were also enriched in survival-associated genes, suggesting that opposite-strand transcription regulation might play a role in breast cancer disease.

Therefore, our report indicates that ncNAT expression is often increased in cancer samples compared to matched non-malignant adjacent tissues. The relevance of this observation for coding gene expression, cancer biology, prognosis and treatment will need to be determined in large, specific cohorts of paired samples with long-term follow-up of the patients.

## Material and Methods

### Ethical Statement

Tissues were obtained from the Liege University Biobank (N = 12) and the St Vincent Clinic of Rocourt (N = 11), Belgium. This study was approved by the local institutional ethical board (“Comité d’éthique hospital-facultaire universitaire de Liège (707)) under the file number 2010/229. All aspects of the study comply with the Declaration of Helsinki. Patients from Liege University Hospital were recruited on the basis of an opt-out methodology. Patients from the St Vincent Clinic of Rocourt were informed of the research work and provided with written informed consent.

### Patients and samples

This retrospective study was performed on 23 cryopreserved cancerous and adjacent non-malignant tissues from 23 women suffering from estrogen receptor-expressing breast cancer. Samples were collected from 2010 to 2014. One patient was excluded due to the poor strand-specificity of the RNA-Seq. The clinical and pathological parameters of the patients included in the final analysis were recorded and are summarized in Table 1.

A summary of the experimental design is depicted in Fig. 1.

### DNA/RNA/miRNA extraction

DNA, RNA and miRNA were simultaneously extracted using an All Prep DNA/RNA/miRNA Universal kit (Qiagen, Belgium) according to the manufacturer’s protocol. The RNA quality was assessed using a BioAnalyzer (Agilent, Belgium).

### TruSeq® Stranded Total RNA by Illumina® and next generation sequencing

RNA sequencing libraries for 23 breast tumors and paired adjacent tissues were constructed from 500 ng of total RNA using the TruSeq® Stranded Total RNA kit and the Ribo-Zero rRNA Removal kit. A step of chemical fragmentation generated RNA fragments of 180 pb. This step was adapted according to RNA quality as described in the manufacturer’s protocol. The syntheses of the first and second strands of cDNA were performed with random hexamer primers. Twelve cycles of PCR were performed to amplify the libraries. The quality and size of the cDNA libraries were assessed using Bioanalyzer Agilent Chip DNA 1000. Only libraries from 290 bp to 300 bp were used, and 14 pmol final cDNA libraries were loaded on an Illumina HiSeq2000 apparatus for cluster generation and paired-end sequencing of 2 × 100 bp, with a mean of 8.26E + 09 bases sequenced for each sample (4 samples/flow cell). Kits and apparatus were from Illumina, The Netherlands.

### CGH array

Array comparative genomic hybridization was performed in non-malignant and tumorous tissues from the 23 patients using the Agilent 60 K microarray platform (G4827A-031746; Agilent Technologies, Santa Clara, CA, USA) according to the manufacturer’s instructions. The arrays were scanned with a SureScan High Resolution Microarray Scanner (Agilent Technologies, Santa Clara, CA, USA). Data and images were imported using Feature Extraction V.9.5.3.1 Software, and results were analyzed with CytoGenomics software v2.5 (Agilent Technologies, Santa Clara, CA, USA). The Aberration Detection Methods 2 algorithm (ADM-2) was used with a cut-off of 6.0, followed by a filter to select regions with three or more adjacent probes and a minimum average log2 ratio ± 0.25, which was used to detect copy number changes. The quality of each experiment was assessed by measuring the derivative log ratio spread using CytoGenomics software v2.0. Genomic positions were based on the UCSC February 2009 human reference sequence (hg19) (NCBI build 37 reference sequence assembly). Copy number changes were filtered using the BENCHlab CNV software (Cartagenia, Leuven, Belgium).

### Gene expression quantification by RNA-Sequencing

Quality control for the sequenced reads has been performed with FastQC software (v. 0.11.2; https://www.bioinformatics.babraham.ac.uk/projects/fastqc/). Sequencing reads were mapped to the Human Genome hg19 GRCh37-75 (Ensembl) using Star 2.4.1c software^28^. Mapping quality was assessed with the Picard RnaSeqMetrics tool in the Picard software suite (v. 1.127; http://broadinstitute.github.io/picard/) using default parameters. The results are available in Supplemental File 1/Table S12. Read count assignment was performed with the htseq-count tool of the HTSeq software suite (v. 0.6.1)^29^. Data quality assessment was performed by computing the strand specificity (ratio of sequencing reads mapping to incorrect strands) of all samples with htseq-count, leading to the removal of one patient who showed aberrant strand specificity. DESeq. 2 software (v. 1.10.1) was used to normalize read counts, estimate dispersion, perform variance stabilizing transformation, and perform independent filtering using the mean of normalized counts as a filter statistic, thereby adjusting the filtering threshold at 33%, following the standard workflow^30^. Variance-stabilization performance was assessed by producing MA-plots of log2-fold-change versus mean expression using DESeq. 2. Outliers were searched for by computing Cook’s distances for every gene and every sample with DESeq. 2 (Supplemental File 1/Figures S13–S17). A principal component plot was performed to assess the appropriate separation between the two sample classes (Supplemental File 1/Figure S18). All aforementioned quality and performance measures yielded acceptable results for all remaining samples.

### Data availability

The raw and processed RNASeq data were submitted to GEO DataSet under the series record GSE103001.

### Quantitative RT-qPCR

Reverse transcription was performed using the Reverse Aid H Minus kit (LifeTechnologies, Belgium) from 100 ng of total RNA using random hexamer primers in the case of coding genes and using target-specific primers coupled to an unrelated synthetic DNA oligonucleotide in the case of ncNAT.

Quantitative PCR were performed using specific 6-FAM/ZEN/IBFQ probes (IDT, Belgium) with Kapa Probe Fast qPCR Master Mix (Sopachem, Belgium) on a LightCycler 480 apparatus (Roche). In the case of coding gene amplification, the primers were designed according to a standard procedure. In the case of ncNAT gene amplification, a primer specific to the target ncNAT and a primer specific to the synthetic oligonucleotide added during reverse transcription were used to allow strand-specific amplification.

The relative expression was calculated using the standard curves methods, using beta−2-microglobuline as endogenous standard.

Primers and probe sequences can be found in the Supplemental File 1/ Additional Materials and Methods.

### External dataset used for RNASeq gene expression comparison

Gene expression variations were retrieved from the de GEO Dataset GSE65216 (https://www.ncbi.nlm.nih.gov/geo/query/acc.cgi?acc=GSE65216), which is an expression study using the micro-array (Affymetrix) of the Maire’s breast cancer cohort^31^.

### Definition of protein-coding/antisense pairs

The list of pairs of protein-coding genes and their corresponding antisense transcripts was generated based on human genome assembly and gene annotation GRCh37 (release 75) from Ensembl^32^. To be included in the list, pairs of genes had to fulfill the three following conditions: overlapping coordinates; opposite strands; one of the two genes must have a protein_coding biotype, while the other can have any of the following biotypes: *3prime_overlapping_ncrna, antisense, IG_C_pseudogene, IG_V_pseudogene, lincRNA, misc_RNA, polymorphic_pseudogene, processed_transcript, pseudogene, sense_intronic, sense_overlapping, snoRNA, snRNA*. The reasoning behind including all non-protein_coding biotypes as putative antisenses is that the Ensembl annotation of antisense is limited to already validated antisenses and thus might miss previously unknown antisenses.

### ncNAT/PCT Gene list selection methods

1. DiffCor list: Differential correlation analysis between pairs of protein-coding and antisense transcripts was performed using DGCA software (v. 1.0.1)^22^. Pairs of protein coding/antisense genes were selected for which the correlation significantly differed between normal and tumor samples (adjusted p-value < 0.05) and for which correlation class differed between tumor and normal samples (i.e., we removed the 0/0, +/+, −/− classes).
2. ncNATDiffExp list: Differential expression analysis between all tumor and non-malignant samples was performed using DESeq. 2 software (v. 1.10.1) for all genes, following the standard multi-factor workflow for paired samples. Pairs of protein coding/antisense genes were selected for which the antisense was significantly differentially expressed (adjusted p-value < 0.05) between normal and tumor samples.
3. varRatio list: Read count ratio variation analysis was performed as follows: define the variation of read counts ratio (varRatio) for each pair of ncNAT/PCT genes as

Equation 1

$${var}\,{Ratio}=\frac{{tumoral}\,{read}\,{countsratio}}{{normal}\,{read}\,{countsratio}}$$

where

Equation 2

$$tumoral\,read\,counts\,ratio=\frac{\sum tumor\,read\,count{s}_{antisense}}{\sum tumor\,read\,count{s}_{proteincoding}}$$

and

Equation 3

$$normal\,read\,counts\,ratio=\frac{\sum normal\,read\,count{s}_{antisense}}{\sum normal\,read\,count{s}_{proteincoding}}$$

Pairs of ncNAT/PCT genes corresponding to extreme values of the varRatio distribution were selected by applying a threshold (mean ± standard deviation) to the log-transformed distribution of the varRatios (Supplemental File 1/Figures S5).

For all three gene list selection methods, pairs of genes in which either the protein-coding or the antisense was expressed in less than seven tumor samples or seven adjacent non-malignant samples were discarded.

### Survival analysis

All protein-coding genes from the three gene lists have been tested for association with survival using an external dataset of 1066 RNA-Seq samples from the tumors of female breast cancer patients (Package R TCGA Biolinks^33^). Association with survival was recorded when the p-value of a log-rank test was less than 0.05. The ratio of genes associated with survival in each list has been compared using the same ratio computed with two control distributions (95% CI of 200 random pair distributions) of protein-coding genes: the first control contained protein-coding genes with no overlap with the antisense, while the second control contained protein-coding genes overlapping the antisense (i.e., protein-coding genes from previously defined 9632 pairs of protein-coding genes and antisense) but were not selected by the three analysis methods used.

## Electronic supplementary material

Supplemental File 1

Supplemental File 2
