# Supplementary material for: Transcriptome-wide analysis of natural antisense transcripts shows their potential role in breast cancer
Source: Sci Rep. 2017 Dec 12;7:17452. doi: 10.1038/s41598-017-17811-2 (PMC5727077; doi:10.1038/s41598-017-17811-2)
Supplement: Supplementary file 1 — Supplemental File 1 [file 41598_2017_17811_MOESM1_ESM.pdf]

Title

Transcriptome wide analysis of natural antisense transcripts shows their potential role in breast cancer.

Authors

Stephane Wenric, Sonia ElGuendi, Jean-Hubert Caberg, Warda Bezzaou, Corinne Fasquelle, Benoit Charloteaux, Latifa Karim, Benoit Hennuy, Pierre Frères, Joëlle Collignon, Meriem Boukerroucha, Hélène Schroeder, Fabrice Olivier, Véronique Jossa, Guy Jerusalem, Claire Josse and Vincent Bours.

Supplemental file 1

Table of content

Table of content	
Supplemental File 1/ FigS1	RNASeq validation
Supplemental File 1/ Table S2	GSEA : pathways enrichment table
Supplemental File 1/ FigS3	GSEA : enrichment plot
Supplemental File 1/ Table S4	Description of ncNAT/PCT pairs overlap
Supplemental File 1/ FigS5	Varatio list description
Supplemental File 1/ TableS6	DiffCor gene list
Supplemental File 1/ TableS7	VarRatio Right gene list
Supplemental File 1/ TableS8	VarRatio Left gene list
Supplemental File 1/ TableS9	ncNATDiffCor gene list
Supplemental File 1/ TableS10	association of DiffCor, VarRatio and ncNATDiffExp list with known pronostic factors
Supplemental File 1/ TableS11	cancer genes potentially regulated by their corresponding ncNAT
Supplemental File 1/ TableS12	RNASeq picard metrics
Supplemental File 1/ FigS13	picard metrics summary
Supplemental File 1/ FigS14	Data quality assessment : strand specificity
Supplemental File 1/ FigS15	Data quality assessment : MA-Plot
Supplemental File 1/ FigS16	Data quality assessment : boxplot of Cook's distances
Supplemental File 1/ FigS17	Count threshold description
Supplemental File 1/ FigS18	PCA
Supplemental File 1/ Additional Material and Methods	Additional Material and Methods

Supplemental File 1 /Figure S1: Validation of RNA-Seq.

A. Fold change distributions of genes, as determined by RNA-Seq, which are located in somatic copy-number alterations (amplifications or deletions), as determined by CGH. The distinct curves show a clear effect of the copy-number alterations on the gene expression (fold-changes). As expected, genes located in genomic amplified regions in the tumor showed increased expression, and conversely. **B.** Gene expression fold-changes between tumor and non-malignant tissues obtained in the current RNA-Seq study were compared to those described in an external Affymetrix micro-array dataset GSE65216. This comparison showed a global concordance of the results, with a Spearman correlation coefficient of 0.613 (p-value < 0.001). **C.** The relative expression of the protein coding ADAMTS9 and its ncNAT, ADAMTS9-AS2, in tumors and non-malignant tissues obtained by RNA sequencing and by RT-PCR were compared. The RT-qPCR values were normalized by the expression of the endogenous control gene B2M. [p-value <0.001 (***)].

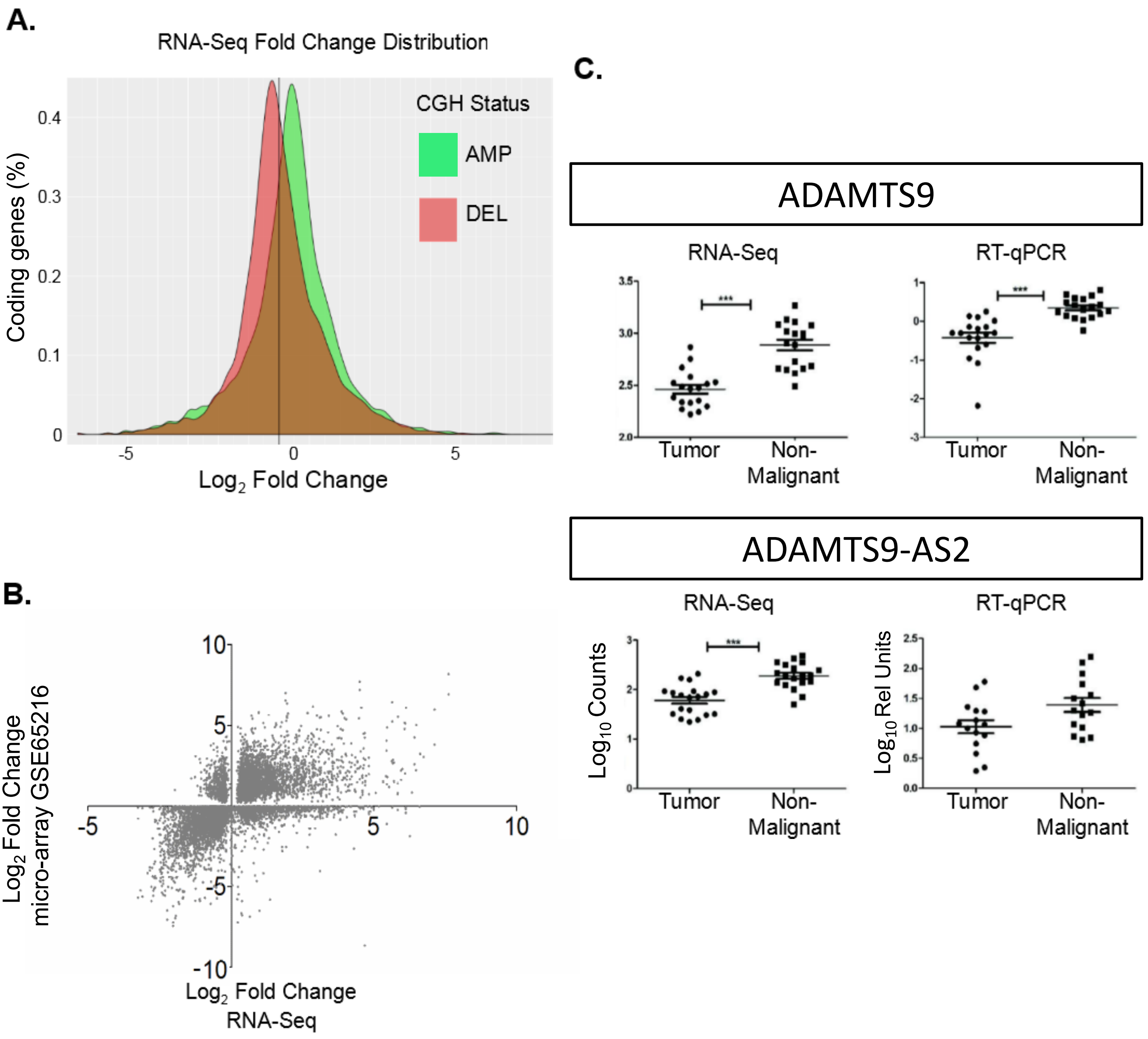

Supplemental File 1/ Table S2 : A GSEA analysis was conducted on GenePattern (http://genepattern.broadinstitute.org/gp/) on the Wenric & ElGuendi dataset and on the GSE65216 (Maire's cohort) dataset using the same parameters.The genes expressed in breast tumors were compared to the MSigDB c2ALLv. 6 database to search for gene set enrichment. The 100 first enriched gene sets in tumor were listed.

1026 gene sets were enriched in the Wenric & Elguendi dataset. Amoung them, 870 were also enriched in the GSE65216 dataset showing a 85% homology in gene sets enrichment in both dataset.

		Wenric & ElGuendi Dataset				GSE65216 Dataset			
**NAME**	**SIZE**	**ES**	**NES**	**NOM p-val**	**FDR q-val**	**ES**	**NES**	**NOM p-val**	**FDR q-val**
LIEN_BREAST_CARCINOMA_METAPLASTIC_VS_DUCTAL_DN	97	0,676	1,932	0,002	0,987	0,699	1,383	0,034	1
SMID_BREAST_CANCER_RELAPSE_IN_BRAIN_DN	73	0,678	1,921	0,002	0,581	0,674	1,339	0,079	1
VANTVEER_BREAST_CANCER_ESR1_UP	144	0,657	1,902	0,010	0,498	0,594	1,299	0,146	1
CHARAFE_BREAST_CANCER_LUMINAL_VS_MESENCHYMAL_UP	371	0,646	1,887	0,012	0,456	0,612	1,311	0,135	1
SMID_BREAST_CANCER_LUMINAL_B_UP	142	0,639	1,842	0,006	0,636	0,635	1,359	0,043	1
NIKOLSKY_BREAST_CANCER_1Q21_AMPLICON	34	0,752	1,839	0,009	0,548	0,543	1,144	0,384	1
COLDREN_GEFITINIB_RESISTANCE_DN	193	0,545	1,823	0,008	0,565	0,502	1,236	0,191	1
DOANE_BREAST_CANCER_ESR1_UP	96	0,642	1,812	0,018	0,560	0,749	1,406	0,016	1
AIGNER_ZEB1_TARGETS	30	0,671	1,793	0,012	0,616	0,565	1,244	0,221	1
PID_ATR_PATHWAY	38	0,667	1,780	0,018	0,637	0,557	1,163	0,357	1
REACTOME_DOUBLE_STRAND_BREAK_REPAIR	21	0,681	1,771	0,006	0,640	0,489	1,135	0,358	0,9892
YANG_BREAST_CANCER_ESR1_UP	33	0,685	1,764	0,014	0,628	0,688	1,383	0,051	1
KAUFFMANN_MELANOMA_RELAPSE_UP	56	0,694	1,763	0,023	0,584	0,649	1,230	0,269	1
ODONNELL_TFRC_TARGETS_DN	112	0,575	1,742	0,061	0,664	0,650	1,255	0,267	1
REACTOME_BASE_EXCISION_REPAIR	18	0,676	1,740	0,014	0,631				
NAKAYAMA_SOFT_TISSUE_TUMORS_PCA2_UP	83	0,617	1,740	0,035	0,592	0,731	1,332	0,134	1
LIM_MAMMARY_LUMINAL_MATURE_UP	93	0,517	1,738	0,023	0,571	0,510	1,225	0,191	1
LIM_MAMMARY_STEM_CELL_DN	366	0,478	1,713	0,048	0,674	0,467	1,175	0,268	1
CHARAFE_BREAST_CANCER_LUMINAL_VS_BASAL_UP	317	0,566	1,712	0,050	0,644	0,595	1,327	0,085	1
REACTOME_RESOLUTION_OF_AP_SITES_VIA_THE_MULTIPLE_NUCLEOTIDE_PATCH_REPLACEMENT_PATHWAY	17	0,665	1,709	0,017	0,627				
TURASHVILI_BREAST_DUCTAL_CARCINOMA_VS_LOBULAR_NORMAL_UP	62	0,586	1,706	0,030	0,614	0,729	1,350	0,031	1
FERREIRA_EWINGS_SARCOMA_UNSTABLE_VS_STABLE_UP	135	0,513	1,703	0,046	0,603	0,598	1,284	0,189	1
PID_FANCONI_PATHWAY	39	0,598	1,702	0,033	0,581	0,552	1,172	0,359	1
MUELLER_PLURINET	273	0,505	1,698	0,033	0,582	0,474	1,161	0,349	1
SMID_BREAST_CANCER_RELAPSE_IN_BONE_UP	82	0,550	1,697	0,014	0,559	0,611	1,336	0,070	1
ZHANG_TLX_TARGETS_DN	84	0,631	1,690	0,057	0,575	0,625	1,185	0,328	1
REACTOME_TRANSPORT_OF_MATURE_MRNA_DERIVED_FROM_AN_INTRONLESS_TRANSCRIPT	31	0,581	1,687	0,020	0,567	0,539	1,170	0,350	1
PUJANA_BRCA2_PCC_NETWORK	373	0,476	1,679	0,042	0,588	0,528	1,205	0,283	1
REACTOME_POST_TRANSLATIONAL_MODIFICATION_SYNTHESIS_OF_GPI_ANCHORED_PROTEINS	22	0,625	1,676	0,039	0,584	0,415	0,994	0,507	0,8658
FARMER_BREAST_CANCER_CLUSTER_6	16	0,761	1,675	0,025	0,566				
PUJANA_BRCA_CENTERED_NETWORK	106	0,558	1,675	0,037	0,549	0,559	1,197	0,316	1
REACTOME_PROCESSING_OF_CAPPED_INTRONLESS_PRE_MRNA	23	0,665	1,674	0,032	0,534	0,534	1,159	0,355	1
REN_BOUND_BY_E2F	53	0,723	1,674	0,023	0,520	0,660	1,252	0,199	1
REACTOME_G2_M_CHECKPOINTS	37	0,658	1,674	0,029	0,507	0,684	1,228	0,257	1
LIU_PROSTATE_CANCER_UP	77	0,531	1,673	0,041	0,494	0,431	1,117	0,329	0,9619
PUJANA_XPRSS_INT_NETWORK	150	0,536	1,668	0,047	0,501	0,558	1,226	0,268	1
REACTOME_ACTIVATION_OF_ATR_IN_RESPONSE_TO_REPLICATION_STRESS	31	0,668	1,664	0,033	0,505	0,699	1,256	0,224	1
REACTOME_DNA_REPAIR	94	0,481	1,663	0,029	0,494	0,440	1,183	0,322	1
CHANG_CYCLING_GENES	125	0,663	1,663	0,043	0,483	0,717	1,264	0,203	1
DUTERTRE_ESTRADIOL_RESPONSE_24HR_UP	287	0,609	1,657	0,060	0,495	0,662	1,294	0,191	1
PID_ATM_PATHWAY	33	0,543	1,657	0,039	0,484	0,533	1,178	0,317	1
NIELSEN_LIPOSARCOMA_DN	17	0,583	1,655	0,025	0,477	0,560	1,232	0,164	1
ROSTY_CERVICAL_CANCER_PROLIFERATION_CLUSTER	126	0,753	1,654	0,033	0,470	0,777	1,237	0,268	1
GOBERT_OLIGODENDROCYTE_DIFFERENTIATION_UP	481	0,484	1,653	0,059	0,464	0,502	1,169	0,329	1
ZHOU_CELL_CYCLE_GENES_IN_IR_RESPONSE_6HR	71	0,674	1,651	0,048	0,464	0,685	1,213	0,327	1
CHIANG_LIVER_CANCER_SUBCLASS_PROLIFERATION_UP	153	0,533	1,650	0,063	0,455	0,651	1,269	0,237	1
OXFORD_RALA_OR_RALB_TARGETS_UP	42	0,675	1,649	0,045	0,451	0,693	1,225	0,273	1
PID_BARD1_PATHWAY	26	0,644	1,649	0,047	0,441	0,478	1,137	0,387	0,9984
SARRIO_EPITHELIAL_MESENCHYMAL_TRANSITION_UP	153	0,544	1,642	0,052	0,454	0,632	1,237	0,256	1
REACTOME_CLEAVAGE_OF_GROWING_TRANSCRIPT_IN_THE_TERMINATION_REGION_	41	0,539	1,641	0,048	0,449	0,531	1,196	0,310	1
WAMUNYOKOLI_OVARIAN_CANCER_GRADES_1_2_UP	120	0,494	1,640	0,032	0,445	0,516	1,242	0,195	1
REACTOME_METABOLISM_OF_NON_CODING_RNA	47	0,539	1,638	0,040	0,444	0,548	1,233	0,291	1
LI_WILMS_TUMOR_ANAPLASTIC_UP	18	0,771	1,636	0,046	0,443				
WANG_METASTASIS_OF_BREAST_CANCER_ESR1_UP	19	0,720	1,633	0,029	0,446	0,713	1,258	0,161	1
RHODES_CANCER_META_SIGNATURE	59	0,645	1,630	0,043	0,446	0,638	1,196	0,342	1

STEIN_ESRRA_TARGETS_RESPONSIVE_TO_ESTROGEN_DN	32	0,626	1,629	0,031	0,442	0,717	1,325	0,116	1
LI_WILMS_TUMOR_VS_FETAL_KIDNEY_1_DN	151	0,541	1,626	0,057	0,443	0,593	1,249	0,211	1
KARAKAS_TGFB1_SIGNALING	16	0,619	1,622	0,029	0,449				
WU_APOPTOSIS_BY_CDKN1A_VIA_TP53	49	0,640	1,620	0,055	0,450	0,732	1,225	0,257	1
ODONNELL_TARGETS_OF_MYC_AND_TFRC_DN	41	0,640	1,618	0,077	0,447	0,643	1,113	0,398	0,9609
REACTOME_EXTENSION_OF_TELOMERES	27	0,646	1,617	0,042	0,443	0,613	1,196	0,293	1
BURTON_ADIPOGENESIS_PEAK_AT_24HR	38	0,645	1,611	0,076	0,458	0,746	1,264	0,223	1
KAUFFMANN_DNA_REPAIR_GENES	200	0,422	1,609	0,037	0,455	0,462	1,230	0,255	1
BONOME_OVARIAN_CANCER_POOR_SURVIVAL_DN	18	0,654	1,609	0,039	0,450	0,516	1,072	0,431	0,8826
KANG_DOXORUBICIN_RESISTANCE_UP	47	0,739	1,608	0,061	0,445	0,811	1,239	0,247	1
ZHANG_TLX_TARGETS_60HR_DN	246	0,509	1,604	0,085	0,451	0,590	1,227	0,272	1
BOYAULT_LIVER_CANCER_SUBCLASS_G123_UP	43	0,608	1,599	0,040	0,461	0,579	1,184	0,342	1
KONG_E2F3_TARGETS	87	0,659	1,599	0,062	0,455	0,734	1,284	0,235	1
FINETTI_BREAST_CANCERS_KINOME_BLUE	20	0,656	1,599	0,044	0,448	0,669	1,239	0,209	1
SANSOM_WNT_PATHWAY_REQUIRE_MYC	54	0,387	1,598	0,010	0,444	0,407	1,069	0,313	0,8806
EGUCHI_CELL_CYCLE_RB1_TARGETS	21	0,826	1,597	0,031	0,441	0,746	1,144	0,385	1
SU_TESTIS	65	0,501	1,596	0,049	0,437	0,550	1,216	0,268	1
KEGG_BASE_EXCISION_REPAIR	32	0,548	1,594	0,047	0,437	0,456	1,085	0,430	0,9083
REACTOME_FANCONI_ANEMIA_PATHWAY	16	0,634	1,590	0,053	0,443	0,480	1,122	0,358	0,9729
SONG_TARGETS_OF_IE86_CMV_PROTEIN	49	0,638	1,589	0,083	0,440	0,625	1,182	0,354	1
REACTOME_DNA_STRAND_ELONGATION	28	0,690	1,583	0,059	0,455	0,671	1,220	0,316	1
CROONQUIST_NRAS_SIGNALING_DN	68	0,671	1,582	0,073	0,453	0,728	1,210	0,292	1
TOMLINS_PROSTATE_CANCER_UP	34	0,538	1,578	0,066	0,457	0,473	1,136	0,376	0,9927
VANTVEER_BREAST_CANCER_METASTASIS_UP	50	0,533	1,576	0,069	0,460	0,538	1,166	0,257	1
ZHOU_CELL_CYCLE_GENES_IN_IR_RESPONSE_24HR	108	0,603	1,575	0,087	0,455	0,694	1,259	0,248	1
SOTIRIOU_BREAST_CANCER_GRADE_1_VS_3_UP	132	0,699	1,575	0,079	0,449	0,735	1,202	0,328	1
REACTOME_MEIOTIC_RECOMBINATION	44	0,573	1,574	0,057	0,449	0,577	1,236	0,263	1
BIOCARTA_ATRBRCA_PATHWAY	17	0,597	1,572	0,039	0,448	0,462	1,067	0,401	0,8784
MISSIAGLIA_REGULATED_BY_METHYLATION_DN	108	0,539	1,570	0,095	0,449	0,631	1,247	0,254	1
WHITFIELD_CELL_CYCLE_G1_S	116	0,448	1,568	0,060	0,450	0,381	1,024	0,439	0,8546
VERNELL_RETINOBLASTOMA_PATHWAY_UP	63	0,569	1,567	0,086	0,449	0,530	1,121	0,384	0,9671
HU_GENOTOXIC_DAMAGE_4HR	32	0,562	1,563	0,068	0,456	0,685	1,241	0,256	1
REACTOME_CELL_CYCLE_CHECKPOINTS	101	0,529	1,562	0,064	0,453	0,585	1,197	0,351	1
CHARAFE_BREAST_CANCER_BASAL_VS_MESENCHYMAL_UP	98	0,489	1,562	0,083	0,448	0,299	0,796	0,810	0,9066
REACTOME_NEP_NS2_INTERACTS_WITH_THE_CELLULAR_EXPORT_ MACHINERY	26	0,545	1,562	0,056	0,444	0,565	1,210	0,321	1
REACTOME_HOMOLOGOUS_RECOMBINATION_REPAIR_OF_REPLICA TION_INDEPENDENT_DOUBLE_STRAND_BREAKS	15	0,645	1,562	0,045	0,439				
YANG_BREAST_CANCER_ESR1_LASER_UP	28	0,600	1,558	0,051	0,444	0,678	1,229	0,226	1
KOBAYASHI_EGFR_SIGNALING_24HR_DN	227	0,554	1,558	0,110	0,439	0,662	1,262	0,236	1
REACTOME_ZINC_TRANSPORTERS	15	0,642	1,558	0,051	0,435				
REACTOME_MRNA_PROCESSING	146	0,446	1,556	0,066	0,437	0,459	1,213	0,292	1
CREIGHTON_ENDOCRINE_THERAPY_RESISTANCE_2	331	0,363	1,550	0,044	0,448	0,324	0,956	0,524	0,8668
PID_INTEGRIN5_PATHWAY	17	0,597	1,549	0,040	0,448				
WAMUNYOKOLI_OVARIAN_CANCER_LMP_UP	224	0,432	1,548	0,083	0,446	0,366	1,014	0,486	0,8593
BLUM_RESPONSE_TO_SALIRASIB_DN	316	0,444	1,548	0,100	0,442	0,553	1,252	0,170	1
BOYAULT_LIVER_CANCER_SUBCLASS_G12_UP	34	0,470	1,547	0,046	0,438	0,545	1,175	0,336	1

Supplemental File 1/ Figure S3 : Enrichment plots of several representative pathways that are differentially enriched in tumor and non-malignant adjacent tissues are presented showing comparable profiles in both Wenric & Elguendi dataset and GSE65216 datasets.

Wenric & ElGuendi dataset

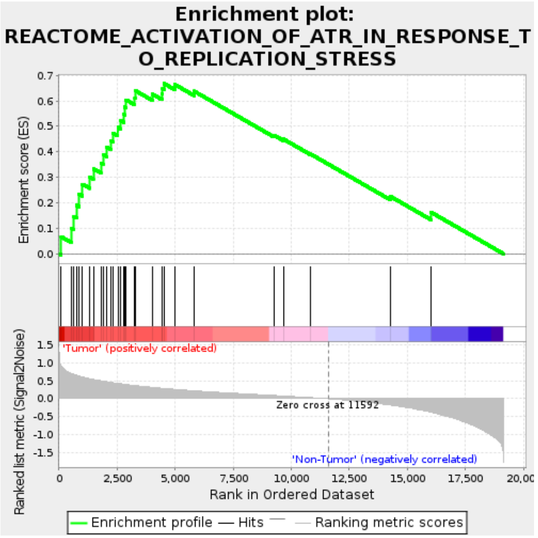

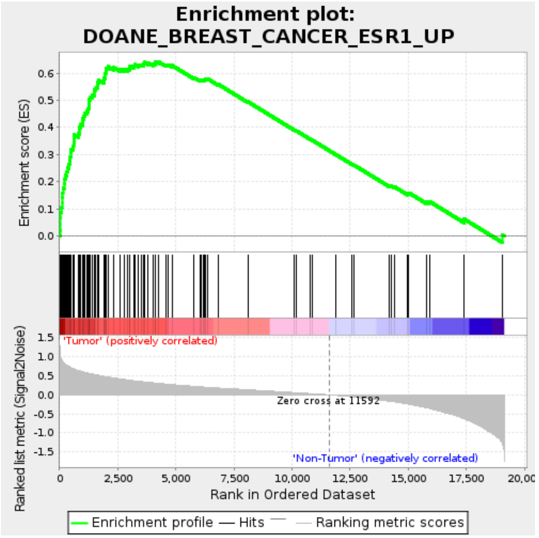

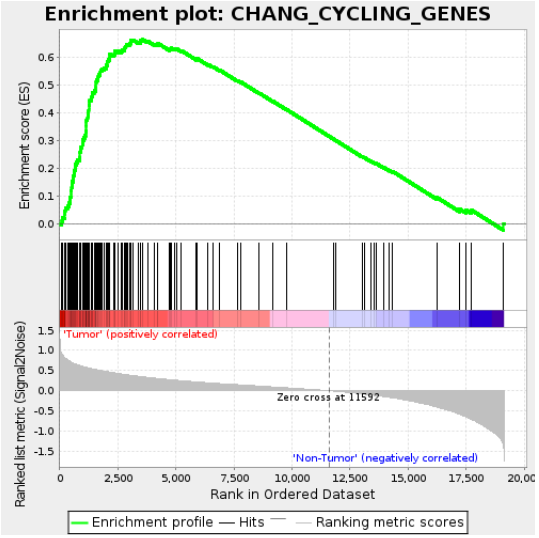

GSE65216 dataset (Maire's cohort)

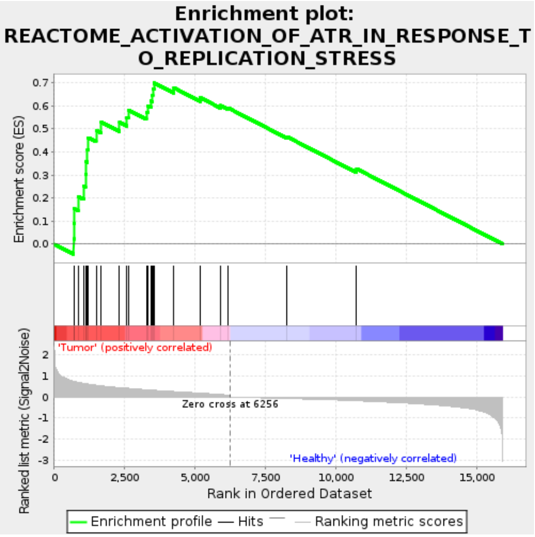

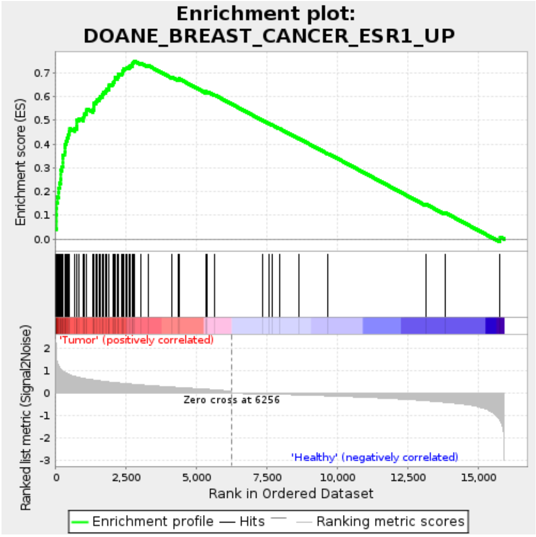

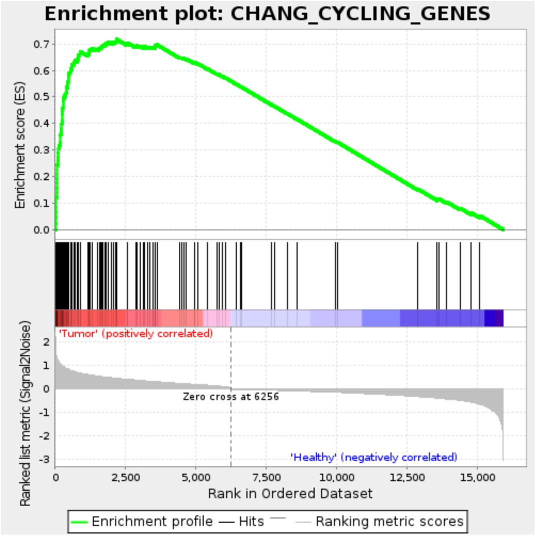

<u>Supplemental File 1 / Table S4</u> :

A. The complete list of 9632 PCT/NAT pairs contains 6494 unique PC gene and 8861 unique NAT gene

B. The subgroup of 4884 PCT/NAT pairs including only transcripts that are expressed both in the tumor and the healthy tissue of 7/22 patients contains 3247 unique PC gene and 4057 unique NAT genes

Transcripts overlap is distributed as follow :

A.

3888	PCT hold	1	overlapping NAT
1298	PCT hold	2	overlapping NAT
401	PCT hold	3	overlapping NAT
178	PCT hold	4	overlapping NAT
45	PCT hold	5	overlapping NAT
25	PCT hold	6	overlapping NAT
17	PCT hold	7	overlapping NAT
4	PCT hold	8	overlapping NAT
6	PCT hold	9	overlapping NAT

3888	NAT hold	1	overlapping PCT
528	NAT hold	2	overlapping PCT
71	NAT hold	3	overlapping PCT
17	NAT hold	4	overlapping PCT
5	NAT hold	5	overlapping PCT
1	NAT hold	6	overlapping PCT
2	NAT hold	7	overlapping PCT
2	NAT hold	8	overlapping PCT

B.

2633	PCT hold	1	overlapping NAT
581	PCT hold	2	overlapping NAT
100	PCT hold	3	overlapping NAT
23	PCT hold	4	overlapping NAT
12	PCT hold	5	overlapping NAT
2	PCT hold	7	overlapping NAT
1	PCT hold	9	overlapping NAT

2633	NAT hold	1	overlapping PCT
337	NAT hold	2	overlapping PCT
37	NAT hold	3	overlapping PCT
8	NAT hold	4	overlapping PCT
2	NAT hold	5	overlapping PCT

Supplemental File 1/ Figure S5. VarRatio threshold definition : frequencies of the log of varRatio values are plotted and one standard deviation is defined as threshold to select extreme varRatio as interesting PC/NAT gene pairs.

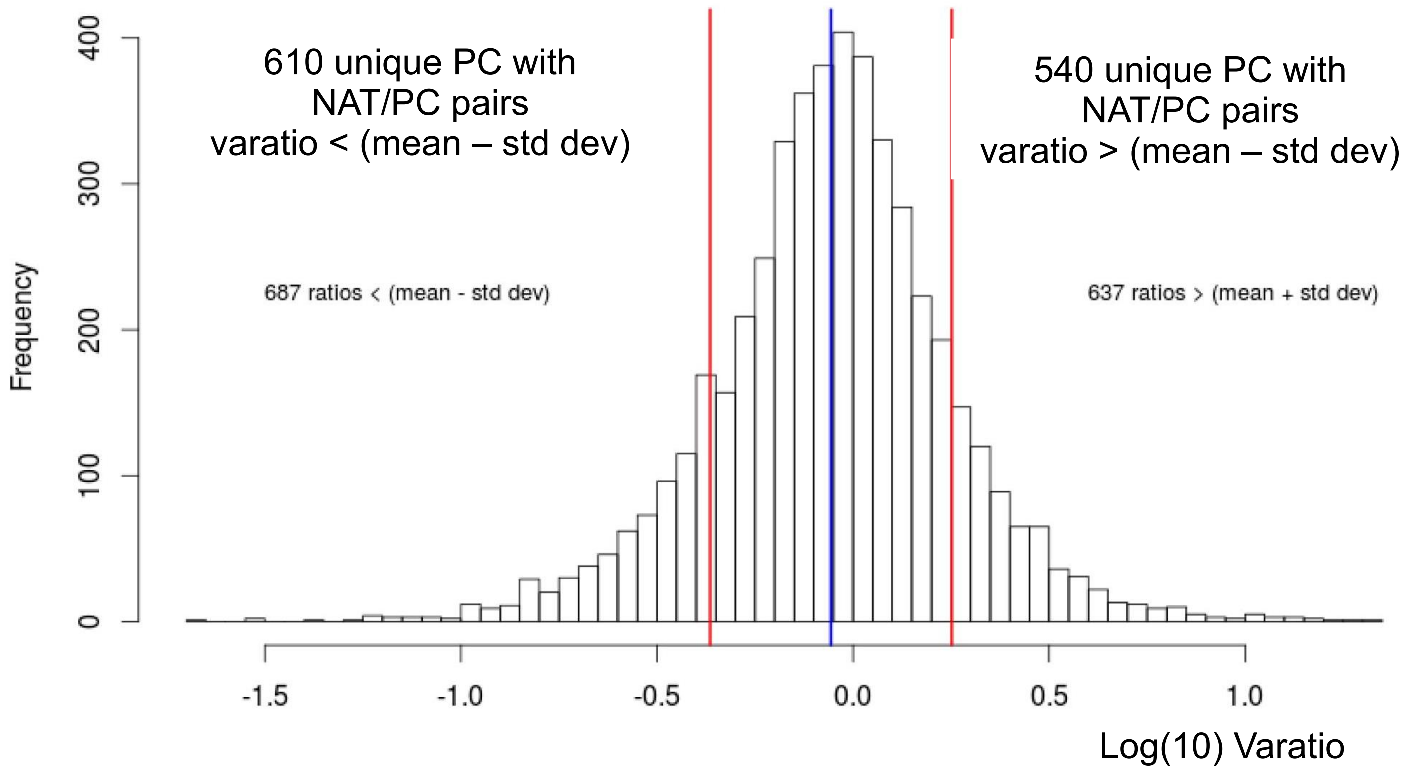

Supplemental File 1/Table S6 : DiffCorr list of ncNAT/PC pairs. In this list, PC expression is potentially regulated by their corresponding ncNAT. The copy number status of the genes is indicated.

Differential correlation analysis between pairs of protein-coding and antisense transcripts was performed with the DGCA software (v. 1.0.1) [PMID:27846853; McKenzie et al. BMC Systems Biology (2016) 10:106]. Pairs of protein coding/antisense genes whose correlation is significantly different between normal and tumor samples (adjusted p-value < 0.05) and whose correlation class differs between tumor and normal samples (ie. we removed the 0/0, +/+, -/- classes) have been selected. Pairs of genes where either the protein-coding or the antisense was expressed in less than 7 tumor samples or 7 healthy samples have been discarded.

Ensembl	Gene	Nb patients AMP	Nb patients DEL	Nb patients NEUTRAL	Majority
ENSG00000001617	SEMA3F	0	0	22	NEUTRAL
ENSG00000003402	CFLAR	0	0	22	NEUTRAL
ENSG00000004866	ST7	3	1	18	NEUTRAL
ENSG00000006007	GDE1	12	0	10	AMP
ENSG00000006451	RALA	4	1	17	NEUTRAL
ENSG00000006534	ALDH3B1	2	1	19	NEUTRAL
ENSG00000006837	CDKL3	0	2	20	NEUTRAL
ENSG00000007047	MARK4	0	1	21	NEUTRAL
ENSG00000008382	MPND	1	1	20	NEUTRAL
ENSG00000012048	BRCA1	1	4	17	NEUTRAL
ENSG00000012232	EXTL3	4	4	14	NEUTRAL
ENSG00000023287	RB1CC1	7	1	14	NEUTRAL
ENSG00000028277	POU2F2	0	1	21	NEUTRAL
ENSG00000029534	ANK1	7	3	12	NEUTRAL
ENSG00000031003	FAM13B	0	2	20	NEUTRAL
ENSG00000037757	MRI1	0	2	20	NEUTRAL
ENSG00000049283	EPN3	3	2	17	NEUTRAL
ENSG00000053254	FOXN3	0	1	21	NEUTRAL
ENSG00000054392	HHAT	15	0	7	AMP
ENSG00000055163	CYFIP2	0	2	20	NEUTRAL
ENSG00000058091	CDK14	4	0	18	NEUTRAL
ENSG00000058453	CROCC	1	3	18	NEUTRAL
ENSG00000061987	MON2	0	0	22	NEUTRAL
ENSG00000062485	CS	0	0	22	NEUTRAL
ENSG00000064309	CDON	0	7	15	NEUTRAL
ENSG00000064989	CALCRL	0	0	22	NEUTRAL
ENSG00000067715	SYT1	0	0	22	NEUTRAL
ENSG00000068489	PRR11	2	1	19	NEUTRAL
ENSG00000069020	MAST4	0	1	21	NEUTRAL
ENSG00000070193	FGF10	1	0	21	NEUTRAL
ENSG00000070731	ST6GALNAC2	1	2	19	NEUTRAL
ENSG00000071909	MYO3B	0	1	21	NEUTRAL
ENSG00000072736	NFATC3	1	13	8	DEL
ENSG00000075399	VPS9D1	1	12	9	DEL
ENSG00000076351	SLC46A1	1	2	19	NEUTRAL
ENSG00000076928	ARHGEF1	0	1	21	NEUTRAL
ENSG00000076944	STXBP2	1	1	20	NEUTRAL
ENSG00000078747	ITCH	3	0	19	NEUTRAL
ENSG00000078967	UBE2D4	4	1	17	NEUTRAL
ENSG00000079435	LIPE	0	1	21	NEUTRAL
ENSG00000080293	SCTR	0	0	22	NEUTRAL
ENSG00000081177	EXD2	0	1	21	NEUTRAL
ENSG00000082497	SERTAD4	15	0	7	AMP
ENSG00000082996	RNF13	1	1	20	NEUTRAL
ENSG00000085644	ZNF213	13	0	9	AMP
ENSG00000087460	GNAS	3	0	19	NEUTRAL
ENSG00000087884	AAMDC	4	2	16	NEUTRAL
ENSG00000088256	GNA11	0	2	20	NEUTRAL
ENSG00000089053	ANAPC5	0	0	22	NEUTRAL
ENSG00000089094	KDM2B	0	0	22	NEUTRAL
ENSG00000092841	MYL6	0	0	22	NEUTRAL

Ensembl	Gene	Nb patients AMP	Nb patients DEL	Nb patients NEUTRAL	Majority
ENSG00000095002	MSH2	0	0	22	NEUTRAL
ENSG00000099957	P2RX6	0	6	16	NEUTRAL
ENSG00000100036	SLC35E4	0	6	16	NEUTRAL
ENSG00000100100	PIK3IP1	0	6	16	NEUTRAL
ENSG00000100197	CYP2D6	0	6	16	NEUTRAL
ENSG00000100479	POLE2	1	0	21	NEUTRAL
ENSG00000100522	GNPNAT1	1	0	21	NEUTRAL
ENSG00000100934	SEC23A	0	0	22	NEUTRAL
ENSG00000101204	CHRNA4	3	0	19	NEUTRAL
ENSG00000101493	ZNF516	1	1	20	NEUTRAL
ENSG00000101745	ANKRD12	1	2	19	NEUTRAL
ENSG00000101773	RBBP8	1	2	19	NEUTRAL
ENSG00000102606	ARHGEF7	0	3	19	NEUTRAL
ENSG00000102974	CTCF	1	13	8	DEL
ENSG00000103335	PIEZO1	2	12	8	DEL
ENSG00000103342	GSPT1	12	0	10	AMP
ENSG00000103351	CLUAP1	13	0	9	AMP
ENSG00000103591	AAGAB	0	3	19	NEUTRAL
ENSG00000104044	OCA2	0	0	22	NEUTRAL
ENSG00000104154	SLC30A4	0	3	19	NEUTRAL
ENSG00000104313	EYA1	8	0	14	NEUTRAL
ENSG00000104497	SNX16	9	0	13	NEUTRAL
ENSG00000104517	UBR5	9	0	13	NEUTRAL
ENSG00000104763	ASAH1	4	7	11	NEUTRAL
ENSG00000104901	DKKL1	1	1	20	NEUTRAL
ENSG00000104960	PTOV1	1	1	20	NEUTRAL
ENSG00000105323	HNRNPUL1	0	1	21	NEUTRAL
ENSG00000105364	MRPL4	1	1	20	NEUTRAL
ENSG00000105371	ICAM4	1	1	20	NEUTRAL
ENSG00000105443	CYTH2	0	1	21	NEUTRAL
ENSG00000105707	HPN	0	1	21	NEUTRAL
ENSG00000105778	AVL9	4	1	17	NEUTRAL
ENSG00000106018	VIPR2	4	0	18	NEUTRAL
ENSG00000107443	CCNJ	1	1	20	NEUTRAL
ENSG00000108244	KRT23	1	3	18	NEUTRAL
ENSG00000108439	PNPO	3	2	17	NEUTRAL
ENSG00000108823	SGCA	3	2	17	NEUTRAL
ENSG00000108924	HLF	2	2	18	NEUTRAL
ENSG00000108950	FAM20A	2	1	19	NEUTRAL
ENSG00000109586	GALNT7	0	1	21	NEUTRAL
ENSG00000109787	KLF3	0	0	22	NEUTRAL
ENSG00000109819	PPARGC1A	0	0	22	NEUTRAL
ENSG00000110721	CHKA	2	0	20	NEUTRAL
ENSG00000111224	PARP11	0	1	21	NEUTRAL
ENSG00000111269	CREBL2	0	1	21	NEUTRAL
ENSG00000111405	ENDOU	0	0	22	NEUTRAL
ENSG00000111725	PRKAB1	0	0	22	NEUTRAL
ENSG00000111877	MCM9	0	6	16	NEUTRAL
ENSG00000112276	BVES	0	6	16	NEUTRAL
ENSG00000112303	VNN2	0	5	17	NEUTRAL
ENSG00000112319	EYA4	0	5	17	NEUTRAL
ENSG00000112406	HECA	0	6	16	NEUTRAL
ENSG00000112659	CUL9	0	0	22	NEUTRAL
ENSG00000113161	HMGCR	0	1	21	NEUTRAL
ENSG00000113522	RAD50	0	2	20	NEUTRAL
ENSG00000114113	RBP2	1	1	20	NEUTRAL
ENSG00000114473	IQCG	1	1	20	NEUTRAL
ENSG00000114670	NEK11	1	2	19	NEUTRAL
ENSG00000115641	FHL2	0	0	22	NEUTRAL
ENSG00000115866	DARS	1	1	20	NEUTRAL
ENSG00000115896	PLCL1	0	0	22	NEUTRAL
ENSG00000116032	GRIN3B	0	2	20	NEUTRAL

Ensembl	Gene	Nb patients AMP	Nb patients DEL	Nb patients NEUTRAL	Majority
ENSG00000116062	MSH6	0	0	22	NEUTRAL
ENSG00000116183	PAPPA2	14	0	8	AMP
ENSG00000116786	PLEKHM2	1	3	18	NEUTRAL
ENSG00000116833	NR5A2	14	0	8	AMP
ENSG00000117602	RCAN3	1	4	17	NEUTRAL
ENSG00000117682	DHDDS	1	4	17	NEUTRAL
ENSG00000117983	MUC5B	2	2	18	NEUTRAL
ENSG00000119414	PPP6C	0	0	22	NEUTRAL
ENSG00000119715	ESRRB	0	1	21	NEUTRAL
ENSG00000120093	HOXB3	3	2	17	NEUTRAL
ENSG00000120645	IQSEC3	0	0	22	NEUTRAL
ENSG00000120725	SIL1	0	2	20	NEUTRAL
ENSG00000120833	SOCS2	0	0	22	NEUTRAL
ENSG00000120896	SORBS3	4	7	11	NEUTRAL
ENSG00000121039	RDH10	8	0	14	NEUTRAL
ENSG00000121073	SLC35B1	3	2	17	NEUTRAL
ENSG00000121104	FAM117A	3	2	17	NEUTRAL
ENSG00000121316	PLBD1	0	1	21	NEUTRAL
ENSG00000121361	KCNJ8	0	1	21	NEUTRAL
ENSG00000123094	RASSF8	0	0	22	NEUTRAL
ENSG00000123388	HOXC11	0	0	22	NEUTRAL
ENSG00000123552	USP45	0	6	16	NEUTRAL
ENSG00000123689	G0S2	15	0	7	AMP
ENSG00000124006	OBSL1	0	0	22	NEUTRAL
ENSG00000124813	RUNX2	0	0	22	NEUTRAL
ENSG00000125337	KIF25	0	5	17	NEUTRAL
ENSG00000126457	PRMT1	1	1	20	NEUTRAL
ENSG00000126733	DACH2	2	2	18	NEUTRAL
ENSG00000127948	POR	3	0	19	NEUTRAL
ENSG00000128709	HOXD9	0	1	21	NEUTRAL
ENSG00000128881	TTBK2	0	3	19	NEUTRAL
ENSG00000128908	INO80	0	3	19	NEUTRAL
ENSG00000129255	MPDU1	0	5	17	NEUTRAL
ENSG00000129480	DTD2	1	0	21	NEUTRAL
ENSG00000129493	HEATR5A	1	0	21	NEUTRAL
ENSG00000129595	EPB41L4A	0	2	20	NEUTRAL
ENSG00000130167	TSPAN16	1	1	20	NEUTRAL
ENSG00000130768	SMPDL3B	1	4	17	NEUTRAL
ENSG00000131378	RFTN1	0	0	22	NEUTRAL
ENSG00000131504	DIAPH1	0	2	20	NEUTRAL
ENSG00000131620	ANO1	6	0	16	NEUTRAL
ENSG00000131951	LRRC9	0	1	21	NEUTRAL
ENSG00000132321	IQCA1	0	1	21	NEUTRAL
ENSG00000132326	PER2	0	1	21	NEUTRAL
ENSG00000132359	RAP1GAP2	0	5	17	NEUTRAL
ENSG00000132846	ZBED3	0	1	21	NEUTRAL
ENSG00000132970	WASF3	0	3	19	NEUTRAL
ENSG00000133392	MYH11	12	0	10	AMP
ENSG00000133466	C1QTNF6	0	6	16	NEUTRAL
ENSG00000133657	ATP13A3	1	1	20	NEUTRAL
ENSG00000133706	LARS	0	2	20	NEUTRAL
ENSG00000134146	DPH6	0	2	20	NEUTRAL
ENSG00000134283	PPHLN1	0	0	22	NEUTRAL
ENSG00000134318	ROCK2	0	0	22	NEUTRAL
ENSG00000134504	KCTD1	1	1	20	NEUTRAL
ENSG00000134873	CLDN10	0	3	19	NEUTRAL
ENSG00000135018	UBQLN1	0	1	21	NEUTRAL
ENSG00000135100	HNF1A	0	0	22	NEUTRAL
ENSG00000135250	SRPK2	3	1	18	NEUTRAL
ENSG00000135269	TES	3	1	18	NEUTRAL
ENSG00000135314	KHDC1	0	4	18	NEUTRAL
ENSG00000135406	PRPH	0	0	22	NEUTRAL

Ensembl	Gene	Nb patients AMP	Nb patients DEL	Nb patients NEUTRAL	Majority
ENSG00000135898	GPR55	0	1	21	NEUTRAL
ENSG00000136068	FLNB	0	0	22	NEUTRAL
ENSG00000136153	LMO7	0	4	18	NEUTRAL
ENSG00000136379	ABHD17C	0	2	20	NEUTRAL
ENSG00000136908	DPM2	0	0	22	NEUTRAL
ENSG00000137494	ANKRD42	1	4	17	NEUTRAL
ENSG00000137500	CCDC90B	1	4	17	NEUTRAL
ENSG00000137571	SLCO5A1	8	0	14	NEUTRAL
ENSG00000137766	UNC13C	0	3	19	NEUTRAL
ENSG00000138185	ENTPD1	1	1	20	NEUTRAL
ENSG00000138378	STAT4	0	0	22	NEUTRAL
ENSG00000138640	FAM13A	0	1	21	NEUTRAL
ENSG00000138698	RAP1GDS1	0	1	21	NEUTRAL
ENSG00000139146	FAM60A	0	0	22	NEUTRAL
ENSG00000139151	PLCZ1	0	1	21	NEUTRAL
ENSG00000139193	CD27	0	1	21	NEUTRAL
ENSG00000139304	PTPRQ	0	0	22	NEUTRAL
ENSG00000139926	FRMD6	1	0	21	NEUTRAL
ENSG00000140455	USP3	0	3	19	NEUTRAL
ENSG00000140459	CYP11A1	0	2	20	NEUTRAL
ENSG00000140479	PCSK6	0	0	22	NEUTRAL
ENSG00000140526	ABHD2	0	2	20	NEUTRAL
ENSG00000140836	ZFHX3	1	13	8	DEL
ENSG00000140955	ADAD2	1	12	9	DEL
ENSG00000141013	GAS8	1	9	12	NEUTRAL
ENSG00000141161	UNC45B	1	3	18	NEUTRAL
ENSG00000141293	SKAP1	3	2	17	NEUTRAL
ENSG00000141434	MEP1B	2	1	19	NEUTRAL
ENSG00000141576	RNF157	1	2	19	NEUTRAL
ENSG00000142192	APP	2	0	20	NEUTRAL
ENSG00000142233	NTN5	1	1	20	NEUTRAL
ENSG00000143164	DCAF6	14	0	8	AMP
ENSG00000143376	SNX27	15	0	7	AMP
ENSG00000143418	CERS2	15	0	7	AMP
ENSG00000143641	GALNT2	15	0	7	AMP
ENSG00000143819	EPHX1	15	0	7	AMP
ENSG00000143845	ETNK2	16	0	6	AMP
ENSG00000144283	PKP4	0	1	21	NEUTRAL
ENSG00000144724	PTPRG	0	0	22	NEUTRAL
ENSG00000144736	SHQ1	0	0	22	NEUTRAL
ENSG00000145244	CORIN	0	0	22	NEUTRAL
ENSG00000145335	SNCA	0	1	21	NEUTRAL
ENSG00000146063	TRIM41	0	1	21	NEUTRAL
ENSG00000147419	CCDC25	4	5	13	NEUTRAL
ENSG00000147852	VLDLR	0	0	22	NEUTRAL
ENSG00000147883	CDKN2B	0	0	22	NEUTRAL
ENSG00000147889	CDKN2A	0	0	22	NEUTRAL
ENSG00000148219	ASTN2	0	0	22	NEUTRAL
ENSG00000149212	SESN3	1	5	16	NEUTRAL
ENSG00000149346	SLX4IP	4	0	18	NEUTRAL
ENSG00000149571	KIRREL3	0	7	15	NEUTRAL
ENSG00000150527	CTAGE5	0	0	22	NEUTRAL
ENSG00000150995	ITPR1	0	1	21	NEUTRAL
ENSG00000151092	NGLY1	0	0	22	NEUTRAL
ENSG00000151208	DLG5	1	0	21	NEUTRAL
ENSG00000151247	EIF4E	0	1	21	NEUTRAL
ENSG00000151948	GLT1D1	0	0	22	NEUTRAL
ENSG00000151952	TMEM132D	0	0	22	NEUTRAL
ENSG00000152591	DSPP	0	1	21	NEUTRAL
ENSG00000153002	CPB1	1	1	20	NEUTRAL
ENSG00000153956	CACNA2D1	3	0	19	NEUTRAL
ENSG00000154330	PGM5	0	0	22	NEUTRAL

Ensembl	Gene	Nb patients AMP	Nb patients DEL	Nb patients NEUTRAL	Majority
ENSG00000155096	AZIN1	9	0	13	NEUTRAL
ENSG00000155324	GRAMD3	0	2	20	NEUTRAL
ENSG00000155545	MIER3	0	0	22	NEUTRAL
ENSG00000155657	TTN	0	1	21	NEUTRAL
ENSG00000155714	PDZD9	12	0	10	AMP
ENSG00000155816	FMN2	14	0	8	AMP
ENSG00000155962	CLIC2	2	2	18	NEUTRAL
ENSG00000156697	UTP14A	2	2	18	NEUTRAL
ENSG00000156958	GALK2	0	3	19	NEUTRAL
ENSG00000157150	TIMP4	0	0	22	NEUTRAL
ENSG00000157778	PSMG3	3	1	18	NEUTRAL
ENSG00000158104	HPD	0	0	22	NEUTRAL
ENSG00000158486	DNAH3	12	0	10	AMP
ENSG00000158717	RNF166	2	12	8	DEL
ENSG00000158828	PINK1	1	3	18	NEUTRAL
ENSG00000159197	KCNE2	2	0	20	NEUTRAL
ENSG00000159251	ACTC1	0	2	20	NEUTRAL
ENSG00000159658	EFCAB14	0	2	20	NEUTRAL
ENSG00000160226	C21orf2	2	0	20	NEUTRAL
ENSG00000160439	RDH13	2	0	20	NEUTRAL
ENSG00000160551	TAOK1	1	2	19	NEUTRAL
ENSG00000160691	SHC1	15	0	7	AMP
ENSG00000160753	RUSC1	15	0	7	AMP
ENSG00000160888	IER2	0	2	20	NEUTRAL
ENSG00000160932	LY6E	8	1	13	NEUTRAL
ENSG00000161664	ASB16	1	3	18	NEUTRAL
ENSG00000162365	CYP4A22	0	2	20	NEUTRAL
ENSG00000162383	SLC1A7	0	2	20	NEUTRAL
ENSG00000162430	SEPN1	1	4	17	NEUTRAL
ENSG00000162460	TMEM82	1	3	18	NEUTRAL
ENSG00000162595	DIRAS3	0	1	21	NEUTRAL
ENSG00000162641	AKNAD1	0	2	20	NEUTRAL
ENSG00000162771	FAM71A	15	0	7	AMP
ENSG00000162873	KLHDC8A	16	0	6	AMP
ENSG00000163171	CDC42EP3	0	0	22	NEUTRAL
ENSG00000163348	PYGO2	15	0	7	AMP
ENSG00000163357	DCST1	15	0	7	AMP
ENSG00000164032	H2AFZ	0	1	21	NEUTRAL
ENSG00000164100	NDST3	0	1	21	NEUTRAL
ENSG00000164125	FAM198B	0	1	21	NEUTRAL
ENSG00000164176	EDIL3	0	1	21	NEUTRAL
ENSG00000164292	RHOBTB3	0	2	20	NEUTRAL
ENSG00000164398	ACSL6	0	2	20	NEUTRAL
ENSG00000164574	GALNT10	0	2	20	NEUTRAL
ENSG00000164647	STEAP1	4	0	18	NEUTRAL
ENSG00000164930	FZD6	9	0	13	NEUTRAL
ENSG00000165006	UBAP1	0	0	22	NEUTRAL
ENSG00000165046	LETM2	9	1	12	NEUTRAL
ENSG00000165092	ALDH1A1	0	1	21	NEUTRAL
ENSG00000165591	FAAH2	2	2	18	NEUTRAL
ENSG00000165646	SLC18A2	0	0	22	NEUTRAL
ENSG00000165678	GHITM	1	1	20	NEUTRAL
ENSG00000165895	ARHGAP42	0	5	17	NEUTRAL
ENSG00000166002	SMCO4	1	5	16	NEUTRAL
ENSG00000166006	KCNC2	0	0	22	NEUTRAL
ENSG00000166145	SPINT1	0	3	19	NEUTRAL
ENSG00000166435	XRRA1	3	1	18	NEUTRAL
ENSG00000166444	ST5	1	2	19	NEUTRAL
ENSG00000166527	CLEC4D	0	1	21	NEUTRAL
ENSG00000166801	FAM111A	1	1	20	NEUTRAL
ENSG00000166923	GREM1	0	2	20	NEUTRAL
ENSG00000166946	CCNDBP1	0	3	19	NEUTRAL

Ensembl	Gene	Nb patients AMP	Nb patients DEL	Nb patients NEUTRAL	Majority
ENSG00000166947	EPB42	0	3	19	NEUTRAL
ENSG00000166960	CCDC178	2	1	19	NEUTRAL
ENSG00000167216	KATNAL2	2	2	18	NEUTRAL
ENSG00000167880	EVPL	1	2	19	NEUTRAL
ENSG00000167889	MGAT5B	1	2	19	NEUTRAL
ENSG00000167972	ABCA3	12	0	10	AMP
ENSG00000168040	FADD	6	0	16	NEUTRAL
ENSG00000168275	COA6	15	0	7	AMP
ENSG00000168394	TAP1	0	0	22	NEUTRAL
ENSG00000168497	SDPR	0	1	21	NEUTRAL
ENSG00000168517	HEXIM2	2	3	17	NEUTRAL
ENSG00000168594	ADAM29	0	1	21	NEUTRAL
ENSG00000168675	LDLRAD4	1	1	20	NEUTRAL
ENSG00000169032	MAP2K1	0	3	19	NEUTRAL
ENSG00000169067	ACTBL2	0	1	21	NEUTRAL
ENSG00000169122	FAM110B	8	0	14	NEUTRAL
ENSG00000169594	BNC1	0	2	20	NEUTRAL
ENSG00000170275	CRTAP	0	0	22	NEUTRAL
ENSG00000170442	KRT86	0	0	22	NEUTRAL
ENSG00000170579	DLGAP1	1	2	19	NEUTRAL
ENSG00000171735	CAMTA1	1	2	19	NEUTRAL
ENSG00000171824	EXOSC10	1	3	18	NEUTRAL
ENSG00000171840	NINJ2	0	1	21	NEUTRAL
ENSG00000171953	ATPAF2	0	5	17	NEUTRAL
ENSG00000172164	SNTB1	9	0	13	NEUTRAL
ENSG00000172380	GNG12	0	1	21	NEUTRAL
ENSG00000172493	AFF1	0	1	21	NEUTRAL
ENSG00000173261	PLAC8L1	0	2	20	NEUTRAL
ENSG00000173818	ENDOV	1	2	19	NEUTRAL
ENSG00000173917	HOXB2	3	2	17	NEUTRAL
ENSG00000174255	ZNF80	2	1	19	NEUTRAL
ENSG00000174586	ZNF497	0	0	22	NEUTRAL
ENSG00000174989	FBXW8	0	0	22	NEUTRAL
ENSG00000174996	KLC2	3	3	16	NEUTRAL
ENSG00000175065	DSG4	2	1	19	NEUTRAL
ENSG00000175582	RAB6A	3	1	18	NEUTRAL
ENSG00000175600	SUGCT	4	1	17	NEUTRAL
ENSG00000175664	TEX26	0	3	19	NEUTRAL
ENSG00000175809	ZNF645	2	2	18	NEUTRAL
ENSG00000175899	A2M	0	1	21	NEUTRAL
ENSG00000176148	TCP11L1	3	1	18	NEUTRAL
ENSG00000176393	RNPEP	15	0	7	AMP
ENSG00000176406	RIMS2	9	0	13	NEUTRAL
ENSG00000176595	KBTBD11	3	6	13	NEUTRAL
ENSG00000176720	BOK	0	1	21	NEUTRAL
ENSG00000176912	C18orf56	1	2	19	NEUTRAL
ENSG00000177119	ANO6	0	0	22	NEUTRAL
ENSG00000177191	B3GNT8	0	1	21	NEUTRAL
ENSG00000177479	ARIH2	0	0	22	NEUTRAL
ENSG00000177694	NAALADL2	1	1	20	NEUTRAL
ENSG00000178307	TMEM11	0	5	17	NEUTRAL
ENSG00000178363	CALML3	0	1	21	NEUTRAL
ENSG00000179165	PXT1	0	1	21	NEUTRAL
ENSG00000179588	ZFPM1	1	12	9	DEL
ENSG00000180479	ZNF571	0	1	21	NEUTRAL
ENSG00000180694	TMEM64	9	0	13	NEUTRAL
ENSG00000180828	BHLHE22	8	0	14	NEUTRAL
ENSG00000180957	PITPNB	0	6	16	NEUTRAL
ENSG00000181085	MAPK15	8	1	13	NEUTRAL
ENSG00000182149	IST1	1	13	8	DEL
ENSG00000182575	NXPH3	3	2	17	NEUTRAL
ENSG00000182667	NTM	0	7	15	NEUTRAL

Ensembl	Gene	Nb patients AMP	Nb patients DEL	Nb patients NEUTRAL	Majority
ENSG00000182704	TSKU	3	2	17	NEUTRAL
ENSG00000182742	HOXB4	3	2	17	NEUTRAL
ENSG00000182979	MTA1	0	0	22	NEUTRAL
ENSG00000183090	FREM3	0	1	21	NEUTRAL
ENSG00000183092	BEGAIN	0	1	21	NEUTRAL
ENSG00000183248	CTD-3193O13.9	1	1	20	NEUTRAL
ENSG00000183273	CCDC60	0	0	22	NEUTRAL
ENSG00000184389	A3GALT2	1	4	17	NEUTRAL
ENSG00000184743	ATL3	1	4	17	NEUTRAL
ENSG00000184937	WT1	3	1	18	NEUTRAL
ENSG00000185619	PCGF3	0	0	22	NEUTRAL
ENSG00000185787	MORF4L1	0	2	20	NEUTRAL
ENSG00000186834	HEXIM1	2	3	17	NEUTRAL
ENSG00000187391	MAGI2	3	0	19	NEUTRAL
ENSG00000187535	IFT140	12	0	10	AMP
ENSG00000187824	TMEM220	0	5	17	NEUTRAL
ENSG00000188107	EYS	0	3	19	NEUTRAL
ENSG00000188316	ENO4	0	0	22	NEUTRAL
ENSG00000188343	FAM92A1	9	0	13	NEUTRAL
ENSG00000188610	FAM72B	0	0	22	NEUTRAL
ENSG00000188649	CC2D2B	1	1	20	NEUTRAL
ENSG00000188897	CTD-3088G3.8	13	0	9	AMP
ENSG00000188910	GJB3	1	4	17	NEUTRAL
ENSG00000189056	RELN	3	1	18	NEUTRAL
ENSG00000189319	FAM53B	0	0	22	NEUTRAL
ENSG00000196277	GRM7	0	1	21	NEUTRAL
ENSG00000196465	MYL6B	0	0	22	NEUTRAL
ENSG00000196535	MYO18A	1	2	19	NEUTRAL
ENSG00000196648	GOLGA6L20	0	2	20	NEUTRAL
ENSG00000196684	HSH2D	1	1	20	NEUTRAL
ENSG00000197147	LRRC8B	0	1	21	NEUTRAL
ENSG00000197415	VEPH1	1	2	19	NEUTRAL
ENSG00000197576	HOXA4	4	1	17	NEUTRAL
ENSG00000197584	KCNMB2	1	1	20	NEUTRAL
ENSG00000197620	CXorf40A	2	2	18	NEUTRAL
ENSG00000197647	ZNF433	0	2	20	NEUTRAL
ENSG00000197912	SPG7	1	12	9	DEL
ENSG00000197930	ERO1L	1	0	21	NEUTRAL
ENSG00000197977	ELOVL2	0	1	21	NEUTRAL
ENSG00000198046	ZNF667	2	0	20	NEUTRAL
ENSG00000198363	ASPH	8	0	14	NEUTRAL
ENSG00000198382	UVRAG	2	1	19	NEUTRAL
ENSG00000198700	IPO9	15	0	7	AMP
ENSG00000198780	FAM169A	0	1	21	NEUTRAL
ENSG00000198838	RYR3	0	2	20	NEUTRAL
ENSG00000198839	ZNF277	3	1	18	NEUTRAL
ENSG00000198948	MFAP3L	0	1	21	NEUTRAL
ENSG00000204296	C6orf10	0	0	22	NEUTRAL
ENSG00000204577	LILRB3	2	0	20	NEUTRAL
ENSG00000205212	CCDC144NL	0	5	17	NEUTRAL
ENSG00000205277	MUC12	4	0	18	NEUTRAL
ENSG00000205593	DENND6B	0	6	16	NEUTRAL
ENSG00000205629	LCMT1	12	1	9	AMP
ENSG00000205981	DNAJC19	1	1	20	NEUTRAL
ENSG00000213088	DARC	14	0	8	AMP
ENSG00000213160	KLHL23	0	2	20	NEUTRAL
ENSG00000213988	ZNF90	0	1	21	NEUTRAL
ENSG00000214188	ST7-OT4	3	1	18	NEUTRAL
ENSG00000215421	ZNF407	1	1	20	NEUTRAL
ENSG00000221970	OR2A1	4	2	16	NEUTRAL
ENSG00000223501	VPS52	0	0	22	NEUTRAL
ENSG00000225190	PLEKHM1	2	3	17	NEUTRAL

Ensembl	Gene	Nb patients AMP	Nb patients DEL	Nb patients NEUTRAL	Majority
ENSG00000226321	AC104809.3	0	1	21	NEUTRAL
ENSG00000232040	SCAND3	0	0	22	NEUTRAL
ENSG00000235098	ANKRD65	1	2	19	NEUTRAL
ENSG00000239605	C2orf61	0	0	22	NEUTRAL
ENSG00000242259	C22orf39	0	6	16	NEUTRAL
ENSG00000243414	TICAM2	0	2	20	NEUTRAL
ENSG00000245848	CEBPA	0	0	22	NEUTRAL
ENSG00000253293	HOXA10	4	1	17	NEUTRAL
ENSG00000255872	RP11-613M10.9	0	0	22	NEUTRAL
ENSG00000258740	RP11-293M10.1	0	1	21	NEUTRAL
ENSG00000260456	C16orf95	1	12	9	DEL
ENSG00000262039	RP11-81K2.1	3	2	17	NEUTRAL
ENSG00000268080	AC016885.1	9	0	13	NEUTRAL
ENSG00000268423	AC011551.3	0	1	21	NEUTRAL
ENSG00000268628	AL121761.2	4	0	18	NEUTRAL
ENSG00000269545	CTD-3138B18.4	0	0	22	NEUTRAL
ENSG00000269948	RP11-248J23.6	1	1	20	NEUTRAL
ENSG00000272325	NUDT3	0	0	22	NEUTRAL

Supplemental File 1 /Table S7 : Varatio-right list of ncNAT/PC pairs. In this list, PC expression is potentially regulated by their corresponding NAT. The copy number status was indiquated

The read counts varRatio has been calculated as described in the main manuscript. Pairs of NAT/PC genes corresponding to righttmost values of the varRatio distribution have been selected by applying a threshold (mean ± standard deviation) to the log-transformed distribution of the varRatios (Supplemental File 1/Table S5). Pairs of genes where either the protein-coding or the antisense was expressed in less than 7 tumor samples or 7 healthy samples have been discarded.

Ensembl	Gene	Nb patients AMP	Nb patients DEL	Nb patients NEUTRAL	Majority
ENSG00000166317	SYNPO2L	0	0	22	NEUTRAL
ENSG00000169067	ACTBL2	0	1	21	NEUTRAL
ENSG00000146197	SCUBE3	0	0	22	NEUTRAL
ENSG00000165646	SLC18A2	0	0	22	NEUTRAL
ENSG00000171595	DNAI2	1	1	20	NEUTRAL
ENSG00000144485	HES6	0	1	21	NEUTRAL
ENSG00000158717	RNF166	2	12	8	DEL
ENSG00000113161	HMGCR	0	1	21	NEUTRAL
ENSG00000158623	COPG2	3	1	18	NEUTRAL
ENSG00000186160	CYP4Z1	0	2	20	NEUTRAL
ENSG00000196611	MMP1	0	5	17	NEUTRAL
ENSG00000132031	MATN3	0	0	22	NEUTRAL
ENSG00000189377	CXCL17	0	1	21	NEUTRAL
ENSG00000167861	HID1	1	2	19	NEUTRAL
ENSG00000166670	MMP10	0	5	17	NEUTRAL
ENSG00000104093	DMXL2	0	3	19	NEUTRAL
ENSG00000176635	HORMAD2	0	6	16	NEUTRAL
ENSG00000224389	C4B	0	0	22	NEUTRAL
ENSG00000166920	C15orf48	0	3	19	NEUTRAL
ENSG00000213967	ZNF726	0	1	21	NEUTRAL
ENSG00000122641	INHBA	4	1	17	NEUTRAL
ENSG00000163704	PRRT3	0	0	22	NEUTRAL
ENSG00000169994	MYO7B	0	0	22	NEUTRAL
ENSG00000124939	SCGB2A1	1	2	19	NEUTRAL
ENSG00000144339	TMEFF2	0	1	21	NEUTRAL
ENSG00000100003	SEC14L2	0	6	16	NEUTRAL
ENSG00000078098	FAP	0	1	21	NEUTRAL
ENSG00000170743	SYT9	1	2	19	NEUTRAL
ENSG00000124479	NDP	2	2	18	NEUTRAL
ENSG00000162460	TMEM82	1	3	18	NEUTRAL
ENSG00000188820	FAM26F	0	6	16	NEUTRAL
ENSG00000135679	MDM2	0	0	22	NEUTRAL
ENSG00000115896	PLCL1	0	0	22	NEUTRAL
ENSG00000129170	CSRP3	2	2	18	NEUTRAL
ENSG00000108984	MAP2K6	1	1	20	NEUTRAL
ENSG00000101204	CHRNA4	3	0	19	NEUTRAL
ENSG00000160683	CXCR5	0	7	15	NEUTRAL
ENSG00000163249	CCNYL1	0	0	22	NEUTRAL
ENSG00000267970	AC004899.1	4	1	17	NEUTRAL
ENSG00000118523	CTGF	0	5	17	NEUTRAL
ENSG00000237649	KIFC1	0	0	22	NEUTRAL
ENSG00000029534	ANK1	7	3	12	NEUTRAL
ENSG00000134259	NGF	1	2	19	NEUTRAL
ENSG00000104814	MAP4K1	0	1	21	NEUTRAL
ENSG00000152467	ZSCAN1	0	0	22	NEUTRAL
ENSG00000145244	CORIN	0	0	22	NEUTRAL
ENSG00000149968	MMP3	0	5	17	NEUTRAL
ENSG00000125398	SOX9	1	1	20	NEUTRAL
ENSG00000187010	RHD	1	4	17	NEUTRAL
ENSG00000115233	PSMD14	0	1	21	NEUTRAL

Ensembl	Gene	Nb patients AMP	Nb patients DEL	Nb patients NEUTRAL	Majority
ENSG00000165434	PGM2L1	2	1	19	NEUTRAL
ENSG00000188869	TMC3	0	2	20	NEUTRAL
ENSG00000186340	THBS2	0	4	18	NEUTRAL
ENSG00000269047	AC009041.2	12	0	10	AMP
ENSG00000165264	NDUFB6	0	0	22	NEUTRAL
ENSG00000086062	B4GALT1	0	0	22	NEUTRAL
ENSG00000113262	GRM6	0	1	21	NEUTRAL
ENSG00000206560	ANKRD28	0	0	22	NEUTRAL
ENSG00000135119	RNFT2	0	0	22	NEUTRAL
ENSG00000183682	BMP8A	0	3	19	NEUTRAL
ENSG00000151012	SLC7A11	0	1	21	NEUTRAL
ENSG00000135773	CAPN9	15	0	7	AMP
ENSG00000137674	MMP20	0	5	17	NEUTRAL
ENSG00000088926	F11	0	1	21	NEUTRAL
ENSG00000110375	UPK2	0	7	15	NEUTRAL
ENSG00000213186	TRIM59	1	2	19	NEUTRAL
ENSG00000134970	TMED7	0	2	20	NEUTRAL
ENSG00000018189	RUFY3	0	0	22	NEUTRAL
ENSG00000251201	TMED7-TICAM2	0	2	20	NEUTRAL
ENSG00000139865	TTC6	0	0	22	NEUTRAL
ENSG00000184224	C11orf72	1	1	20	NEUTRAL
ENSG00000100330	MTMR3	0	6	16	NEUTRAL
ENSG00000132326	PER2	0	1	21	NEUTRAL
ENSG00000129654	FOXJ1	1	2	19	NEUTRAL
ENSG00000162595	DIRAS3	0	1	21	NEUTRAL
ENSG00000142513	ACPT	1	1	20	NEUTRAL
ENSG00000137449	CPEB2	0	0	22	NEUTRAL
ENSG00000124935	SCGB1D2	1	2	19	NEUTRAL
ENSG00000114113	RBP2	1	1	20	NEUTRAL
ENSG00000143476	DTL	15	0	7	AMP
ENSG00000179476	C14orf28	0	1	21	NEUTRAL
ENSG00000175206	NPPA	1	3	18	NEUTRAL
ENSG00000116754	SRSF11	0	1	21	NEUTRAL
ENSG00000170965	PLAC1	2	2	18	NEUTRAL
ENSG00000141434	MEP1B	2	1	19	NEUTRAL
ENSG00000165591	FAAH2	2	2	18	NEUTRAL
ENSG00000101883	RHOXF1	2	2	18	NEUTRAL
ENSG00000104299	INTS9	4	4	14	NEUTRAL
ENSG00000172575	RASGRP1	0	3	19	NEUTRAL
ENSG00000175264	CHST1	2	1	19	NEUTRAL
ENSG00000197915	HRNR	15	0	7	AMP
ENSG00000205669	ACOT6	0	1	21	NEUTRAL
ENSG00000117091	CD48	14	0	8	AMP
ENSG00000007001	UPP2	0	1	21	NEUTRAL
ENSG00000171720	HDAC3	0	2	20	NEUTRAL
ENSG00000160469	BRSK1	2	0	20	NEUTRAL
ENSG00000205476	CCDC85C	0	1	21	NEUTRAL
ENSG00000004838	ZMYND10	0	0	22	NEUTRAL
ENSG00000019505	SYT13	2	1	19	NEUTRAL
ENSG00000188001	TPRG1	1	1	20	NEUTRAL
ENSG00000129682	FGF13	2	2	18	NEUTRAL
ENSG00000074590	NUAK1	0	0	22	NEUTRAL
ENSG00000131504	DIAPH1	0	2	20	NEUTRAL
ENSG00000123191	ATP7B	0	4	18	NEUTRAL
ENSG00000183273	CCDC60	0	0	22	NEUTRAL
ENSG00000123388	HOXC11	0	0	22	NEUTRAL
ENSG00000257950	P2RX5-TAX1BP3	0	5	17	NEUTRAL

Ensembl	Gene	Nb patients AMP	Nb patients DEL	Nb patients NEUTRAL	Majority
ENSG00000162849	KIF26B	14	0	8	AMP
ENSG00000082014	SMARCD3	4	0	18	NEUTRAL
ENSG00000188315	C3orf62	0	0	22	NEUTRAL
ENSG00000146950	SHROOM2	2	2	18	NEUTRAL
ENSG00000180182	MED14	2	2	18	NEUTRAL
ENSG00000197372	ZNF675	0	1	21	NEUTRAL
ENSG00000136770	DNAJC1	0	1	21	NEUTRAL
ENSG00000122783	C7orf49	3	1	18	NEUTRAL
ENSG00000126603	GLIS2	13	0	9	AMP
ENSG00000151789	ZNF385D	0	0	22	NEUTRAL
ENSG00000164023	SGMS2	0	1	21	NEUTRAL
ENSG00000104267	CA2	9	0	13	NEUTRAL
ENSG00000013293	SLC7A14	1	1	20	NEUTRAL
ENSG00000170522	ELOVL6	0	1	21	NEUTRAL
ENSG00000129173	E2F8	2	2	18	NEUTRAL
ENSG00000166268	MYRFL	0	0	22	NEUTRAL
ENSG00000124243	BCAS4	3	0	19	NEUTRAL
ENSG00000067715	SYT1	0	0	22	NEUTRAL
ENSG00000122691	TWIST1	3	1	18	NEUTRAL
ENSG00000124467	PSG8	0	1	21	NEUTRAL
ENSG00000168743	NPNT	0	1	21	NEUTRAL
ENSG00000165046	LETM2	9	1	12	NEUTRAL
ENSG00000145491	ROPN1L	2	0	20	NEUTRAL
ENSG00000125869	LAMP5	4	0	18	NEUTRAL
ENSG00000128815	WDFY4	0	1	21	NEUTRAL
ENSG00000138061	CYP1B1	0	0	22	NEUTRAL
ENSG00000186111	PIP5K1C	1	1	20	NEUTRAL
ENSG00000135269	TES	3	1	18	NEUTRAL
ENSG00000185306	C12orf56	0	0	22	NEUTRAL
ENSG00000257727	CNPY2	0	0	22	NEUTRAL
ENSG00000186710	CCDC42B	0	0	22	NEUTRAL
ENSG00000105011	ASF1B	0	2	20	NEUTRAL
ENSG00000107282	APBA1	0	1	21	NEUTRAL
ENSG00000104885	DOT1L	0	2	20	NEUTRAL
ENSG00000143520	FLG2	15	0	7	AMP
ENSG00000170442	KRT86	0	0	22	NEUTRAL
ENSG00000138622	HCN4	0	2	20	NEUTRAL
ENSG00000175832	ETV4	1	4	17	NEUTRAL
ENSG00000129116	PALLD	0	1	21	NEUTRAL
ENSG00000145888	GLRA1	0	2	20	NEUTRAL
ENSG00000064270	ATP2C2	1	12	9	DEL
ENSG00000243414	TICAM2	0	2	20	NEUTRAL
ENSG00000167536	DHRS13	1	2	19	NEUTRAL
ENSG00000163689	C3orf67	0	0	22	NEUTRAL
ENSG00000095585	BLNK	1	1	20	NEUTRAL
ENSG00000164520	RAET1E	0	3	19	NEUTRAL
ENSG00000112799	LY86	0	1	21	NEUTRAL
ENSG00000183196	CHST6	1	13	8	DEL
ENSG00000092853	CLSPN	1	3	18	NEUTRAL
ENSG00000129810	SGOL1	0	0	22	NEUTRAL
ENSG00000135525	MAP7	0	5	17	NEUTRAL
ENSG00000187242	KRT12	1	3	18	NEUTRAL
ENSG00000255346	NOX5	0	3	19	NEUTRAL
ENSG00000064787	BCAS1	3	0	19	NEUTRAL
ENSG00000196569	LAMA2	0	5	17	NEUTRAL
ENSG00000178127	NDUFV2	1	2	19	NEUTRAL
ENSG00000111110	PPM1H	0	0	22	NEUTRAL

Ensembl	Gene	Nb patients AMP	Nb patients DEL	Nb patients NEUTRAL	Majority
ENSG00000215114	UBXN2B	8	0	14	NEUTRAL
ENSG00000078114	NEBL	0	1	21	NEUTRAL
ENSG00000167880	EVPL	1	2	19	NEUTRAL
ENSG00000267819	AC092850.1	0	0	22	NEUTRAL
ENSG00000198780	FAM169A	0	1	21	NEUTRAL
ENSG00000132432	SEC61G	5	0	17	NEUTRAL
ENSG00000176658	MYO1D	1	3	18	NEUTRAL
ENSG00000198901	PRC1	0	2	20	NEUTRAL
ENSG00000156687	UNC5D	8	1	13	NEUTRAL
ENSG00000173917	HOXB2	3	2	17	NEUTRAL
ENSG00000197279	ZNF165	0	0	22	NEUTRAL
ENSG00000196588	MKL1	0	6	16	NEUTRAL
ENSG00000006453	BAIAP2L1	4	0	18	NEUTRAL
ENSG00000181418	DDN	0	0	22	NEUTRAL
ENSG00000143858	SYT2	15	0	7	AMP
ENSG00000079385	CEACAM1	0	1	21	NEUTRAL
ENSG00000169758	C15orf27	0	3	19	NEUTRAL
ENSG00000214114	MYCBP	0	3	19	NEUTRAL
ENSG00000166923	GREM1	0	2	20	NEUTRAL
ENSG00000100721	TCL1A	0	1	21	NEUTRAL
ENSG00000145715	RASA1	0	2	20	NEUTRAL
ENSG00000154767	XPC	0	0	22	NEUTRAL
ENSG00000128923	FAM63B	0	3	19	NEUTRAL
ENSG00000116147	TNR	14	0	8	AMP
ENSG00000102466	FGF14	0	3	19	NEUTRAL
ENSG00000215277	C14orf164	0	0	22	NEUTRAL
ENSG00000198019	FCGR1B	0	0	22	NEUTRAL
ENSG00000137266	SLC22A23	0	1	21	NEUTRAL
ENSG00000123364	HOXC13	0	0	22	NEUTRAL
ENSG00000120093	HOXB3	3	2	17	NEUTRAL
ENSG00000143631	FLG	15	0	7	AMP
ENSG00000173473	SMARCC1	0	0	22	NEUTRAL
ENSG00000179344	HLA-DQB1	0	0	22	NEUTRAL
ENSG00000175894	TSPEAR	2	0	20	NEUTRAL
ENSG00000102606	ARHGEF7	0	3	19	NEUTRAL
ENSG00000154102	C16orf74	1	12	9	DEL
ENSG00000167595	C19orf55	0	1	21	NEUTRAL
ENSG00000072858	SIDT1	2	1	19	NEUTRAL
ENSG00000009724	MASP2	1	3	18	NEUTRAL
ENSG00000219545	RPA3-AS1	3	1	18	NEUTRAL
ENSG00000119715	ESRRB	0	1	21	NEUTRAL
ENSG00000133275	CSNK1G2	0	2	20	NEUTRAL
ENSG00000144744	UBA3	0	0	22	NEUTRAL
ENSG00000117971	CHRNB4	0	2	20	NEUTRAL
ENSG00000198758	EPS8L3	0	2	20	NEUTRAL
ENSG00000146809	ASB15	4	1	17	NEUTRAL
ENSG00000109062	SLC9A3R1	1	1	20	NEUTRAL
ENSG00000125414	MYH2	0	5	17	NEUTRAL
ENSG00000012963	UBR7	0	1	21	NEUTRAL
ENSG00000205517	RGL3	1	1	20	NEUTRAL
ENSG00000144747	TMF1	0	0	22	NEUTRAL
ENSG00000132570	PCBD2	0	2	20	NEUTRAL
ENSG00000149582	TMEM25	0	7	15	NEUTRAL
ENSG00000157335	CLEC18C	1	13	8	DEL
ENSG00000143845	ETNK2	16	0	6	AMP
ENSG00000169836	TACR3	0	1	21	NEUTRAL
ENSG00000164171	ITGA2	0	0	22	NEUTRAL

Ensembl	Gene	Nb patients AMP	Nb patients DEL	Nb patients NEUTRAL	Majority
ENSG00000147655	RSPO2	9	0	13	NEUTRAL
ENSG00000154040	CABYR	1	2	19	NEUTRAL
ENSG00000157212	PAXIP1	4	0	18	NEUTRAL
ENSG00000158315	RHBDL2	0	3	19	NEUTRAL
ENSG00000085274	MYNN	1	1	20	NEUTRAL
ENSG00000198826	ARHGAP11A	0	2	20	NEUTRAL
ENSG00000150667	FSIP1	0	3	19	NEUTRAL
ENSG00000187951	ARHGAP11B	0	2	20	NEUTRAL
ENSG00000095539	SEMA4G	0	1	21	NEUTRAL
ENSG00000213047	DENND1B	14	0	8	AMP
ENSG00000185669	SNAI3	1	12	9	DEL
ENSG00000108100	CCNY	0	0	22	NEUTRAL
ENSG00000008517	IL32	13	0	9	AMP
ENSG00000186409	CCDC30	0	2	20	NEUTRAL
ENSG00000236699	ARHGEF38	0	1	21	NEUTRAL
ENSG00000170921	TANC2	2	1	19	NEUTRAL
ENSG00000188672	RHCE	1	4	17	NEUTRAL
ENSG00000101577	LPIN2	1	2	19	NEUTRAL
ENSG00000179133	C10orf67	0	1	21	NEUTRAL
ENSG00000104147	OIP5	0	3	19	NEUTRAL
ENSG00000162814	SPATA17	15	0	7	AMP
ENSG00000162062	C16orf59	12	0	10	AMP
ENSG00000151611	MMAA	0	1	21	NEUTRAL
ENSG00000137648	TMPRSS4	0	7	15	NEUTRAL
ENSG00000176155	CCDC57	1	2	19	NEUTRAL
ENSG00000187391	MAGI2	3	0	19	NEUTRAL
ENSG00000110660	SLC35F2	0	5	17	NEUTRAL
ENSG00000214491	SEC14L6	0	6	16	NEUTRAL
ENSG00000196812	ZSCAN16	0	0	22	NEUTRAL
ENSG00000133020	MYH8	0	5	17	NEUTRAL
ENSG00000167580	AQP2	0	0	22	NEUTRAL
ENSG00000140650	PMM2	13	0	9	AMP
ENSG00000132122	SPATA6	0	2	20	NEUTRAL
ENSG00000186638	KIF24	0	0	22	NEUTRAL
ENSG00000164109	MAD2L1	0	1	21	NEUTRAL
ENSG00000180902	D2HGDH	0	1	21	NEUTRAL
ENSG00000152382	TADA1	14	0	8	AMP
ENSG00000152763	WDR78	0	1	21	NEUTRAL
ENSG00000185090	MANEAL	0	3	19	NEUTRAL
ENSG00000204060	FOXO6	0	3	19	NEUTRAL
ENSG00000198822	GRM3	3	0	19	NEUTRAL
ENSG00000206530	WDR52	2	1	19	NEUTRAL
ENSG00000196335	STK31	4	1	17	NEUTRAL
ENSG00000153086	ACMSD	1	1	20	NEUTRAL
ENSG00000128683	GAD1	0	1	21	NEUTRAL
ENSG00000105854	PON2	5	0	17	NEUTRAL
ENSG00000118707	TGIF2	3	0	19	NEUTRAL
ENSG00000178896	EXOSC4	8	1	13	NEUTRAL
ENSG00000154305	MIA3	15	0	7	AMP
ENSG00000109814	UGDH	0	0	22	NEUTRAL
ENSG00000119699	TGFB3	0	1	21	NEUTRAL
ENSG00000052344	PRSS8	12	0	10	AMP
ENSG00000183248	CTD-3193O13.9	1	1	20	NEUTRAL
ENSG00000268628	AL121761.2	4	0	18	NEUTRAL
ENSG00000134851	TMEM165	0	0	22	NEUTRAL
ENSG00000159216	RUNX1	2	0	20	NEUTRAL
ENSG00000156097	GPR61	0	2	20	NEUTRAL

Ensembl	Gene	Nb patients AMP	Nb patients DEL	Nb patients NEUTRAL	Majority
ENSG00000113494	PRLR	2	0	20	NEUTRAL
ENSG00000068489	PRR11	2	1	19	NEUTRAL
ENSG00000196090	PTPRT	3	0	19	NEUTRAL
ENSG00000249034	AC005609.1	0	2	20	NEUTRAL
ENSG00000133477	FAM83F	0	6	16	NEUTRAL
ENSG00000138658	C4orf21	0	1	21	NEUTRAL
ENSG00000111266	DUSP16	0	1	21	NEUTRAL
ENSG00000008853	RHOBTB2	4	7	11	NEUTRAL
ENSG00000139656	SMIM2	0	4	18	NEUTRAL
ENSG00000070731	ST6GALNAC2	1	2	19	NEUTRAL
ENSG00000136828	RALGPS1	0	0	22	NEUTRAL
ENSG00000005844	ITGAL	12	0	10	AMP
ENSG00000172748	ZNF596	0	0	22	NEUTRAL
ENSG00000162456	KNCN	0	2	20	NEUTRAL
ENSG00000135100	HNF1A	0	0	22	NEUTRAL
ENSG00000149571	KIRREL3	0	7	15	NEUTRAL
ENSG00000184999	SLC22A10	1	4	17	NEUTRAL
ENSG00000162571	TTLL10	1	2	19	NEUTRAL
ENSG00000164512	ANKRD55	0	0	22	NEUTRAL
ENSG00000105519	CAPS	1	1	20	NEUTRAL
ENSG00000148488	ST8SIA6	0	1	21	NEUTRAL
ENSG00000184743	ATL3	1	4	17	NEUTRAL
ENSG00000164176	EDIL3	0	1	21	NEUTRAL
ENSG00000258740	RP11-293M10.1	0	1	21	NEUTRAL
ENSG00000104894	CD37	1	1	20	NEUTRAL
ENSG00000150556	LYPD6B	0	0	22	NEUTRAL
ENSG00000070501	POLB	7	3	12	NEUTRAL
ENSG00000204616	TRIM31	0	0	22	NEUTRAL
ENSG00000164440	TXLNB	0	6	16	NEUTRAL
ENSG00000164318	EGFLAM	2	0	20	NEUTRAL
ENSG00000116062	MSH6	0	0	22	NEUTRAL
ENSG00000051180	RAD51	0	3	19	NEUTRAL
ENSG00000198018	ENTPD7	0	1	21	NEUTRAL
ENSG00000147419	CCDC25	4	5	13	NEUTRAL
ENSG00000128739	SNRPN	1	0	21	NEUTRAL
ENSG00000074803	SLC12A1	0	3	19	NEUTRAL
ENSG00000105707	HPN	0	1	21	NEUTRAL
ENSG00000172000	ZNF556	0	2	20	NEUTRAL
ENSG00000121335	PRB2	0	1	21	NEUTRAL
ENSG00000180806	HOXC9	0	0	22	NEUTRAL
ENSG00000011485	PPP5C	0	1	21	NEUTRAL
ENSG00000160813	PPP1R35	4	0	18	NEUTRAL
ENSG00000006625	GGCT	4	1	17	NEUTRAL
ENSG00000197050	ZNF420	0	1	21	NEUTRAL
ENSG00000204291	COL15A1	0	1	21	NEUTRAL
ENSG00000256660	CLEC12B	0	1	21	NEUTRAL
ENSG00000173068	BNC2	0	0	22	NEUTRAL
ENSG00000143183	TMCO1	14	0	8	AMP
ENSG00000109180	OCIAD1	0	0	22	NEUTRAL
ENSG00000173578	XCR1	0	0	22	NEUTRAL
ENSG00000180818	HOXC10	0	0	22	NEUTRAL
ENSG00000103852	TTC23	0	0	22	NEUTRAL
ENSG00000180263	FGD6	0	0	22	NEUTRAL
ENSG00000136261	BZW2	3	1	18	NEUTRAL
ENSG00000160191	PDE9A	2	0	20	NEUTRAL
ENSG00000120256	LRP11	0	3	19	NEUTRAL
ENSG00000185551	NR2F2	0	0	22	NEUTRAL

Ensembl	Gene	Nb patients AMP	Nb patients DEL	Nb patients NEUTRAL	Majority
ENSG00000175809	ZNF645	2	2	18	NEUTRAL
ENSG00000111642	CHD4	0	1	21	NEUTRAL
ENSG00000224383	C17orf72	2	1	19	NEUTRAL
ENSG00000188343	FAM92A1	9	0	13	NEUTRAL
ENSG00000161798	AQP5	0	0	22	NEUTRAL
ENSG00000213809	KLRK1	0	1	21	NEUTRAL
ENSG00000177425	PAWR	0	0	22	NEUTRAL
ENSG00000175575	PAAF1	4	1	17	NEUTRAL
ENSG00000153233	PTPRR	0	0	22	NEUTRAL
ENSG00000164105	SAP30	0	1	21	NEUTRAL
ENSG00000165119	HNRNPK	0	1	21	NEUTRAL
ENSG00000258231	RP11-362K2.2	0	0	22	NEUTRAL
ENSG00000165244	ZNF367	0	1	21	NEUTRAL
ENSG00000183850	ZNF730	0	1	21	NEUTRAL
ENSG00000054392	HHAT	15	0	7	AMP
ENSG00000157778	PSMG3	3	1	18	NEUTRAL
ENSG00000051341	POLQ	1	2	19	NEUTRAL
ENSG00000158055	GRHL3	1	4	17	NEUTRAL
ENSG00000081923	ATP8B1	1	1	20	NEUTRAL
ENSG00000115762	PLEKHB2	1	0	21	NEUTRAL
ENSG00000171729	TMEM51	1	3	18	NEUTRAL
ENSG00000159409	CELF3	15	0	7	AMP
ENSG00000163638	ADAMTS9	0	0	22	NEUTRAL
ENSG00000154889	MPPE1	1	2	19	NEUTRAL
ENSG00000197757	HOXC6	0	0	22	NEUTRAL
ENSG00000151694	ADAM17	0	0	22	NEUTRAL
ENSG00000140479	PCSK6	0	0	22	NEUTRAL
ENSG00000175305	CCNE2	9	0	13	NEUTRAL
ENSG00000156030	ELMSAN1	0	1	21	NEUTRAL
ENSG00000179761	PIPOX	1	2	19	NEUTRAL
ENSG00000189108	IL1RAPL2	2	2	18	NEUTRAL
ENSG00000196507	TCEAL3	2	2	18	NEUTRAL
ENSG00000196684	HSH2D	1	1	20	NEUTRAL
ENSG00000127585	FBXL16	12	0	10	AMP
ENSG00000102401	ARMCX3	2	2	18	NEUTRAL
ENSG00000135205	CCDC146	2	0	20	NEUTRAL
ENSG00000079974	RABL2B	0	0	22	NEUTRAL
ENSG00000089012	SIRPG	4	0	18	NEUTRAL
ENSG00000115556	PLCD4	0	0	22	NEUTRAL
ENSG00000114279	FGF12	1	1	20	NEUTRAL
ENSG00000113088	GZMK	0	0	22	NEUTRAL
ENSG00000128908	INO80	0	3	19	NEUTRAL
ENSG00000115266	APC2	0	2	20	NEUTRAL
ENSG00000134215	VAV3	0	2	20	NEUTRAL
ENSG00000179588	ZFPM1	1	12	9	DEL
ENSG00000056736	IL17RB	0	0	22	NEUTRAL
ENSG00000116171	SCP2	0	2	20	NEUTRAL
ENSG00000138771	SHROOM3	1	0	21	NEUTRAL
ENSG00000140443	IGF1R	0	0	22	NEUTRAL
ENSG00000066032	CTNNA2	0	0	22	NEUTRAL
ENSG00000111206	FOXM1	0	1	21	NEUTRAL
ENSG00000088756	ARHGAP28	1	2	19	NEUTRAL
ENSG00000174628	IQCK	12	0	10	AMP
ENSG00000079691	LRRC16A	0	0	22	NEUTRAL
ENSG00000164032	H2AFZ	0	1	21	NEUTRAL
ENSG00000102471	NDFIP2	0	3	19	NEUTRAL
ENSG00000064652	SNX24	0	2	20	NEUTRAL

Ensembl	Gene	Nb patients AMP	Nb patients DEL	Nb patients NEUTRAL	Majority
ENSG00000140548	ZNF710	0	2	20	NEUTRAL
ENSG00000267918	AL117190.2	0	1	21	NEUTRAL
ENSG00000258947	TUBB3	1	12	9	DEL
ENSG00000175279	APITD1	1	3	18	NEUTRAL
ENSG00000087095	NLK	1	2	19	NEUTRAL
ENSG00000170382	LRRN2	16	0	6	AMP
ENSG00000100644	HIF1A	0	1	21	NEUTRAL
ENSG00000215910	C1orf167	1	3	18	NEUTRAL
ENSG00000153707	PTPRD	0	0	22	NEUTRAL
ENSG00000093072	CECR1	0	6	16	NEUTRAL
ENSG00000153904	DDAH1	1	1	20	NEUTRAL
ENSG00000114520	SNX4	1	2	19	NEUTRAL
ENSG00000139438	FAM222A	0	0	22	NEUTRAL
ENSG00000268891	AC006014.1	3	0	19	NEUTRAL
ENSG00000139687	RB1	0	4	18	NEUTRAL
ENSG00000128833	MYO5C	0	3	19	NEUTRAL
ENSG00000106018	VIPR2	4	0	18	NEUTRAL
ENSG00000108395	TRIM37	2	1	19	NEUTRAL
ENSG00000139835	GRTP1	0	3	19	NEUTRAL
ENSG00000118492	ADGB	0	3	19	NEUTRAL
ENSG00000166734	CASC4	0	3	19	NEUTRAL
ENSG00000134202	GSTM3	0	2	20	NEUTRAL
ENSG00000174989	FBXW8	0	0	22	NEUTRAL
ENSG00000139572	GPR84	0	0	22	NEUTRAL
ENSG00000164663	USP49	0	1	21	NEUTRAL
ENSG00000143748	NVL	15	0	7	AMP
ENSG00000268080	AC016885.1	9	0	13	NEUTRAL
ENSG00000005108	THSD7A	3	1	18	NEUTRAL
ENSG00000161996	WDR90	12	0	10	AMP
ENSG00000163749	CCDC158	1	0	21	NEUTRAL
ENSG00000121904	CSMD2	1	4	17	NEUTRAL
ENSG00000141013	GAS8	1	9	12	NEUTRAL
ENSG00000203747	FCGR3A	14	0	8	AMP
ENSG00000167112	TRUB2	0	0	22	NEUTRAL
ENSG00000137513	NARS2	4	2	16	NEUTRAL
ENSG00000082497	SERTAD4	15	0	7	AMP
ENSG00000100985	MMP9	3	0	19	NEUTRAL
ENSG00000137877	SPTBN5	0	3	19	NEUTRAL
ENSG00000110328	GALNT18	2	2	18	NEUTRAL
ENSG00000196247	ZNF107	3	1	18	NEUTRAL
ENSG00000144791	LIMD1	0	0	22	NEUTRAL
ENSG00000174996	KLC2	3	3	16	NEUTRAL
ENSG00000170370	EMX2	0	0	22	NEUTRAL
ENSG00000167653	PSCA	8	1	13	NEUTRAL
ENSG00000169891	REPS2	2	2	18	NEUTRAL
ENSG00000103599	IQCH	0	3	19	NEUTRAL
ENSG00000102921	N4BP1	1	11	10	DEL
ENSG00000185261	KIAA0825	0	2	20	NEUTRAL
ENSG00000213214	ARHGEF35	4	1	17	NEUTRAL
ENSG00000260001	TGFBR3L	1	1	20	NEUTRAL
ENSG00000150687	PRSS23	1	4	17	NEUTRAL
ENSG00000269709	AL109659.1	0	2	20	NEUTRAL
ENSG00000127377	CRYGN	4	0	18	NEUTRAL
ENSG00000162894	FAIM3	15	0	7	AMP
ENSG00000144285	SCN1A	0	1	21	NEUTRAL
ENSG00000100362	PVALB	0	6	16	NEUTRAL
ENSG00000175792	RUVBL1	1	2	19	NEUTRAL

Ensembl	Gene	Nb patients AMP	Nb patients DEL	Nb patients NEUTRAL	Majority
ENSG00000148331	ASB6	0	0	22	NEUTRAL
ENSG00000254598	CSNK2A3	2	2	18	NEUTRAL
ENSG00000163866	SMIM12	1	4	17	NEUTRAL
ENSG00000139154	AEBP2	0	1	21	NEUTRAL
ENSG00000187720	THSD4	0	2	20	NEUTRAL
ENSG00000012048	BRCA1	1	4	17	NEUTRAL
ENSG00000168288	MMADHC	0	0	22	NEUTRAL
ENSG00000171954	CYP4F22	1	1	20	NEUTRAL
ENSG00000111269	CREBL2	0	1	21	NEUTRAL
ENSG00000177885	GRB2	1	2	19	NEUTRAL
ENSG00000158941	CCAR2	4	7	11	NEUTRAL
ENSG00000142945	KIF2C	0	2	20	NEUTRAL
ENSG00000258472	RP11-192H23.4	1	2	19	NEUTRAL
ENSG00000126814	TRMT5	0	1	21	NEUTRAL
ENSG00000187186	RP11-195F19.5	0	0	22	NEUTRAL
ENSG00000172425	TTC36	0	7	15	NEUTRAL
ENSG00000117724	CENPF	15	0	7	AMP
ENSG00000152601	MBNL1	1	1	20	NEUTRAL
ENSG00000181274	FRAT2	0	1	21	NEUTRAL
ENSG00000139624	CERS5	0	0	22	NEUTRAL
ENSG00000070019	GUCY2C	0	1	21	NEUTRAL
ENSG00000055163	CYFIP2	0	2	20	NEUTRAL
ENSG00000147687	TATDN1	9	0	13	NEUTRAL
ENSG00000138641	HERC3	0	1	21	NEUTRAL
ENSG00000136379	ABHD17C	0	2	20	NEUTRAL
ENSG00000197020	ZNF100	0	1	21	NEUTRAL
ENSG00000139725	RHOF	0	0	22	NEUTRAL
ENSG00000175426	PCSK1	0	2	20	NEUTRAL
ENSG00000004478	FKBP4	0	1	21	NEUTRAL
ENSG00000154611	PSMA8	1	1	20	NEUTRAL
ENSG00000257446	ZNF878	0	2	20	NEUTRAL
ENSG00000153936	HS2ST1	0	1	21	NEUTRAL
ENSG00000187764	SEMA4D	0	1	21	NEUTRAL
ENSG00000269343	ZNF587B	0	0	22	NEUTRAL
ENSG00000012779	ALOX5	0	0	22	NEUTRAL
ENSG00000167716	WDR81	0	5	17	NEUTRAL
ENSG00000162994	CLHC1	0	0	22	NEUTRAL
ENSG00000177291	GJD4	0	1	21	NEUTRAL
ENSG00000108829	LRRC59	3	2	17	NEUTRAL
ENSG00000039123	SKIV2L2	0	0	22	NEUTRAL
ENSG00000170776	AKAP13	0	2	20	NEUTRAL
ENSG00000054967	RELT	3	1	18	NEUTRAL
ENSG00000143228	NUF2	15	0	7	AMP
ENSG00000139289	PHLDA1	0	0	22	NEUTRAL
ENSG00000157107	FCHO2	0	1	21	NEUTRAL
ENSG00000001630	CYP51A1	3	0	19	NEUTRAL
ENSG00000170379	FAM115C	5	0	17	NEUTRAL
ENSG00000120071	KANSL1	2	2	18	NEUTRAL
ENSG00000078618	NRD1	0	2	20	NEUTRAL
ENSG00000171130	ATP6V0E2	4	0	18	NEUTRAL
ENSG00000104313	EYA1	8	0	14	NEUTRAL
ENSG00000164114	MAP9	0	1	21	NEUTRAL
ENSG00000106479	ZNF862	4	0	18	NEUTRAL
ENSG00000152620	NADK2	2	0	20	NEUTRAL
ENSG00000144452	ABCA12	0	0	22	NEUTRAL
ENSG00000186666	BCDIN3D	0	0	22	NEUTRAL
ENSG00000179673	RPRML	2	2	18	NEUTRAL

Ensembl	Gene	Nb patients AMP	Nb patients DEL	Nb patients NEUTRAL	Majority
ENSG00000163630	SYNPR	0	0	22	NEUTRAL
ENSG00000079335	CDC14A	0	2	20	NEUTRAL
ENSG00000164309	CMYA5	0	1	21	NEUTRAL
ENSG00000163714	U2SURP	1	1	20	NEUTRAL
ENSG00000128039	SRD5A3	0	0	22	NEUTRAL
ENSG00000197594	ENPP1	0	5	17	NEUTRAL
ENSG00000124813	RUNX2	0	0	22	NEUTRAL
ENSG00000103966	EHD4	0	3	19	NEUTRAL
ENSG00000196345	ZKSCAN7	0	0	22	NEUTRAL
ENSG00000134107	BHLHE40	0	1	21	NEUTRAL
ENSG00000258315	C17orf49	0	5	17	NEUTRAL
ENSG00000160957	RECQL4	8	1	13	NEUTRAL
ENSG00000135314	KHDC1	0	4	18	NEUTRAL
ENSG00000165487	MICU2	0	3	19	NEUTRAL
ENSG00000167306	MYO5B	1	1	20	NEUTRAL
ENSG00000143450	OAZ3	15	0	7	AMP
ENSG00000140398	NEIL1	0	3	19	NEUTRAL
ENSG00000162063	CCNF	12	0	10	AMP
ENSG00000168264	IRF2BP2	15	0	7	AMP
ENSG00000244607	CCDC13	0	0	22	NEUTRAL
ENSG00000160714	UBE2Q1	15	0	7	AMP
ENSG00000176371	ZSCAN2	0	2	20	NEUTRAL
ENSG00000071909	MYO3B	0	1	21	NEUTRAL
ENSG00000048052	HDAC9	3	1	18	NEUTRAL
ENSG00000139146	FAM60A	0	0	22	NEUTRAL
ENSG00000204099	NEU4	0	1	21	NEUTRAL
ENSG00000113648	H2AFY	0	2	20	NEUTRAL
ENSG00000088727	KIF9	0	0	22	NEUTRAL
ENSG00000186474	KLK12	1	1	20	NEUTRAL
ENSG00000170638	TRABD	0	6	16	NEUTRAL
ENSG00000133740	E2F5	9	0	13	NEUTRAL
ENSG00000166037	CEP57	1	5	16	NEUTRAL
ENSG00000187109	NAP1L1	0	0	22	NEUTRAL
ENSG00000117983	MUC5B	2	2	18	NEUTRAL

Supplemental File 1 / Table S8 : Varatio-left list of NAT/PC pairs. In this list, PC expression is potentially regulated by their corresponding NAT. The genomic copy number status was indicated for each gene

The read counts varRatio has been calculated as described in the main manuscript. Pairs of NAT/PC genes corresponding to righttmost values of the varRatio distribution have been selected by applying a threshold (mean ± standard deviation) to the log-transformed distribution of the varRatios (S8-Supplemental File 1). Pairs of genes where either the protein-coding or the antisense was expressed in less than 7 tumor samples or 7 healthy samples have been discarded.

Ensembl	Gene	Nb patients AMP	Nb patients DEL	Nb patients NEUTRAL	Majority
ENSG00000062282	DGAT2	3	1	18	NEUTRAL
ENSG00000174720	LARP7	0	1	21	NEUTRAL
ENSG00000170262	MRAP	2	0	20	NEUTRAL
ENSG00000130876	SLC7A10	0	0	22	NEUTRAL
ENSG00000165868	HSPA12A	0	0	22	NEUTRAL
ENSG00000168309	FAM107A	0	0	22	NEUTRAL
ENSG00000079308	TNS1	0	0	22	NEUTRAL
ENSG00000216921	AC131097.4	0	1	21	NEUTRAL
ENSG00000120049	KCNIP2	0	1	21	NEUTRAL
ENSG00000177666	PNPLA2	2	2	18	NEUTRAL
ENSG00000186642	PDE2A	3	1	18	NEUTRAL
ENSG00000164849	GPR146	3	1	18	NEUTRAL
ENSG00000135218	CD36	3	0	19	NEUTRAL
ENSG00000140263	SORD	0	3	19	NEUTRAL
ENSG00000166947	EPB42	0	3	19	NEUTRAL
ENSG00000198947	DMD	2	2	18	NEUTRAL
ENSG00000133800	LYVE1	2	2	18	NEUTRAL
ENSG00000221866	PLXNA4	3	1	18	NEUTRAL
ENSG00000079435	LIPE	0	1	21	NEUTRAL
ENSG00000182118	FAM89A	15	0	7	AMP
ENSG00000128918	ALDH1A2	0	3	19	NEUTRAL
ENSG00000184347	SLIT3	0	2	20	NEUTRAL
ENSG00000105426	PTPRS	1	1	20	NEUTRAL
ENSG00000185761	ADAMTSL5	0	2	20	NEUTRAL
ENSG00000181577	C6orf223	0	0	22	NEUTRAL
ENSG00000073712	FERMT2	1	0	21	NEUTRAL
ENSG00000133392	MYH11	12	0	10	AMP
ENSG00000111859	NEDD9	0	1	21	NEUTRAL
ENSG00000181856	SLC2A4	0	5	17	NEUTRAL
ENSG00000174944	P2RY14	1	1	20	NEUTRAL
ENSG00000144908	ALDH1L1	1	2	19	NEUTRAL
ENSG00000163431	LMOD1	15	0	7	AMP
ENSG00000147883	CDKN2B	0	0	22	NEUTRAL
ENSG00000213494	CCL14	1	3	18	NEUTRAL
ENSG00000132970	WASF3	0	3	19	NEUTRAL
ENSG00000183090	FREM3	0	1	21	NEUTRAL
ENSG00000141576	RNF157	1	2	19	NEUTRAL
ENSG00000198363	ASPH	8	0	14	NEUTRAL
ENSG00000183549	ACSM5	12	0	10	AMP
ENSG00000115504	EHBP1	0	0	22	NEUTRAL
ENSG00000134020	PEBP4	4	7	11	NEUTRAL
ENSG00000152270	PDE3B	2	2	18	NEUTRAL

Ensembl	Gene	Nb patients AMP	Nb patients DEL	Nb patients NEUTRAL	Majority
ENSG00000154258	ABCA9	2	1	19	NEUTRAL
ENSG00000169744	LDB2	0	0	22	NEUTRAL
ENSG00000112769	LAMA4	0	7	15	NEUTRAL
ENSG00000163637	PRICKLE2	0	0	22	NEUTRAL
ENSG00000140199	SLC12A6	0	2	20	NEUTRAL
ENSG00000140945	CDH13	1	12	9	DEL
ENSG00000165072	MAMDC2	0	1	21	NEUTRAL
ENSG00000104490	NCALD	9	0	13	NEUTRAL
ENSG00000179388	EGR3	4	7	11	NEUTRAL
ENSG00000180155	LYNX1	8	1	13	NEUTRAL
ENSG00000182667	NTM	0	7	15	NEUTRAL
ENSG00000147202	DIAPH2	2	2	18	NEUTRAL
ENSG00000120645	IQSEC3	0	0	22	NEUTRAL
ENSG00000119729	RHOQ	0	0	22	NEUTRAL
ENSG00000139304	PTPRQ	0	0	22	NEUTRAL
ENSG00000112964	GHR	1	0	21	NEUTRAL
ENSG00000172927	MYEOV	4	0	18	NEUTRAL
ENSG00000110693	SOX6	2	2	18	NEUTRAL
ENSG00000213759	UGT2B11	0	0	22	NEUTRAL
ENSG00000048740	CELF2	0	1	21	NEUTRAL
ENSG00000182782	HCAR2	0	0	22	NEUTRAL
ENSG00000152592	DMP1	0	1	21	NEUTRAL
ENSG00000133401	PDZD2	2	0	20	NEUTRAL
ENSG00000126218	F10	0	3	19	NEUTRAL
ENSG00000151632	AKR1C2	0	1	21	NEUTRAL
ENSG00000141052	MYOCD	0	5	17	NEUTRAL
ENSG00000162771	FAM71A	15	0	7	AMP
ENSG00000171914	TLN2	0	3	19	NEUTRAL
ENSG00000240654	C1QTNF9	0	3	19	NEUTRAL
ENSG00000109819	PPARGC1A	0	0	22	NEUTRAL
ENSG00000173210	ABLIM3	0	2	20	NEUTRAL
ENSG00000173482	PTPRM	1	2	19	NEUTRAL
ENSG00000254349	RP11-758M4.1	8	0	14	NEUTRAL
ENSG00000182871	COL18A1	2	0	20	NEUTRAL
ENSG00000150907	FOXO1	0	4	18	NEUTRAL
ENSG00000119042	SATB2	0	0	22	NEUTRAL
ENSG00000168594	ADAM29	0	1	21	NEUTRAL
ENSG00000152104	PTPN14	15	0	7	AMP
ENSG00000147257	GPC3	2	2	18	NEUTRAL
ENSG00000138685	FGF2	0	1	21	NEUTRAL
ENSG00000111339	ART4	0	1	21	NEUTRAL
ENSG00000134760	DSG1	2	1	19	NEUTRAL
ENSG00000101367	MAPRE1	3	0	19	NEUTRAL
ENSG00000167701	GPT	8	1	13	NEUTRAL
ENSG00000245848	CEBPA	0	0	22	NEUTRAL
ENSG00000162367	TAL1	0	2	20	NEUTRAL
ENSG00000114771	AADAC	1	1	20	NEUTRAL
ENSG00000146648	EGFR	5	0	17	NEUTRAL
ENSG00000111077	TENC1	0	0	22	NEUTRAL
ENSG00000118971	CCND2	0	1	21	NEUTRAL
ENSG00000101605	MYOM1	1	2	19	NEUTRAL
ENSG00000169554	ZEB2	0	2	20	NEUTRAL

Ensembl	Gene	Nb patients AMP	Nb patients DEL	Nb patients NEUTRAL	Majority
ENSG00000170145	SIK2	1	6	15	NEUTRAL
ENSG00000150471	LPHN3	0	0	22	NEUTRAL
ENSG00000256618	MTRNR2L1	0	0	22	NEUTRAL
ENSG00000172201	ID4	0	0	22	NEUTRAL
ENSG00000103489	XYLT1	12	0	10	AMP
ENSG00000163297	ANTXR2	0	1	21	NEUTRAL
ENSG00000145335	SNCA	0	1	21	NEUTRAL
ENSG00000006652	IFRD1	4	1	17	NEUTRAL
ENSG00000168917	SLC35G2	1	1	20	NEUTRAL
ENSG00000151474	FRMD4A	0	1	21	NEUTRAL
ENSG00000178814	OPLAH	8	1	13	NEUTRAL
ENSG00000157510	AFAP1L1	0	2	20	NEUTRAL
ENSG00000004776	HSPB6	0	1	21	NEUTRAL
ENSG00000145934	TENM2	0	2	20	NEUTRAL
ENSG00000101542	CDH20	1	1	20	NEUTRAL
ENSG00000086570	FAT2	0	2	20	NEUTRAL
ENSG00000166960	CCDC178	2	1	19	NEUTRAL
ENSG00000123119	NECAB1	9	0	13	NEUTRAL
ENSG00000008394	MGST1	0	1	21	NEUTRAL
ENSG00000129151	BBOX1	2	2	18	NEUTRAL
ENSG00000101331	CCM2L	3	0	19	NEUTRAL
ENSG00000112280	COL9A1	0	5	17	NEUTRAL
ENSG00000149256	TENM4	3	2	17	NEUTRAL
ENSG00000134324	LPIN1	0	0	22	NEUTRAL
ENSG00000183117	CSMD1	3	6	13	NEUTRAL
ENSG00000172572	PDE3A	0	1	21	NEUTRAL
ENSG00000169291	SHE	15	0	7	AMP
ENSG00000065534	MYLK	1	2	19	NEUTRAL
ENSG00000125337	KIF25	0	5	17	NEUTRAL
ENSG00000127954	STEAP4	4	0	18	NEUTRAL
ENSG00000146477	SLC22A3	0	4	18	NEUTRAL
ENSG00000153823	PID1	0	1	21	NEUTRAL
ENSG00000182902	SLC25A18	0	6	16	NEUTRAL
ENSG00000071051	NCK2	0	0	22	NEUTRAL
ENSG00000126091	ST3GAL3	0	2	20	NEUTRAL
ENSG00000116678	LEPR	0	1	21	NEUTRAL
ENSG00000171368	TPPP	3	2	17	NEUTRAL
ENSG00000155380	SLC16A1	0	2	20	NEUTRAL
ENSG00000151320	AKAP6	0	0	22	NEUTRAL
ENSG00000094963	FMO2	14	0	8	AMP
ENSG00000160678	S100A1	15	0	7	AMP
ENSG00000225968	ELFN1	3	1	18	NEUTRAL
ENSG00000166265	CYYR1	2	0	20	NEUTRAL
ENSG00000135862	LAMC1	14	0	8	AMP
ENSG00000164318	EGFLAM	2	0	20	NEUTRAL
ENSG00000079215	SLC1A3	2	0	20	NEUTRAL
ENSG00000175161	CADM2	0	0	22	NEUTRAL
ENSG00000064300	NGFR	3	2	17	NEUTRAL
ENSG00000006007	GDE1	12	0	10	AMP
ENSG00000182253	SYNM	0	0	22	NEUTRAL
ENSG00000133424	LARGE	0	6	16	NEUTRAL
ENSG00000169116	PARM1	1	0	21	NEUTRAL

Ensembl	Gene	Nb patients AMP	Nb patients DEL	Nb patients NEUTRAL	Majority
ENSG00000273079	GRIN2B	0	1	21	NEUTRAL
ENSG00000136160	EDNRB	0	4	18	NEUTRAL
ENSG00000177951	BET1L	0	0	22	NEUTRAL
ENSG00000198382	UVRAG	2	1	19	NEUTRAL
ENSG00000165152	TMEM246	0	1	21	NEUTRAL
ENSG00000131378	RFTN1	0	0	22	NEUTRAL
ENSG00000165914	TTC7B	0	1	21	NEUTRAL
ENSG00000130584	ZBTB46	3	0	19	NEUTRAL
ENSG00000118507	AKAP7	0	5	17	NEUTRAL
ENSG00000042062	FAM65C	3	0	19	NEUTRAL
ENSG00000109906	ZBTB16	0	6	16	NEUTRAL
ENSG00000175065	DSG4	2	1	19	NEUTRAL
ENSG00000102802	MEDAG	0	3	19	NEUTRAL
ENSG00000069122	GPR116	1	0	21	NEUTRAL
ENSG00000116141	MARK1	15	0	7	AMP
ENSG00000187527	ATP13A5	1	1	20	NEUTRAL
ENSG00000127743	IL17B	0	2	20	NEUTRAL
ENSG00000111536	IL26	0	0	22	NEUTRAL
ENSG00000175538	KCNE3	2	1	19	NEUTRAL
ENSG00000177697	CD151	2	2	18	NEUTRAL
ENSG00000241258	CRCP	3	1	18	NEUTRAL
ENSG00000166025	AMOTL1	1	5	16	NEUTRAL
ENSG00000142798	HSPG2	1	3	18	NEUTRAL
ENSG00000123411	IKZF4	0	0	22	NEUTRAL
ENSG00000121039	RDH10	8	0	14	NEUTRAL
ENSG00000169432	SCN9A	0	1	21	NEUTRAL
ENSG00000111537	IFNG	0	0	22	NEUTRAL
ENSG00000064205	WISP2	3	0	19	NEUTRAL
ENSG00000100191	SLC5A4	0	6	16	NEUTRAL
ENSG00000167772	ANGPTL4	1	1	20	NEUTRAL
ENSG00000197928	ZNF677	2	0	20	NEUTRAL
ENSG00000101680	LAMA1	1	2	19	NEUTRAL
ENSG00000257327	RP11-650K20.3	0	0	22	NEUTRAL
ENSG00000087903	RFX2	1	1	20	NEUTRAL
ENSG00000106714	CNTNAP3	0	0	22	NEUTRAL
ENSG00000173114	LRRN3	3	1	18	NEUTRAL
ENSG00000196935	SRGAP1	0	0	22	NEUTRAL
ENSG00000242259	C22orf39	0	6	16	NEUTRAL
ENSG00000107104	KANK1	0	0	22	NEUTRAL
ENSG00000160145	KALRN	1	2	19	NEUTRAL
ENSG00000143819	EPHX1	15	0	7	AMP
ENSG00000176595	KBTBD11	3	6	13	NEUTRAL
ENSG00000131408	NR1H2	1	1	20	NEUTRAL
ENSG00000145860	RNF145	0	2	20	NEUTRAL
ENSG00000204176	SYT15	0	2	20	NEUTRAL
ENSG00000049540	ELN	3	0	19	NEUTRAL
ENSG00000016602	CLCA4	0	1	21	NEUTRAL
ENSG00000108924	HLF	2	2	18	NEUTRAL
ENSG00000101187	SLCO4A1	3	0	19	NEUTRAL
ENSG00000244482	LILRA6	2	0	20	NEUTRAL
ENSG00000138640	FAM13A	0	1	21	NEUTRAL
ENSG00000117707	PROX1	15	0	7	AMP

Ensembl	Gene	Nb patients AMP	Nb patients DEL	Nb patients NEUTRAL	Majority
ENSG00000154080	CHST9	1	1	20	NEUTRAL
ENSG00000100242	SUN2	0	6	16	NEUTRAL
ENSG00000123992	DNPEP	0	0	22	NEUTRAL
ENSG00000139220	PPFIA2	0	0	22	NEUTRAL
ENSG00000130957	FBP2	0	1	21	NEUTRAL
ENSG00000196557	CACNA1H	12	0	10	AMP
ENSG00000139641	ESYT1	0	0	22	NEUTRAL
ENSG00000182463	TSHZ2	3	0	19	NEUTRAL
ENSG00000185610	DBX2	0	0	22	NEUTRAL
ENSG00000261611	AC010547.9	1	13	8	DEL
ENSG00000163762	TM4SF18	1	1	20	NEUTRAL
ENSG00000197930	ERO1L	1	0	21	NEUTRAL
ENSG00000100092	SH3BP1	0	6	16	NEUTRAL
ENSG00000058091	CDK14	4	0	18	NEUTRAL
ENSG00000182534	MXRA7	1	2	19	NEUTRAL
ENSG00000105339	DENND3	8	1	13	NEUTRAL
ENSG00000074047	GLI2	0	0	22	NEUTRAL
ENSG00000151364	KCTD14	4	2	16	NEUTRAL
ENSG00000170214	ADRA1B	0	2	20	NEUTRAL
ENSG00000115361	ACADL	0	0	22	NEUTRAL
ENSG00000116539	ASH1L	15	0	7	AMP
ENSG00000164687	FABP5	9	0	13	NEUTRAL
ENSG00000072210	ALDH3A2	0	5	17	NEUTRAL
ENSG00000186453	FAM228A	0	0	22	NEUTRAL
ENSG00000167202	TBC1D2B	0	2	20	NEUTRAL
ENSG00000104805	NUCB1	1	1	20	NEUTRAL
ENSG00000126804	ZBTB1	0	1	21	NEUTRAL
ENSG00000066933	MYO9A	0	2	20	NEUTRAL
ENSG00000164879	CA3	9	0	13	NEUTRAL
ENSG00000035664	DAPK2	0	3	19	NEUTRAL
ENSG00000154655	L3MBTL4	1	2	19	NEUTRAL
ENSG00000151276	MAGI1	0	0	22	NEUTRAL
ENSG00000128652	HOXD3	0	1	21	NEUTRAL
ENSG00000135406	PRPH	0	0	22	NEUTRAL
ENSG00000100504	PYGL	1	0	21	NEUTRAL
ENSG00000166575	TMEM135	0	4	18	NEUTRAL
ENSG00000140873	ADAMTS18	1	12	9	DEL
ENSG00000172159	FRMD3	0	1	21	NEUTRAL
ENSG00000109686	SH3D19	0	1	21	NEUTRAL
ENSG00000177311	ZBTB38	1	1	20	NEUTRAL
ENSG00000099957	P2RX6	0	6	16	NEUTRAL
ENSG00000073910	FRY	0	3	19	NEUTRAL
ENSG00000108309	RUNDC3A	1	3	18	NEUTRAL
ENSG00000072736	NFATC3	1	13	8	DEL
ENSG00000058063	ATP11B	1	1	20	NEUTRAL
ENSG00000101230	ISM1	4	0	18	NEUTRAL
ENSG00000140459	CYP11A1	0	2	20	NEUTRAL
ENSG00000167614	TTYH1	2	0	20	NEUTRAL
ENSG00000105875	WDR91	3	1	18	NEUTRAL
ENSG00000268923	AL645922.1	0	0	22	NEUTRAL
ENSG00000137960	GIPC2	1	1	20	NEUTRAL
ENSG00000131730	CKMT2	0	1	21	NEUTRAL

Ensembl	Gene	Nb patients AMP	Nb patients DEL	Nb patients NEUTRAL	Majority
ENSG00000171587	DSCAM	2	0	20	NEUTRAL
ENSG00000121057	AKAP1	2	2	18	NEUTRAL
ENSG00000101335	MYL9	3	0	19	NEUTRAL
ENSG00000108424	KPNB1	3	2	17	NEUTRAL
ENSG00000060709	RIMBP2	0	0	22	NEUTRAL
ENSG00000258818	RNASE4	0	0	22	NEUTRAL
ENSG00000141404	GNAL	1	2	19	NEUTRAL
ENSG00000142185	TRPM2	2	0	20	NEUTRAL
ENSG00000128655	PDE11A	0	1	21	NEUTRAL
ENSG00000106511	MEOX2	3	1	18	NEUTRAL
ENSG00000110367	DDX6	0	7	15	NEUTRAL
ENSG00000085511	MAP3K4	0	4	18	NEUTRAL
ENSG00000161533	ACOX1	1	2	19	NEUTRAL
ENSG00000113296	THBS4	0	1	21	NEUTRAL
ENSG00000106004	HOXA5	4	1	17	NEUTRAL
ENSG00000151962	RBM46	0	1	21	NEUTRAL
ENSG00000165795	NDRG2	0	0	22	NEUTRAL
ENSG00000145362	ANK2	0	1	21	NEUTRAL
ENSG00000204371	EHMT2	0	0	22	NEUTRAL
ENSG00000143776	CDC42BPA	15	0	7	AMP
ENSG00000091436	MLTK	0	0	22	NEUTRAL
ENSG00000172554	SNTG2	0	0	22	NEUTRAL
ENSG00000134352	IL6ST	0	0	22	NEUTRAL
ENSG00000171408	PDE7B	0	5	17	NEUTRAL
ENSG00000204084	INPP5B	0	3	19	NEUTRAL
ENSG00000214357	NEURL1B	0	2	20	NEUTRAL
ENSG00000058668	ATP2B4	16	0	6	AMP
ENSG00000197182	FLJ27365	0	6	16	NEUTRAL
ENSG00000159658	EFCAB14	0	2	20	NEUTRAL
ENSG00000169594	BNC1	0	2	20	NEUTRAL
ENSG00000079337	RAPGEF3	0	0	22	NEUTRAL
ENSG00000174099	MSRB3	0	0	22	NEUTRAL
ENSG00000135407	AVIL	0	0	22	NEUTRAL
ENSG00000141564	RPTOR	1	2	19	NEUTRAL
ENSG00000119514	GALNT12	0	1	21	NEUTRAL
ENSG00000152591	DSPP	0	1	21	NEUTRAL
ENSG00000181195	PENK	7	1	14	NEUTRAL
ENSG00000140391	TSPAN3	0	3	19	NEUTRAL
ENSG00000026025	VIM	0	1	21	NEUTRAL
ENSG00000132405	TBC1D14	0	0	22	NEUTRAL
ENSG00000198799	LRIG2	0	2	20	NEUTRAL
ENSG00000113578	FGF1	0	2	20	NEUTRAL
ENSG00000126950	TMEM35	2	2	18	NEUTRAL
ENSG00000172548	NIPAL4	0	2	20	NEUTRAL
ENSG00000206579	XKR4	7	0	15	NEUTRAL
ENSG00000106484	MEST	3	1	18	NEUTRAL
ENSG00000127329	PTPRB	0	0	22	NEUTRAL
ENSG00000123091	RNF11	0	2	20	NEUTRAL
ENSG00000184647	PRSS55	3	7	12	NEUTRAL
ENSG00000134508	CABLES1	1	2	19	NEUTRAL
ENSG00000104324	CPQ	9	0	13	NEUTRAL
ENSG00000172270	BSG	0	2	20	NEUTRAL

Ensembl	Gene	Nb patients AMP	Nb patients DEL	Nb patients NEUTRAL	Majority
ENSG00000138031	ADCY3	0	0	22	NEUTRAL
ENSG00000104154	SLC30A4	0	3	19	NEUTRAL
ENSG00000117682	DHDDS	1	4	17	NEUTRAL
ENSG00000113319	RASGRF2	0	1	21	NEUTRAL
ENSG00000147526	TACC1	9	1	12	NEUTRAL
ENSG00000115459	ELMOD3	0	0	22	NEUTRAL
ENSG00000067191	CACNB1	1	3	18	NEUTRAL
ENSG00000198542	ITGBL1	0	3	19	NEUTRAL
ENSG00000166250	CLMP	0	7	15	NEUTRAL
ENSG00000164929	BAALC	9	0	13	NEUTRAL
ENSG00000023287	RB1CC1	7	1	14	NEUTRAL
ENSG00000148671	ADIRF	0	2	20	NEUTRAL
ENSG00000164741	DLC1	3	6	13	NEUTRAL
ENSG00000143382	ADAMTSL4	15	0	7	AMP
ENSG00000113790	EHHADH	1	1	20	NEUTRAL
ENSG00000181092	ADIPOQ	1	1	20	NEUTRAL
ENSG00000167716	WDR81	0	5	17	NEUTRAL
ENSG00000127452	FBXL12	1	1	20	NEUTRAL
ENSG00000256515	CCL3L3	1	3	18	NEUTRAL
ENSG00000168497	SDPR	0	1	21	NEUTRAL
ENSG00000133063	CHIT1	16	0	6	AMP
ENSG00000110917	MLEC	0	0	22	NEUTRAL
ENSG00000070010	UFD1L	0	6	16	NEUTRAL
ENSG00000104765	BNIP3L	4	6	12	NEUTRAL
ENSG00000169282	KCNAB1	1	2	19	NEUTRAL
ENSG00000171840	NINJ2	0	1	21	NEUTRAL
ENSG00000173281	PPP1R3B	3	7	12	NEUTRAL
ENSG00000163171	CDC42EP3	0	0	22	NEUTRAL
ENSG00000205863	C1QTNF9B	0	3	19	NEUTRAL
ENSG00000125733	TRIP10	1	1	20	NEUTRAL
ENSG00000198832	SELM	0	6	16	NEUTRAL
ENSG00000006837	CDKL3	0	2	20	NEUTRAL
ENSG00000132466	ANKRD17	0	0	22	NEUTRAL
ENSG00000103043	VAC14	1	13	8	DEL
ENSG00000141433	ADCYAP1	1	2	19	NEUTRAL
ENSG00000109099	PMP22	0	5	17	NEUTRAL
ENSG00000186567	CEACAM19	0	1	21	NEUTRAL
ENSG00000213949	ITGA1	0	0	22	NEUTRAL
ENSG00000134627	PIWIL4	1	5	16	NEUTRAL
ENSG00000166596	WDR16	0	5	17	NEUTRAL
ENSG00000150722	PPP1R1C	0	1	21	NEUTRAL
ENSG00000168237	GLYCTK	0	0	22	NEUTRAL
ENSG00000159231	CBR3	2	0	20	NEUTRAL
ENSG00000173064	HECTD4	0	0	22	NEUTRAL
ENSG00000154945	ANKRD40	3	2	17	NEUTRAL
ENSG00000266964	FXYD1	0	1	21	NEUTRAL
ENSG00000105270	CLIP3	0	1	21	NEUTRAL
ENSG00000066135	KDM4A	0	2	20	NEUTRAL
ENSG00000108387	SEPT4	2	2	18	NEUTRAL
ENSG00000187140	FOXD3	0	1	21	NEUTRAL
ENSG00000253293	HOXA10	4	1	17	NEUTRAL
ENSG00000069431	ABCC9	0	1	21	NEUTRAL

**Ensembl**	**Gene**	**Nb patients AMP**	**Nb patients DEL**	**Nb patients NEUTRAL**	**Majority**
ENSG00000214827	MTCP1	2	2	18	NEUTRAL
ENSG00000145284	SCD5	0	1	21	NEUTRAL
ENSG00000103657	HERC1	0	3	19	NEUTRAL
ENSG00000144724	PTPRG	0	0	22	NEUTRAL
ENSG00000148180	GSN	0	0	22	NEUTRAL
ENSG00000151422	FER	0	2	20	NEUTRAL
ENSG00000101972	STAG2	2	2	18	NEUTRAL
ENSG00000132874	SLC14A2	2	2	18	NEUTRAL
ENSG00000107957	SH3PXD2A	0	1	21	NEUTRAL
ENSG00000240771	ARHGEF25	0	0	22	NEUTRAL
ENSG00000153956	CACNA2D1	3	0	19	NEUTRAL
ENSG00000134569	LRP4	1	1	20	NEUTRAL
ENSG00000158813	EDA	2	2	18	NEUTRAL
ENSG00000129493	HEATR5A	1	0	21	NEUTRAL
ENSG00000134871	COL4A2	0	3	19	NEUTRAL
ENSG00000171885	AQP4	1	1	20	NEUTRAL
ENSG00000153147	SMARCA5	0	1	21	NEUTRAL
ENSG00000115850	LCT	1	1	20	NEUTRAL
ENSG00000256683	ZNF350	1	0	21	NEUTRAL
ENSG00000250722	SEPP1	1	0	21	NEUTRAL
ENSG00000031081	ARHGAP31	2	1	19	NEUTRAL
ENSG00000197892	KIF13B	4	4	14	NEUTRAL
ENSG00000180875	GREM2	14	0	8	AMP
ENSG00000089159	PXN	0	0	22	NEUTRAL
ENSG00000213672	NCKIPSD	0	0	22	NEUTRAL
ENSG00000120896	SORBS3	4	7	11	NEUTRAL
ENSG00000188107	EYS	0	3	19	NEUTRAL
ENSG00000170653	ATF7	0	0	22	NEUTRAL
ENSG00000094755	GABRP	0	2	20	NEUTRAL
ENSG00000147813	NAPRT1	8	1	13	NEUTRAL
ENSG00000138172	CALHM2	0	1	21	NEUTRAL
ENSG00000127951	FGL2	2	0	20	NEUTRAL
ENSG00000091409	ITGA6	0	0	22	NEUTRAL
ENSG00000066382	MPPED2	3	2	17	NEUTRAL
ENSG00000197142	ACSL5	0	0	22	NEUTRAL
ENSG00000080503	SMARCA2	0	0	22	NEUTRAL
ENSG00000136560	TANK	0	1	21	NEUTRAL
ENSG00000165626	BEND7	0	1	21	NEUTRAL
ENSG00000179820	MYADM	2	0	20	NEUTRAL
ENSG00000088256	GNA11	0	2	20	NEUTRAL
ENSG00000143248	RGS5	15	0	7	AMP
ENSG00000185721	DRG1	0	6	16	NEUTRAL
ENSG00000166532	RIMKLB	0	1	21	NEUTRAL
ENSG00000100024	UPB1	0	6	16	NEUTRAL
ENSG00000151532	VTI1A	0	0	22	NEUTRAL
ENSG00000175634	RPS6KB2	2	1	19	NEUTRAL
ENSG00000073008	PVR	0	1	21	NEUTRAL
ENSG00000105618	PRPF31	2	0	20	NEUTRAL
ENSG00000149564	ESAM	0	7	15	NEUTRAL
ENSG00000101425	BPI	3	0	19	NEUTRAL
ENSG00000111879	FAM184A	0	6	16	NEUTRAL
ENSG00000175664	TEX26	0	3	19	NEUTRAL

Ensembl	Gene	Nb patients AMP	Nb patients DEL	Nb patients NEUTRAL	Majority
ENSG00000088367	EPB41L1	3	0	19	NEUTRAL
ENSG00000115353	TACR1	0	0	22	NEUTRAL
ENSG00000109667	SLC2A9	0	0	22	NEUTRAL
ENSG00000170365	SMAD1	0	1	21	NEUTRAL
ENSG00000102189	EEA1	0	0	22	NEUTRAL
ENSG00000134030	CTIF	1	2	19	NEUTRAL
ENSG00000118690	ARMC2	0	7	15	NEUTRAL
ENSG00000182979	MTA1	0	0	22	NEUTRAL
ENSG00000173546	CSPG4	0	3	19	NEUTRAL
ENSG00000166398	KIAA0355	0	0	22	NEUTRAL
ENSG00000100151	PICK1	0	6	16	NEUTRAL
ENSG00000151948	GLT1D1	0	0	22	NEUTRAL
ENSG00000072201	LNX1	0	0	22	NEUTRAL
ENSG00000116833	NR5A2	14	0	8	AMP
ENSG00000172164	SNTB1	9	0	13	NEUTRAL
ENSG00000188729	OSTN	1	1	20	NEUTRAL
ENSG00000135720	DYNC1LI2	1	13	8	DEL
ENSG00000144792	ZNF660	0	0	22	NEUTRAL
ENSG00000108511	HOXB6	3	2	17	NEUTRAL
ENSG00000183098	GPC6	0	3	19	NEUTRAL
ENSG00000070778	PTPN21	0	1	21	NEUTRAL
ENSG00000106772	PRUNE2	0	1	21	NEUTRAL
ENSG00000213121	AL590867.1	0	3	19	NEUTRAL
ENSG00000160886	LY6K	8	1	13	NEUTRAL
ENSG00000132481	TRIM47	1	2	19	NEUTRAL
ENSG00000189056	RELN	3	1	18	NEUTRAL
ENSG00000224470	ATXN1L	1	13	8	DEL
ENSG00000117020	AKT3	14	0	8	AMP
ENSG00000062038	CDH3	1	13	8	DEL
ENSG00000243335	KCTD7	3	0	19	NEUTRAL
ENSG00000234769	WASH4P	0	0	22	NEUTRAL
ENSG00000154330	PGM5	0	0	22	NEUTRAL
ENSG00000126217	MCF2L	0	3	19	NEUTRAL
ENSG00000214140	PRCD	1	2	19	NEUTRAL
ENSG00000106069	CHN2	4	1	17	NEUTRAL
ENSG00000171243	SOSTDC1	3	1	18	NEUTRAL
ENSG00000137766	UNC13C	0	3	19	NEUTRAL
ENSG00000173011	TADA2B	0	0	22	NEUTRAL
ENSG00000157570	TSPAN18	2	1	19	NEUTRAL
ENSG00000183801	OLFML1	1	2	19	NEUTRAL
ENSG00000072952	MRVI1	2	2	18	NEUTRAL
ENSG00000159176	CSRP1	15	0	7	AMP
ENSG00000052841	TTC17	2	1	19	NEUTRAL
ENSG00000167302	ENTHD2	1	2	19	NEUTRAL
ENSG00000148339	SLC25A25	0	0	22	NEUTRAL
ENSG00000127314	RAP1B	0	0	22	NEUTRAL
ENSG00000115286	NDUFS7	0	2	20	NEUTRAL
ENSG00000173171	MTX1	15	0	7	AMP
ENSG00000196569	LAMA2	0	5	17	NEUTRAL
ENSG00000112584	FAM120B	0	4	18	NEUTRAL
ENSG00000170634	ACYP2	0	0	22	NEUTRAL
ENSG00000152022	LIX1L	15	0	7	AMP

Ensembl	Gene	Nb patients AMP	Nb patients DEL	Nb patients NEUTRAL	Majority
ENSG00000232859	LYRM9	1	2	19	NEUTRAL
ENSG00000196296	ATP2A1	12	0	10	AMP
ENSG00000146433	TMEM181	0	4	18	NEUTRAL
ENSG00000121879	PIK3CA	1	1	20	NEUTRAL
ENSG00000049246	PER3	1	2	19	NEUTRAL
ENSG00000113555	PCDH12	0	2	20	NEUTRAL
ENSG00000171757	LRRC34	1	1	20	NEUTRAL
ENSG00000178104	PDE4DIP	0	0	22	NEUTRAL
ENSG00000250588	IQCJ-SCHIP1	1	2	19	NEUTRAL
ENSG00000136011	STAB2	0	0	22	NEUTRAL
ENSG00000138758	SEPT11	1	0	21	NEUTRAL
ENSG00000110841	PPFIBP1	0	0	22	NEUTRAL
ENSG00000117298	ECE1	1	3	18	NEUTRAL
ENSG00000196371	FUT4	1	5	16	NEUTRAL
ENSG00000181722	ZBTB20	2	1	19	NEUTRAL
ENSG00000066230	SLC9A3	3	1	18	NEUTRAL
ENSG00000155714	PDZD9	12	0	10	AMP
ENSG00000157150	TIMP4	0	0	22	NEUTRAL
ENSG00000231360	AL592284.1	0	0	22	NEUTRAL
ENSG00000178974	FBXO34	0	1	21	NEUTRAL
ENSG00000258653	RP5-1021I20.4	0	1	21	NEUTRAL
ENSG00000146555	SDK1	3	1	18	NEUTRAL
ENSG00000145391	SETD7	0	1	21	NEUTRAL
ENSG00000161103	AC008132.13	0	6	16	NEUTRAL
ENSG00000039523	FAM65A	1	13	8	DEL
ENSG00000121104	FAM117A	3	2	17	NEUTRAL
ENSG00000183579	ZNRF3	0	6	16	NEUTRAL
ENSG00000163646	CLRN1	1	1	20	NEUTRAL
ENSG00000122592	HOXA7	4	1	17	NEUTRAL
ENSG00000176692	FOXC2	1	12	9	DEL
ENSG00000215021	PHB2	0	1	21	NEUTRAL
ENSG00000243927	MRPS6	2	0	20	NEUTRAL
ENSG00000198838	RYR3	0	2	20	NEUTRAL
ENSG00000112561	TFEB	0	1	21	NEUTRAL
ENSG00000166548	TK2	1	13	8	DEL
ENSG00000267281	RP11-793H13.10	0	0	22	NEUTRAL
ENSG00000104067	TJP1	0	1	21	NEUTRAL
ENSG00000095066	HOOK2	0	2	20	NEUTRAL
ENSG00000159110	IFNAR2	2	0	20	NEUTRAL
ENSG00000182132	KCNIP1	0	2	20	NEUTRAL
ENSG00000167671	UBXN6	1	1	20	NEUTRAL
ENSG00000183439	TRIM61	0	1	21	NEUTRAL
ENSG00000168306	ACOX2	0	0	22	NEUTRAL
ENSG00000105894	PTN	3	1	18	NEUTRAL
ENSG00000113580	NR3C1	0	2	20	NEUTRAL
ENSG00000165409	TSHR	0	1	21	NEUTRAL
ENSG00000145332	KLHL8	0	1	21	NEUTRAL
ENSG00000090905	TNRC6A	12	0	10	AMP
ENSG00000076984	MAP2K7	1	1	20	NEUTRAL
ENSG00000141384	TAF4B	1	1	20	NEUTRAL
ENSG00000154511	FAM69A	0	1	21	NEUTRAL
ENSG00000163659	TIPARP	1	2	19	NEUTRAL

Ensembl	Gene	Nb patients AMP	Nb patients DEL	Nb patients NEUTRAL	Majority
ENSG00000198643	FAM3D	0	0	22	NEUTRAL
ENSG00000132692	BCAN	15	0	7	AMP
ENSG00000182704	TSKU	3	2	17	NEUTRAL
ENSG00000087884	AAMDC	4	2	16	NEUTRAL
ENSG00000048471	SNX29	12	0	10	AMP
ENSG00000153993	SEMA3D	3	0	19	NEUTRAL
ENSG00000106100	NOD1	4	1	17	NEUTRAL
ENSG00000105371	ICAM4	1	1	20	NEUTRAL
ENSG00000164411	GJB7	0	7	15	NEUTRAL
ENSG00000100154	TTC28	0	6	16	NEUTRAL
ENSG00000138411	HECW2	0	0	22	NEUTRAL
ENSG00000133706	LARS	0	2	20	NEUTRAL
ENSG00000176406	RIMS2	9	0	13	NEUTRAL
ENSG00000151914	DST	1	0	21	NEUTRAL
ENSG00000080561	MID2	2	2	18	NEUTRAL
ENSG00000107796	ACTA2	0	2	20	NEUTRAL
ENSG00000008710	PKD1	12	0	10	AMP
ENSG00000113594	LIFR	2	0	20	NEUTRAL
ENSG00000100439	ABHD4	0	0	22	NEUTRAL
ENSG00000136842	TMOD1	0	1	21	NEUTRAL
ENSG00000078070	MCCC1	1	1	20	NEUTRAL
ENSG00000106333	PCOLCE	4	0	18	NEUTRAL
ENSG00000108823	SGCA	3	2	17	NEUTRAL
ENSG00000125633	CCDC93	0	0	22	NEUTRAL
ENSG00000182095	TNRC18	3	1	18	NEUTRAL
ENSG00000152284	TCF7L1	0	0	22	NEUTRAL
ENSG00000182732	RGS6	0	1	21	NEUTRAL
ENSG00000149294	NCAM1	1	6	15	NEUTRAL
ENSG00000168748	CA7	1	13	8	DEL
ENSG00000138131	LOXL4	0	1	21	NEUTRAL
ENSG00000171992	SYNPO	0	2	20	NEUTRAL
ENSG00000189292	FAM150B	0	0	22	NEUTRAL
ENSG00000109466	KLHL2	0	1	21	NEUTRAL
ENSG00000082438	COBLL1	0	1	21	NEUTRAL
ENSG00000148655	C10orf11	1	0	21	NEUTRAL
ENSG00000164123	C4orf45	0	1	21	NEUTRAL
ENSG00000144668	ITGA9	0	0	22	NEUTRAL
ENSG00000143140	GJA5	15	0	7	AMP
ENSG00000165895	ARHGAP42	0	5	17	NEUTRAL
ENSG00000180357	ZNF609	0	3	19	NEUTRAL
ENSG00000184545	DUSP8	1	2	19	NEUTRAL
ENSG00000079313	REXO1	0	2	20	NEUTRAL
ENSG00000069667	RORA	0	3	19	NEUTRAL
ENSG00000104529	EEF1D	8	1	13	NEUTRAL
ENSG00000140577	CRTC3	0	2	20	NEUTRAL
ENSG00000064726	BTBD1	0	2	20	NEUTRAL
ENSG00000170537	TMC7	12	0	10	AMP
ENSG00000198668	CALM1	0	1	21	NEUTRAL
ENSG00000099949	LZTR1	0	6	16	NEUTRAL
ENSG00000140299	BNIP2	0	3	19	NEUTRAL
ENSG00000081189	MEF2C	0	2	20	NEUTRAL
ENSG00000067248	DHX29	0	0	22	NEUTRAL

Ensembl	Gene	Nb patients AMP	Nb patients DEL	Nb patients NEUTRAL	Majority
ENSG00000132819	RBM38	3	0	19	NEUTRAL
ENSG00000118197	DDX59	14	0	8	AMP
ENSG00000187391	MAGI2	3	0	19	NEUTRAL
ENSG00000164037	SLC9B1	0	1	21	NEUTRAL
ENSG00000172264	MACROD2	4	0	18	NEUTRAL
ENSG00000120910	PPP3CC	4	7	11	NEUTRAL
ENSG00000171451	DSEL	2	1	19	NEUTRAL
ENSG00000100234	TIMP3	0	6	16	NEUTRAL
ENSG00000063046	EIF4B	0	0	22	NEUTRAL
ENSG00000151692	RNF144A	0	0	22	NEUTRAL
ENSG00000166181	API5	2	1	19	NEUTRAL
ENSG00000117859	OSBPL9	0	2	20	NEUTRAL
ENSG00000148143	ZNF462	0	1	21	NEUTRAL
ENSG00000118257	NRP2	0	0	22	NEUTRAL
ENSG00000100142	POLR2F	0	6	16	NEUTRAL
ENSG00000154710	RABGEF1	3	0	19	NEUTRAL
ENSG00000184060	ADAP2	1	2	19	NEUTRAL
ENSG00000105379	ETFB	1	1	20	NEUTRAL
ENSG00000198092	TMPRSS11F	0	0	22	NEUTRAL
ENSG00000112406	HECA	0	6	16	NEUTRAL
ENSG00000162669	HFM1	0	1	21	NEUTRAL
ENSG00000182568	SATB1	0	0	22	NEUTRAL
ENSG00000187555	USP7	13	0	9	AMP
ENSG00000138594	TMOD3	0	3	19	NEUTRAL
ENSG00000183018	SPNS2	0	5	17	NEUTRAL
ENSG00000103187	COTL1	1	12	9	DEL
ENSG00000158828	PINK1	1	3	18	NEUTRAL
ENSG00000087460	GNAS	3	0	19	NEUTRAL
ENSG00000150938	CRIM1	0	0	22	NEUTRAL
ENSG00000129993	CBFA2T3	1	12	9	DEL
ENSG00000169762	TAPT1	0	0	22	NEUTRAL
ENSG00000077157	PPP1R12B	15	0	7	AMP
ENSG00000082641	NFE2L1	3	2	17	NEUTRAL
ENSG00000154162	CDH12	2	0	20	NEUTRAL
ENSG00000148841	ITPRIP	0	1	21	NEUTRAL
ENSG00000143479	DYRK3	15	0	7	AMP
ENSG00000128591	FLNC	3	1	18	NEUTRAL
ENSG00000138101	DTNB	0	0	22	NEUTRAL
ENSG00000150760	DOCK1	0	0	22	NEUTRAL
ENSG00000174903	RAB1B	3	3	16	NEUTRAL
ENSG00000186868	MAPT	2	3	17	NEUTRAL
ENSG00000182950	ODF3L1	0	3	19	NEUTRAL
ENSG00000105971	CAV2	3	1	18	NEUTRAL
ENSG00000162383	SLC1A7	0	2	20	NEUTRAL
ENSG00000065809	FAM107B	0	1	21	NEUTRAL
ENSG00000215790	SLC35E2	1	2	19	NEUTRAL
ENSG00000205592	MUC19	0	0	22	NEUTRAL
ENSG00000135972	MRPS9	0	0	22	NEUTRAL

Supplemental File 1 /Table S9 : DiffExp list of NAT/PC pairs. In this list, PC expression is potentially regulated by their corresponding NAT. The genomic copy number status is indicated.

Differential expression analysis between all tumor and healthy samples was performed with the DESeq2 software (v. 1.10.1), following the standard workflow. Pairs of protein coding/antisense genes where the antisense was significantly differentially expressed (adjusted p-value < 0.05) between normal and tumor samples have been selected. Pairs of genes where either the protein-coding or the antisense was expressed in less than 7 tumor samples or 7 healthy samples have

Ensembl	Gene	Nb patients AMP	Nb patients DEL	Nb patients NEUTRAL	Majority
ENSG00000004897	CDC27	2	2	18	NEUTRAL
ENSG00000005001	PRSS22	13	0	9	AMP
ENSG00000005022	SLC25A5	2	2	18	NEUTRAL
ENSG00000005108	THSD7A	3	1	18	NEUTRAL
ENSG00000005436	GCFC2	0	0	22	NEUTRAL
ENSG00000006007	GDE1	12	0	10	AMP
ENSG00000006534	ALDH3B1	2	1	19	NEUTRAL
ENSG00000006652	IFRD1	4	1	17	NEUTRAL
ENSG00000006837	CDKL3	0	2	20	NEUTRAL
ENSG00000008517	IL32	13	0	9	AMP
ENSG00000008853	RHOBTB2	4	7	11	NEUTRAL
ENSG00000009413	REV3L	0	7	15	NEUTRAL
ENSG00000011566	MAP4K3	0	0	22	NEUTRAL
ENSG00000013364	MVP	12	0	10	AMP
ENSG00000013573	DDX11	0	0	22	NEUTRAL
ENSG00000013583	HEBP1	0	1	21	NEUTRAL
ENSG00000018189	RUFY3	0	0	22	NEUTRAL
ENSG00000023287	RB1CC1	7	1	14	NEUTRAL
ENSG00000029534	ANK1	7	3	12	NEUTRAL
ENSG00000049540	ELN	3	0	19	NEUTRAL
ENSG00000050344	NFE2L3	4	1	17	NEUTRAL
ENSG00000053254	FOXN3	0	1	21	NEUTRAL
ENSG00000054392	HHAT	15	0	7	AMP
ENSG00000054965	FAM168A	3	1	18	NEUTRAL
ENSG00000055609	KMT2C	4	0	18	NEUTRAL
ENSG00000055813	CCDC85A	0	0	22	NEUTRAL
ENSG00000056972	TRAF3IP2	0	7	15	NEUTRAL
ENSG00000058272	PPP1R12A	0	0	22	NEUTRAL
ENSG00000059122	FLYWCH1	13	0	9	AMP
ENSG00000061938	TNK2	1	1	20	NEUTRAL
ENSG00000062282	DGAT2	3	1	18	NEUTRAL
ENSG00000062485	CS	0	0	22	NEUTRAL
ENSG00000063046	EIF4B	0	0	22	NEUTRAL
ENSG00000064652	SNX24	0	2	20	NEUTRAL
ENSG00000065526	SPEN	1	3	18	NEUTRAL
ENSG00000065534	MYLK	1	2	19	NEUTRAL
ENSG00000066135	KDM4A	0	2	20	NEUTRAL
ENSG00000066933	MYO9A	0	2	20	NEUTRAL
ENSG00000067248	DHX29	0	0	22	NEUTRAL
ENSG00000067606	PRKCZ	1	2	19	NEUTRAL
ENSG00000068489	PRR11	2	1	19	NEUTRAL
ENSG00000070010	UFD1L	0	6	16	NEUTRAL
ENSG00000070540	WIPI1	2	1	19	NEUTRAL

Ensembl	Gene	Nb patients AMP	Nb patients DEL	Nb patients NEUTRAL	Majority
ENSG00000070731	ST6GALNAC2	1	2	19	NEUTRAL
ENSG00000071051	NCK2	0	0	22	NEUTRAL
ENSG00000071909	MYO3B	0	1	21	NEUTRAL
ENSG00000072657	TRHDE	0	0	22	NEUTRAL
ENSG00000072736	NFATC3	1	13	8	DEL
ENSG00000073008	PVR	0	1	21	NEUTRAL
ENSG00000073803	MAP3K13	1	1	20	NEUTRAL
ENSG00000073910	FRY	0	3	19	NEUTRAL
ENSG00000074054	CLASP1	0	0	22	NEUTRAL
ENSG00000074590	NUAK1	0	0	22	NEUTRAL
ENSG00000074803	SLC12A1	0	3	19	NEUTRAL
ENSG00000075089	ACTR6	0	0	22	NEUTRAL
ENSG00000075461	CACNG4	2	1	19	NEUTRAL
ENSG00000075568	TMEM131	0	0	22	NEUTRAL
ENSG00000076944	STXBP2	1	1	20	NEUTRAL
ENSG00000078114	NEBL	0	1	21	NEUTRAL
ENSG00000078177	N4BP2	0	0	22	NEUTRAL
ENSG00000078618	NRD1	0	2	20	NEUTRAL
ENSG00000079313	REXO1	0	2	20	NEUTRAL
ENSG00000079385	CEACAM1	0	1	21	NEUTRAL
ENSG00000079435	LIPE	0	1	21	NEUTRAL
ENSG00000079974	RABL2B	0	0	22	NEUTRAL
ENSG00000080546	SESN1	0	7	15	NEUTRAL
ENSG00000081189	MEF2C	0	2	20	NEUTRAL
ENSG00000081665	ZNF506	0	1	21	NEUTRAL
ENSG00000081923	ATP8B1	1	1	20	NEUTRAL
ENSG00000082014	SMARCD3	4	0	18	NEUTRAL
ENSG00000082458	DLG3	2	2	18	NEUTRAL
ENSG00000083720	OXCT1	1	0	21	NEUTRAL
ENSG00000084090	STARD7	0	0	22	NEUTRAL
ENSG00000085274	MYNN	1	1	20	NEUTRAL
ENSG00000085511	MAP3K4	0	4	18	NEUTRAL
ENSG00000085644	ZNF213	13	0	9	AMP
ENSG00000085831	TTC39A	0	2	20	NEUTRAL
ENSG00000086062	B4GALT1	0	0	22	NEUTRAL
ENSG00000086598	TMED2	0	0	22	NEUTRAL
ENSG00000087152	ATXN7L3	1	3	18	NEUTRAL
ENSG00000087245	MMP2	1	11	10	DEL
ENSG00000087884	AAMDC	4	2	16	NEUTRAL
ENSG00000087903	RFX2	1	1	20	NEUTRAL
ENSG00000088387	DOCK9	0	3	19	NEUTRAL
ENSG00000088756	ARHGAP28	1	2	19	NEUTRAL
ENSG00000088926	F11	0	1	21	NEUTRAL
ENSG00000088986	DYNLL1	0	0	22	NEUTRAL
ENSG00000089159	PXN	0	0	22	NEUTRAL
ENSG00000090376	IRAK3	0	0	22	NEUTRAL
ENSG00000092871	RFFL	1	3	18	NEUTRAL
ENSG00000094755	GABRP	0	2	20	NEUTRAL
ENSG00000094916	CBX5	0	0	22	NEUTRAL
ENSG00000099331	MYO9B	1	1	20	NEUTRAL
ENSG00000099364	FBXL19	12	0	10	AMP

Ensembl	Gene	Nb patients AMP	Nb patients DEL	Nb patients NEUTRAL	Majority
ENSG00000099385	BCL7C	12	0	10	AMP
ENSG00000099999	RNF215	0	6	16	NEUTRAL
ENSG00000100003	SEC14L2	0	6	16	NEUTRAL
ENSG00000100034	PPM1F	0	6	16	NEUTRAL
ENSG00000100151	PICK1	0	6	16	NEUTRAL
ENSG00000100330	MTMR3	0	6	16	NEUTRAL
ENSG00000100802	C14orf93	0	0	22	NEUTRAL
ENSG00000101367	MAPRE1	3	0	19	NEUTRAL
ENSG00000101440	ASIP	3	0	19	NEUTRAL
ENSG00000101577	LPIN2	1	2	19	NEUTRAL
ENSG00000101883	RHOXF1	2	2	18	NEUTRAL
ENSG00000101945	SUV39H1	3	2	17	NEUTRAL
ENSG00000101972	STAG2	2	2	18	NEUTRAL
ENSG00000102189	EEA1	0	0	22	NEUTRAL
ENSG00000102466	FGF14	0	3	19	NEUTRAL
ENSG00000102471	NDFIP2	0	3	19	NEUTRAL
ENSG00000102572	STK24	0	3	19	NEUTRAL
ENSG00000102606	ARHGEF7	0	3	19	NEUTRAL
ENSG00000102879	CORO1A	12	0	10	AMP
ENSG00000103148	NPRL3	12	0	10	AMP
ENSG00000103275	UBE2I	12	0	10	AMP
ENSG00000103351	CLUAP1	13	0	9	AMP
ENSG00000103489	XYLT1	12	0	10	AMP
ENSG00000103495	MAZ	12	0	10	AMP
ENSG00000103852	TTC23	0	0	22	NEUTRAL
ENSG00000104067	TJP1	0	1	21	NEUTRAL
ENSG00000104093	DMXL2	0	3	19	NEUTRAL
ENSG00000104147	OIP5	0	3	19	NEUTRAL
ENSG00000104154	SLC30A4	0	3	19	NEUTRAL
ENSG00000104299	INTS9	4	4	14	NEUTRAL
ENSG00000104490	NCALD	9	0	13	NEUTRAL
ENSG00000104517	UBR5	9	0	13	NEUTRAL
ENSG00000104529	EEF1D	8	1	13	NEUTRAL
ENSG00000104689	TNFRSF10A	4	7	11	NEUTRAL
ENSG00000104885	DOT1L	0	2	20	NEUTRAL
ENSG00000104901	DKKL1	1	1	20	NEUTRAL
ENSG00000105270	CLIP3	0	1	21	NEUTRAL
ENSG00000105339	DENND3	8	1	13	NEUTRAL
ENSG00000105618	PRPF31	2	0	20	NEUTRAL
ENSG00000105778	AVL9	4	1	17	NEUTRAL
ENSG00000105971	CAV2	3	1	18	NEUTRAL
ENSG00000105991	HOXA1	4	1	17	NEUTRAL
ENSG00000105996	HOXA2	4	1	17	NEUTRAL
ENSG00000106069	CHN2	4	1	17	NEUTRAL
ENSG00000106333	PCOLCE	4	0	18	NEUTRAL
ENSG00000106336	FBXO24	4	0	18	NEUTRAL
ENSG00000107077	KDM4C	0	0	22	NEUTRAL
ENSG00000107282	APBA1	0	1	21	NEUTRAL
ENSG00000107854	TNKS2	0	1	21	NEUTRAL
ENSG00000107938	EDRF1	0	0	22	NEUTRAL
ENSG00000107957	SH3PXD2A	0	1	21	NEUTRAL

Ensembl	Gene	Nb patients AMP	Nb patients DEL	Nb patients NEUTRAL	Majority
ENSG00000108309	RUNDC3A	1	3	18	NEUTRAL
ENSG00000108312	UBTF	1	3	18	NEUTRAL
ENSG00000108984	MAP2K6	1	1	20	NEUTRAL
ENSG00000109466	KLHL2	0	1	21	NEUTRAL
ENSG00000109472	CPE	0	1	21	NEUTRAL
ENSG00000109586	GALNT7	0	1	21	NEUTRAL
ENSG00000109667	SLC2A9	0	0	22	NEUTRAL
ENSG00000110328	GALNT18	2	2	18	NEUTRAL
ENSG00000110375	UPK2	0	7	15	NEUTRAL
ENSG00000110693	SOX6	2	2	18	NEUTRAL
ENSG00000110917	MLEC	0	0	22	NEUTRAL
ENSG00000111077	TENC1	0	0	22	NEUTRAL
ENSG00000111269	CREBL2	0	1	21	NEUTRAL
ENSG00000111540	RAB5B	0	0	22	NEUTRAL
ENSG00000111859	NEDD9	0	1	21	NEUTRAL
ENSG00000112530	PACRG	0	4	18	NEUTRAL
ENSG00000112769	LAMA4	0	7	15	NEUTRAL
ENSG00000113108	APBB3	0	2	20	NEUTRAL
ENSG00000113161	HMGCR	0	1	21	NEUTRAL
ENSG00000113594	LIFR	2	0	20	NEUTRAL
ENSG00000113971	NPHP3	1	1	20	NEUTRAL
ENSG00000114113	RBP2	1	1	20	NEUTRAL
ENSG00000114115	RBP1	1	1	20	NEUTRAL
ENSG00000114670	NEK11	1	2	19	NEUTRAL
ENSG00000114956	DGUOK	0	0	22	NEUTRAL
ENSG00000115207	GTF3C2	0	0	22	NEUTRAL
ENSG00000115211	EIF2B4	0	0	22	NEUTRAL
ENSG00000115233	PSMD14	0	1	21	NEUTRAL
ENSG00000115286	NDUFS7	0	2	20	NEUTRAL
ENSG00000115504	EHBP1	0	0	22	NEUTRAL
ENSG00000115866	DARS	1	1	20	NEUTRAL
ENSG00000115896	PLCL1	0	0	22	NEUTRAL
ENSG00000116141	MARK1	15	0	7	AMP
ENSG00000116171	SCP2	0	2	20	NEUTRAL
ENSG00000116539	ASH1L	15	0	7	AMP
ENSG00000116580	GON4L	15	0	7	AMP
ENSG00000116584	ARHGEF2	15	0	7	AMP
ENSG00000116754	SRSF11	0	1	21	NEUTRAL
ENSG00000116977	LGALS8	14	0	8	AMP
ENSG00000117054	ACADM	1	1	20	NEUTRAL
ENSG00000117091	CD48	14	0	8	AMP
ENSG00000117594	HSD11B1	15	0	7	AMP
ENSG00000118197	DDX59	14	0	8	AMP
ENSG00000118276	B4GALT6	2	1	19	NEUTRAL
ENSG00000118418	HMGN3	0	5	17	NEUTRAL
ENSG00000118523	CTGF	0	5	17	NEUTRAL
ENSG00000118971	CCND2	0	1	21	NEUTRAL
ENSG00000119537	KDSR	1	1	20	NEUTRAL
ENSG00000120055	C10orf95	0	1	21	NEUTRAL
ENSG00000120071	KANSL1	2	2	18	NEUTRAL
ENSG00000120645	IQSEC3	0	0	22	NEUTRAL

Ensembl	Gene	Nb patients AMP	Nb patients DEL	Nb patients NEUTRAL	Majority
ENSG00000120802	TMPO	0	0	22	NEUTRAL
ENSG00000120833	SOCS2	0	0	22	NEUTRAL
ENSG00000120896	SORBS3	4	7	11	NEUTRAL
ENSG00000120910	PPP3CC	4	7	11	NEUTRAL
ENSG00000121068	TBX2	2	1	19	NEUTRAL
ENSG00000121361	KCNJ8	0	1	21	NEUTRAL
ENSG00000121410	A1BG	0	0	22	NEUTRAL
ENSG00000121440	PDZRN3	0	0	22	NEUTRAL
ENSG00000121454	LHX4	14	0	8	AMP
ENSG00000121716	PILRB	4	0	18	NEUTRAL
ENSG00000122386	ZNF205	13	0	9	AMP
ENSG00000122691	TWIST1	3	1	18	NEUTRAL
ENSG00000122705	CLTA	0	0	22	NEUTRAL
ENSG00000122783	C7orf49	3	1	18	NEUTRAL
ENSG00000123124	WWP1	9	0	13	NEUTRAL
ENSG00000123159	GIPC1	0	2	20	NEUTRAL
ENSG00000123405	NFE2	0	0	22	NEUTRAL
ENSG00000123416	TUBA1B	0	0	22	NEUTRAL
ENSG00000123689	G0S2	15	0	7	AMP
ENSG00000123700	KCNJ2	2	1	19	NEUTRAL
ENSG00000123992	DNPEP	0	0	22	NEUTRAL
ENSG00000124214	STAU1	3	0	19	NEUTRAL
ENSG00000124469	CEACAM8	0	1	21	NEUTRAL
ENSG00000124523	SIRT5	1	0	21	NEUTRAL
ENSG00000124813	RUNX2	0	0	22	NEUTRAL
ENSG00000125398	SOX9	1	1	20	NEUTRAL
ENSG00000125841	NRSN2	4	0	18	NEUTRAL
ENSG00000125966	MMP24	3	0	19	NEUTRAL
ENSG00000126001	CEP250	3	0	19	NEUTRAL
ENSG00000126091	ST3GAL3	0	2	20	NEUTRAL
ENSG00000126777	KTN1	0	1	21	NEUTRAL
ENSG00000127580	WDR24	12	0	10	AMP
ENSG00000127585	FBXL16	12	0	10	AMP
ENSG00000127948	POR	3	0	19	NEUTRAL
ENSG00000128052	KDR	0	0	22	NEUTRAL
ENSG00000128250	RFPL1	0	6	16	NEUTRAL
ENSG00000128298	BAIAP2L2	0	6	16	NEUTRAL
ENSG00000128513	POT1	4	2	16	NEUTRAL
ENSG00000128641	MYO1B	0	0	22	NEUTRAL
ENSG00000128652	HOXD3	0	1	21	NEUTRAL
ENSG00000128694	OSGEPL1	0	0	22	NEUTRAL
ENSG00000128709	HOXD9	0	1	21	NEUTRAL
ENSG00000128908	INO80	0	3	19	NEUTRAL
ENSG00000128923	FAM63B	0	3	19	NEUTRAL
ENSG00000129038	LOXL1	0	2	20	NEUTRAL
ENSG00000129493	HEATR5A	1	0	21	NEUTRAL
ENSG00000129654	FOXJ1	1	2	19	NEUTRAL
ENSG00000129682	FGF13	2	2	18	NEUTRAL
ENSG00000129911	KLF16	0	2	20	NEUTRAL
ENSG00000129925	TMEM8A	12	0	10	AMP
ENSG00000130584	ZBTB46	3	0	19	NEUTRAL

Ensembl	Gene	Nb patients AMP	Nb patients DEL	Nb patients NEUTRAL	Majority
ENSG00000130684	ZNF337	2	0	20	NEUTRAL
ENSG00000130770	ATPIF1	1	4	17	NEUTRAL
ENSG00000130813	C19orf66	1	1	20	NEUTRAL
ENSG00000130957	FBP2	0	1	21	NEUTRAL
ENSG00000131408	NR1H2	1	1	20	NEUTRAL
ENSG00000131477	RAMP2	1	4	17	NEUTRAL
ENSG00000131504	DIAPH1	0	2	20	NEUTRAL
ENSG00000131781	FMO5	15	0	7	AMP
ENSG00000132122	SPATA6	0	2	20	NEUTRAL
ENSG00000132326	PER2	0	1	21	NEUTRAL
ENSG00000132405	TBC1D14	0	0	22	NEUTRAL
ENSG00000132466	ANKRD17	0	0	22	NEUTRAL
ENSG00000132481	TRIM47	1	2	19	NEUTRAL
ENSG00000132692	BCAN	15	0	7	AMP
ENSG00000132846	ZBED3	0	1	21	NEUTRAL
ENSG00000132874	SLC14A2	2	2	18	NEUTRAL
ENSG00000133112	TPT1	0	4	18	NEUTRAL
ENSG00000133256	PDE6B	0	0	22	NEUTRAL
ENSG00000133275	CSNK1G2	0	2	20	NEUTRAL
ENSG00000133657	ATP13A3	1	1	20	NEUTRAL
ENSG00000133661	SFTPD	1	0	21	NEUTRAL
ENSG00000134013	LOXL2	4	7	11	NEUTRAL
ENSG00000134020	PEBP4	4	7	11	NEUTRAL
ENSG00000134121	CHL1	0	0	22	NEUTRAL
ENSG00000134256	CD101	1	2	19	NEUTRAL
ENSG00000134259	NGF	1	2	19	NEUTRAL
ENSG00000134508	CABLES1	1	2	19	NEUTRAL
ENSG00000134871	COL4A2	0	3	19	NEUTRAL
ENSG00000134970	TMED7	0	2	20	NEUTRAL
ENSG00000135018	UBQLN1	0	1	21	NEUTRAL
ENSG00000135269	TES	3	1	18	NEUTRAL
ENSG00000135404	CD63	0	0	22	NEUTRAL
ENSG00000135547	HEY2	0	6	16	NEUTRAL
ENSG00000135577	NMBR	0	5	17	NEUTRAL
ENSG00000135679	MDM2	0	0	22	NEUTRAL
ENSG00000135862	LAMC1	14	0	8	AMP
ENSG00000136244	IL6	4	1	17	NEUTRAL
ENSG00000136367	ZFHX2	1	0	21	NEUTRAL
ENSG00000137154	RPS6	0	0	22	NEUTRAL
ENSG00000137203	TFAP2A	1	1	20	NEUTRAL
ENSG00000137642	SORL1	0	7	15	NEUTRAL
ENSG00000137674	MMP20	0	5	17	NEUTRAL
ENSG00000137726	FXYD6	0	7	15	NEUTRAL
ENSG00000137801	THBS1	0	3	19	NEUTRAL
ENSG00000137807	KIF23	0	2	20	NEUTRAL
ENSG00000137819	PAQR5	0	2	20	NEUTRAL
ENSG00000138031	ADCY3	0	0	22	NEUTRAL
ENSG00000138061	CYP1B1	0	0	22	NEUTRAL
ENSG00000138101	DTNB	0	0	22	NEUTRAL
ENSG00000138594	TMOD3	0	3	19	NEUTRAL
ENSG00000138617	PARP16	0	3	19	NEUTRAL

Ensembl	Gene	Nb patients AMP	Nb patients DEL	Nb patients NEUTRAL	Majority
ENSG00000138641	HERC3	0	1	21	NEUTRAL
ENSG00000138795	LEF1	0	1	21	NEUTRAL
ENSG00000139151	PLCZ1	0	1	21	NEUTRAL
ENSG00000139182	CLSTN3	0	1	21	NEUTRAL
ENSG00000139572	GPR84	0	0	22	NEUTRAL
ENSG00000139624	CERS5	0	0	22	NEUTRAL
ENSG00000139746	RBM26	0	3	19	NEUTRAL
ENSG00000140199	SLC12A6	0	2	20	NEUTRAL
ENSG00000140455	USP3	0	3	19	NEUTRAL
ENSG00000140548	ZNF710	0	2	20	NEUTRAL
ENSG00000140577	CRTC3	0	2	20	NEUTRAL
ENSG00000140650	PMM2	13	0	9	AMP
ENSG00000140691	ARMC5	12	0	10	AMP
ENSG00000140749	IGSF6	12	0	10	AMP
ENSG00000140945	CDH13	1	12	9	DEL
ENSG00000141293	SKAP1	3	2	17	NEUTRAL
ENSG00000141527	CARD14	1	2	19	NEUTRAL
ENSG00000141576	RNF157	1	2	19	NEUTRAL
ENSG00000142185	TRPM2	2	0	20	NEUTRAL
ENSG00000142192	APP	2	0	20	NEUTRAL
ENSG00000142512	SIGLEC10	1	1	20	NEUTRAL
ENSG00000142937	RPS8	0	2	20	NEUTRAL
ENSG00000142945	KIF2C	0	2	20	NEUTRAL
ENSG00000143183	TMCO1	14	0	8	AMP
ENSG00000143520	FLG2	15	0	7	AMP
ENSG00000143570	SLC39A1	15	0	7	AMP
ENSG00000143631	FLG	15	0	7	AMP
ENSG00000143819	EPHX1	15	0	7	AMP
ENSG00000143858	SYT2	15	0	7	AMP
ENSG00000144218	AFF3	0	0	22	NEUTRAL
ENSG00000144339	TMEFF2	0	1	21	NEUTRAL
ENSG00000144452	ABCA12	0	0	22	NEUTRAL
ENSG00000144485	HES6	0	1	21	NEUTRAL
ENSG00000144642	RBMS3	0	0	22	NEUTRAL
ENSG00000144744	UBA3	0	0	22	NEUTRAL
ENSG00000144747	TMF1	0	0	22	NEUTRAL
ENSG00000144792	ZNF660	0	0	22	NEUTRAL
ENSG00000144908	ALDH1L1	1	2	19	NEUTRAL
ENSG00000145012	LPP	1	1	20	NEUTRAL
ENSG00000145416	MARCH1	0	1	21	NEUTRAL
ENSG00000145934	TENM2	0	2	20	NEUTRAL
ENSG00000145936	KCNMB1	0	2	20	NEUTRAL
ENSG00000146122	DAAM2	0	1	21	NEUTRAL
ENSG00000146373	RNF217	0	6	16	NEUTRAL
ENSG00000146433	TMEM181	0	4	18	NEUTRAL
ENSG00000146828	SLC12A9	4	0	18	NEUTRAL
ENSG00000147113	CXorf36	2	2	18	NEUTRAL
ENSG00000147246	HTR2C	2	2	18	NEUTRAL
ENSG00000147419	CCDC25	4	5	13	NEUTRAL
ENSG00000147655	RSPO2	9	0	13	NEUTRAL
ENSG00000147687	TATDN1	9	0	13	NEUTRAL

Ensembl	Gene	Nb patients AMP	Nb patients DEL	Nb patients NEUTRAL	Majority
ENSG00000147852	VLDLR	0	0	22	NEUTRAL
ENSG00000147862	NFIB	0	0	22	NEUTRAL
ENSG00000147883	CDKN2B	0	0	22	NEUTRAL
ENSG00000147889	CDKN2A	0	0	22	NEUTRAL
ENSG00000148482	SLC39A12	0	1	21	NEUTRAL
ENSG00000148660	CAMK2G	0	0	22	NEUTRAL
ENSG00000148671	ADIRF	0	2	20	NEUTRAL
ENSG00000149256	TENM4	3	2	17	NEUTRAL
ENSG00000149346	SLX4IP	4	0	18	NEUTRAL
ENSG00000150687	PRSS23	1	4	17	NEUTRAL
ENSG00000151364	KCTD14	4	2	16	NEUTRAL
ENSG00000151623	NR3C2	0	1	21	NEUTRAL
ENSG00000151692	RNF144A	0	0	22	NEUTRAL
ENSG00000151702	FLI1	0	7	15	NEUTRAL
ENSG00000151789	ZNF385D	0	0	22	NEUTRAL
ENSG00000152348	ATG10	0	1	21	NEUTRAL
ENSG00000152380	FAM151B	0	1	21	NEUTRAL
ENSG00000152527	PLEKHH2	0	0	22	NEUTRAL
ENSG00000152591	DSPP	0	1	21	NEUTRAL
ENSG00000152592	DMP1	0	1	21	NEUTRAL
ENSG00000152601	MBNL1	1	1	20	NEUTRAL
ENSG00000153147	SMARCA5	0	1	21	NEUTRAL
ENSG00000153283	CD96	2	1	19	NEUTRAL
ENSG00000153802	TMPRSS11D	0	0	22	NEUTRAL
ENSG00000153936	HS2ST1	0	1	21	NEUTRAL
ENSG00000154162	CDH12	2	0	20	NEUTRAL
ENSG00000154305	MIA3	15	0	7	AMP
ENSG00000154330	PGM5	0	0	22	NEUTRAL
ENSG00000154358	OBSCN	15	0	7	AMP
ENSG00000154611	PSMA8	1	1	20	NEUTRAL
ENSG00000154655	L3MBTL4	1	2	19	NEUTRAL
ENSG00000154710	RABGEF1	3	0	19	NEUTRAL
ENSG00000154767	XPC	0	0	22	NEUTRAL
ENSG00000155016	CYP2U1	0	1	21	NEUTRAL
ENSG00000155096	AZIN1	9	0	13	NEUTRAL
ENSG00000155380	SLC16A1	0	2	20	NEUTRAL
ENSG00000155657	TTN	0	1	21	NEUTRAL
ENSG00000155893	ACPL2	1	1	20	NEUTRAL
ENSG00000156030	ELMSAN1	0	1	21	NEUTRAL
ENSG00000156172	C8orf37	9	0	13	NEUTRAL
ENSG00000156269	NAA11	0	1	21	NEUTRAL
ENSG00000156802	ATAD2	9	0	13	NEUTRAL
ENSG00000157077	ZFYVE9	0	2	20	NEUTRAL
ENSG00000157107	FCHO2	0	1	21	NEUTRAL
ENSG00000157110	RBPMS	4	4	14	NEUTRAL
ENSG00000157150	TIMP4	0	0	22	NEUTRAL
ENSG00000157212	PAXIP1	4	0	18	NEUTRAL
ENSG00000157326	DHRS4	0	0	22	NEUTRAL
ENSG00000157778	PSMG3	3	1	18	NEUTRAL
ENSG00000158201	ABHD3	1	1	20	NEUTRAL
ENSG00000158315	RHBDL2	0	3	19	NEUTRAL

Ensembl	Gene	Nb patients AMP	Nb patients DEL	Nb patients NEUTRAL	Majority
ENSG00000158623	COPG2	3	1	18	NEUTRAL
ENSG00000158717	RNF166	2	12	8	DEL
ENSG00000158941	CCAR2	4	7	11	NEUTRAL
ENSG00000159176	CSRP1	15	0	7	AMP
ENSG00000159231	CBR3	2	0	20	NEUTRAL
ENSG00000159261	CLDN14	2	0	20	NEUTRAL
ENSG00000159267	HLCS	2	0	20	NEUTRAL
ENSG00000159399	HK2	0	0	22	NEUTRAL
ENSG00000159479	MED8	0	2	20	NEUTRAL
ENSG00000159658	EFCAB14	0	2	20	NEUTRAL
ENSG00000159692	CTBP1	0	0	22	NEUTRAL
ENSG00000160191	PDE9A	2	0	20	NEUTRAL
ENSG00000160294	MCM3AP	2	0	20	NEUTRAL
ENSG00000160678	S100A1	15	0	7	AMP
ENSG00000160679	CHTOP	15	0	7	AMP
ENSG00000160752	FDPS	15	0	7	AMP
ENSG00000160753	RUSC1	15	0	7	AMP
ENSG00000160917	CPSF4	4	0	18	NEUTRAL
ENSG00000160932	LY6E	8	1	13	NEUTRAL
ENSG00000160972	PPP1R16A	8	1	13	NEUTRAL
ENSG00000161277	THAP8	0	1	21	NEUTRAL
ENSG00000161638	ITGA5	0	0	22	NEUTRAL
ENSG00000161642	ZNF385A	0	0	22	NEUTRAL
ENSG00000161664	ASB16	1	3	18	NEUTRAL
ENSG00000161798	AQP5	0	0	22	NEUTRAL
ENSG00000162009	SSTR5	12	0	10	AMP
ENSG00000162069	CCDC64B	13	0	9	AMP
ENSG00000162365	CYP4A22	0	2	20	NEUTRAL
ENSG00000162543	UBXN10	1	3	18	NEUTRAL
ENSG00000162571	TTLL10	1	2	19	NEUTRAL
ENSG00000162614	NEXN	1	1	20	NEUTRAL
ENSG00000162706	CADM3	14	0	8	AMP
ENSG00000162849	KIF26B	14	0	8	AMP
ENSG00000162929	KIAA1841	0	0	22	NEUTRAL
ENSG00000163041	H3F3A	15	0	7	AMP
ENSG00000163249	CCNYL1	0	0	22	NEUTRAL
ENSG00000163291	PAQR3	0	1	21	NEUTRAL
ENSG00000163357	DCST1	15	0	7	AMP
ENSG00000163374	YY1AP1	15	0	7	AMP
ENSG00000163378	EOGT	0	0	22	NEUTRAL
ENSG00000163435	ELF3	15	0	7	AMP
ENSG00000163625	WDFY3	0	1	21	NEUTRAL
ENSG00000163630	SYNPR	0	0	22	NEUTRAL
ENSG00000163637	PRICKLE2	0	0	22	NEUTRAL
ENSG00000163638	ADAMTS9	0	0	22	NEUTRAL
ENSG00000163645	FAM194A	1	1	20	NEUTRAL
ENSG00000163646	CLRN1	1	1	20	NEUTRAL
ENSG00000163659	TIPARP	1	2	19	NEUTRAL
ENSG00000163704	PRRT3	0	0	22	NEUTRAL
ENSG00000163714	U2SURP	1	1	20	NEUTRAL
ENSG00000163749	CCDC158	1	0	21	NEUTRAL

Ensembl	Gene	Nb patients AMP	Nb patients DEL	Nb patients NEUTRAL	Majority
ENSG00000163864	NMNAT3	1	1	20	NEUTRAL
ENSG00000163975	MFI2	1	1	20	NEUTRAL
ENSG00000164023	SGMS2	0	1	21	NEUTRAL
ENSG00000164048	ZNF589	0	0	22	NEUTRAL
ENSG00000164107	HAND2	0	1	21	NEUTRAL
ENSG00000164125	FAM198B	0	1	21	NEUTRAL
ENSG00000164292	RHOBTB3	0	2	20	NEUTRAL
ENSG00000164307	ERAP1	0	2	20	NEUTRAL
ENSG00000164318	EGFLAM	2	0	20	NEUTRAL
ENSG00000164694	FNDC1	0	4	18	NEUTRAL
ENSG00000164849	GPR146	3	1	18	NEUTRAL
ENSG00000165046	LETM2	9	1	12	NEUTRAL
ENSG00000165072	MAMDC2	0	1	21	NEUTRAL
ENSG00000165092	ALDH1A1	0	1	21	NEUTRAL
ENSG00000165264	NDUFB6	0	0	22	NEUTRAL
ENSG00000165591	FAAH2	2	2	18	NEUTRAL
ENSG00000165659	DACH1	0	3	19	NEUTRAL
ENSG00000165863	C10orf82	0	0	22	NEUTRAL
ENSG00000165868	HSPA12A	0	0	22	NEUTRAL
ENSG00000165966	PDZRN4	0	0	22	NEUTRAL
ENSG00000166037	CEP57	1	5	16	NEUTRAL
ENSG00000166145	SPINT1	0	3	19	NEUTRAL
ENSG00000166598	HSP90B1	0	0	22	NEUTRAL
ENSG00000166734	CASC4	0	3	19	NEUTRAL
ENSG00000166793	YPEL4	0	1	21	NEUTRAL
ENSG00000166801	FAM111A	1	1	20	NEUTRAL
ENSG00000166833	NAV2	2	2	18	NEUTRAL
ENSG00000166900	STX3	2	1	19	NEUTRAL
ENSG00000166947	EPB42	0	3	19	NEUTRAL
ENSG00000166997	CNPY4	4	0	18	NEUTRAL
ENSG00000167371	PRRT2	12	0	10	AMP
ENSG00000167580	AQP2	0	0	22	NEUTRAL
ENSG00000167595	C19orf55	0	1	21	NEUTRAL
ENSG00000167671	UBXN6	1	1	20	NEUTRAL
ENSG00000167701	GPT	8	1	13	NEUTRAL
ENSG00000167716	WDR81	0	5	17	NEUTRAL
ENSG00000167766	ZNF83	1	0	21	NEUTRAL
ENSG00000167772	ANGPTL4	1	1	20	NEUTRAL
ENSG00000167861	HID1	1	2	19	NEUTRAL
ENSG00000167967	E4F1	12	0	10	AMP
ENSG00000167968	DNASE1L2	12	0	10	AMP
ENSG00000167972	ABCA3	12	0	10	AMP
ENSG00000167978	SRRM2	13	0	9	AMP
ENSG00000168288	MMADHC	0	0	22	NEUTRAL
ENSG00000168434	COG7	12	0	10	AMP
ENSG00000168497	SDPR	0	1	21	NEUTRAL
ENSG00000168589	DYNLRB2	1	12	9	DEL
ENSG00000168672	FAM84B	9	0	13	NEUTRAL
ENSG00000168994	PXDC1	0	1	21	NEUTRAL
ENSG00000169067	ACTBL2	0	1	21	NEUTRAL
ENSG00000169231	THBS3	15	0	7	AMP

Ensembl	Gene	Nb patients AMP	Nb patients DEL	Nb patients NEUTRAL	Majority
ENSG00000169282	KCNAB1	1	2	19	NEUTRAL
ENSG00000169744	LDB2	0	0	22	NEUTRAL
ENSG00000169902	TPST1	3	1	18	NEUTRAL
ENSG00000169955	ZNF747	12	0	10	AMP
ENSG00000169994	MYO7B	0	0	22	NEUTRAL
ENSG00000170191	NANP	4	0	18	NEUTRAL
ENSG00000170275	CRTAP	0	0	22	NEUTRAL
ENSG00000170370	EMX2	0	0	22	NEUTRAL
ENSG00000170522	ELOVL6	0	1	21	NEUTRAL
ENSG00000170525	PFKFB3	0	1	21	NEUTRAL
ENSG00000170579	DLGAP1	1	2	19	NEUTRAL
ENSG00000170627	GTSF1	0	0	22	NEUTRAL
ENSG00000170776	AKAP13	0	2	20	NEUTRAL
ENSG00000170921	TANC2	2	1	19	NEUTRAL
ENSG00000171130	ATP6V0E2	4	0	18	NEUTRAL
ENSG00000171451	DSEL	2	1	19	NEUTRAL
ENSG00000171467	ZNF318	0	0	22	NEUTRAL
ENSG00000171484	OR1B1	0	0	22	NEUTRAL
ENSG00000171587	DSCAM	2	0	20	NEUTRAL
ENSG00000171595	DNAI2	1	1	20	NEUTRAL
ENSG00000171720	HDAC3	0	2	20	NEUTRAL
ENSG00000171757	LRRC34	1	1	20	NEUTRAL
ENSG00000171840	NINJ2	0	1	21	NEUTRAL
ENSG00000172270	BSG	0	2	20	NEUTRAL
ENSG00000172315	TP53RK	3	0	19	NEUTRAL
ENSG00000172889	EGFL7	0	0	22	NEUTRAL
ENSG00000172955	ADH6	0	1	21	NEUTRAL
ENSG00000173011	TADA2B	0	0	22	NEUTRAL
ENSG00000173068	BNC2	0	0	22	NEUTRAL
ENSG00000173281	PPP1R3B	3	7	12	NEUTRAL
ENSG00000173889	PHC3	1	1	20	NEUTRAL
ENSG00000173914	RBM4B	4	2	16	NEUTRAL
ENSG00000174255	ZNF80	2	1	19	NEUTRAL
ENSG00000174527	MYO1H	0	0	22	NEUTRAL
ENSG00000174586	ZNF497	0	0	22	NEUTRAL
ENSG00000174939	ASPHD1	12	0	10	AMP
ENSG00000174943	KCTD13	12	0	10	AMP
ENSG00000174996	KLC2	3	3	16	NEUTRAL
ENSG00000175305	CCNE2	9	0	13	NEUTRAL
ENSG00000175356	SCUBE2	1	2	19	NEUTRAL
ENSG00000175536	LIPT2	3	1	18	NEUTRAL
ENSG00000175634	RPS6KB2	2	1	19	NEUTRAL
ENSG00000175746	C15orf54	0	3	19	NEUTRAL
ENSG00000175879	HOXD8	0	1	21	NEUTRAL
ENSG00000176124	DLEU1	0	4	18	NEUTRAL
ENSG00000176177	ENTHD1	0	6	16	NEUTRAL
ENSG00000176390	CRLF3	1	2	19	NEUTRAL
ENSG00000176393	RNPEP	15	0	7	AMP
ENSG00000176595	KBTBD11	3	6	13	NEUTRAL
ENSG00000176635	HORMAD2	0	6	16	NEUTRAL
ENSG00000176658	MYO1D	1	3	18	NEUTRAL

Ensembl	Gene	Nb patients AMP	Nb patients DEL	Nb patients NEUTRAL	Majority
ENSG00000176720	BOK	0	1	21	NEUTRAL
ENSG00000177119	ANO6	0	0	22	NEUTRAL
ENSG00000177311	ZBTB38	1	1	20	NEUTRAL
ENSG00000177479	ARIH2	0	0	22	NEUTRAL
ENSG00000177666	PNPLA2	2	2	18	NEUTRAL
ENSG00000177685	EFCAB4A	2	2	18	NEUTRAL
ENSG00000177697	CD151	2	2	18	NEUTRAL
ENSG00000177732	SOX12	4	0	18	NEUTRAL
ENSG00000177932	ZNF354C	0	1	21	NEUTRAL
ENSG00000177951	BET1L	0	0	22	NEUTRAL
ENSG00000178078	STAP2	1	1	20	NEUTRAL
ENSG00000178184	PARD6G	1	1	20	NEUTRAL
ENSG00000178188	SH2B1	12	0	10	AMP
ENSG00000178467	P4HTM	0	0	22	NEUTRAL
ENSG00000178694	NSUN3	1	0	21	NEUTRAL
ENSG00000178814	OPLAH	8	1	13	NEUTRAL
ENSG00000178828	RNF186	1	3	18	NEUTRAL
ENSG00000178896	EXOSC4	8	1	13	NEUTRAL
ENSG00000179388	EGR3	4	7	11	NEUTRAL
ENSG00000179476	C14orf28	0	1	21	NEUTRAL
ENSG00000179528	LBX2	0	0	22	NEUTRAL
ENSG00000179583	CIITA	13	0	9	AMP
ENSG00000179715	PCED1B	0	0	22	NEUTRAL
ENSG00000179889	PDXDC1	12	0	10	AMP
ENSG00000180098	TRNAU1AP	1	4	17	NEUTRAL
ENSG00000180182	MED14	2	2	18	NEUTRAL
ENSG00000180287	PLD5	14	0	8	AMP
ENSG00000180488	FAM73A	1	1	20	NEUTRAL
ENSG00000180902	D2HGDH	0	1	21	NEUTRAL
ENSG00000181085	MAPK15	8	1	13	NEUTRAL
ENSG00000181092	ADIPOQ	1	1	20	NEUTRAL
ENSG00000181577	C6orf223	0	0	22	NEUTRAL
ENSG00000181788	SIAH2	1	1	20	NEUTRAL
ENSG00000182134	TDRKH	15	0	7	AMP
ENSG00000182253	SYNM	0	0	22	NEUTRAL
ENSG00000182372	CLN8	3	6	13	NEUTRAL
ENSG00000182512	GLRX5	0	1	21	NEUTRAL
ENSG00000182667	NTM	0	7	15	NEUTRAL
ENSG00000182827	ACBD3	15	0	7	AMP
ENSG00000182979	MTA1	0	0	22	NEUTRAL
ENSG00000183010	PYCR1	1	2	19	NEUTRAL
ENSG00000183023	SLC8A1	0	0	22	NEUTRAL
ENSG00000183117	CSMD1	3	6	13	NEUTRAL
ENSG00000183474	GTF2H2C	0	1	21	NEUTRAL
ENSG00000184009	ACTG1	1	2	19	NEUTRAL
ENSG00000184060	ADAP2	1	2	19	NEUTRAL
ENSG00000184347	SLIT3	0	2	20	NEUTRAL
ENSG00000184361	SPATA32	2	3	17	NEUTRAL
ENSG00000184743	ATL3	1	4	17	NEUTRAL
ENSG00000184937	WT1	3	1	18	NEUTRAL
ENSG00000185019	UBOX5	4	0	18	NEUTRAL

Ensembl	Gene	Nb patients AMP	Nb patients DEL	Nb patients NEUTRAL	Majority
ENSG00000185122	HSF1	8	1	13	NEUTRAL
ENSG00000185201	IFITM2	2	2	18	NEUTRAL
ENSG00000185236	RAB11B	1	1	20	NEUTRAL
ENSG00000185278	ZBTB37	14	0	8	AMP
ENSG00000185442	FAM174B	0	2	20	NEUTRAL
ENSG00000185522	C11orf35	2	2	18	NEUTRAL
ENSG00000185551	NR2F2	0	0	22	NEUTRAL
ENSG00000185669	SNAI3	1	12	9	DEL
ENSG00000185928	PAGR1	12	0	10	AMP
ENSG00000186020	ZNF529	0	1	21	NEUTRAL
ENSG00000186047	DLEU7	0	4	18	NEUTRAL
ENSG00000186160	CYP4Z1	0	2	20	NEUTRAL
ENSG00000186193	SAPCD2	0	0	22	NEUTRAL
ENSG00000186340	THBS2	0	4	18	NEUTRAL
ENSG00000186567	CEACAM19	0	1	21	NEUTRAL
ENSG00000186642	PDE2A	3	1	18	NEUTRAL
ENSG00000186868	MAPT	2	3	17	NEUTRAL
ENSG00000187010	RHD	1	4	17	NEUTRAL
ENSG00000187054	TMPRSS11A	0	0	22	NEUTRAL
ENSG00000187109	NAP1L1	0	0	22	NEUTRAL
ENSG00000187140	FOXD3	0	1	21	NEUTRAL
ENSG00000187186	RP11-195F19.5	0	0	22	NEUTRAL
ENSG00000187260	WDR86	4	0	18	NEUTRAL
ENSG00000187391	MAGI2	3	0	19	NEUTRAL
ENSG00000187607	ZNF286A	0	5	17	NEUTRAL
ENSG00000187624	C17orf97	0	5	17	NEUTRAL
ENSG00000187630	DHRS4L2	0	0	22	NEUTRAL
ENSG00000187735	TCEA1	7	0	15	NEUTRAL
ENSG00000187758	ADH1A	0	1	21	NEUTRAL
ENSG00000187824	TMEM220	0	5	17	NEUTRAL
ENSG00000188282	RUFY4	0	0	22	NEUTRAL
ENSG00000188315	C3orf62	0	0	22	NEUTRAL
ENSG00000188316	ENO4	0	0	22	NEUTRAL
ENSG00000188343	FAM92A1	9	0	13	NEUTRAL
ENSG00000188626	GOLGA8M	0	1	21	NEUTRAL
ENSG00000188672	RHCE	1	4	17	NEUTRAL
ENSG00000188997	KCTD21	4	2	16	NEUTRAL
ENSG00000189144	ZNF573	0	1	21	NEUTRAL
ENSG00000189377	CXCL17	0	1	21	NEUTRAL
ENSG00000196090	PTPRT	3	0	19	NEUTRAL
ENSG00000196277	GRM7	0	1	21	NEUTRAL
ENSG00000196296	ATP2A1	12	0	10	AMP
ENSG00000196345	ZKSCAN7	0	0	22	NEUTRAL
ENSG00000196422	PPP1R26	0	0	22	NEUTRAL
ENSG00000196569	LAMA2	0	5	17	NEUTRAL
ENSG00000196642	RABL6	0	0	22	NEUTRAL
ENSG00000196932	TMEM26	1	0	21	NEUTRAL
ENSG00000196935	SRGAP1	0	0	22	NEUTRAL
ENSG00000197013	ZNF429	0	1	21	NEUTRAL
ENSG00000197021	CXorf40B	2	2	18	NEUTRAL
ENSG00000197050	ZNF420	0	1	21	NEUTRAL

Ensembl	Gene	Nb patients AMP	Nb patients DEL	Nb patients NEUTRAL	Majority
ENSG00000197147	LRRC8B	0	1	21	NEUTRAL
ENSG00000197162	ZNF785	12	0	10	AMP
ENSG00000197182	FLJ27365	0	6	16	NEUTRAL
ENSG00000197208	SLC22A4	0	2	20	NEUTRAL
ENSG00000197329	PELI1	0	0	22	NEUTRAL
ENSG00000197361	FBXL22	0	3	19	NEUTRAL
ENSG00000197375	SLC22A5	0	2	20	NEUTRAL
ENSG00000197614	MFAP5	0	1	21	NEUTRAL
ENSG00000197775	DHRS4-AS1	0	0	22	NEUTRAL
ENSG00000197776	KLHDC1	1	0	21	NEUTRAL
ENSG00000197822	OCLN	0	1	21	NEUTRAL
ENSG00000197852	FAM212B	0	2	20	NEUTRAL
ENSG00000197915	HRNR	15	0	7	AMP
ENSG00000198046	ZNF667	2	0	20	NEUTRAL
ENSG00000198092	TMPRSS11F	0	0	22	NEUTRAL
ENSG00000198099	ADH4	0	1	21	NEUTRAL
ENSG00000198168	SVIP	2	2	18	NEUTRAL
ENSG00000198242	RPL23A	1	2	19	NEUTRAL
ENSG00000198363	ASPH	8	0	14	NEUTRAL
ENSG00000198382	UVRAG	2	1	19	NEUTRAL
ENSG00000198799	LRIG2	0	2	20	NEUTRAL
ENSG00000198832	SELM	0	6	16	NEUTRAL
ENSG00000203667	COX20	14	0	8	AMP
ENSG00000203815	FAM231D	15	1	6	AMP
ENSG00000204174	NPY4R	0	1	21	NEUTRAL
ENSG00000204291	COL15A1	0	1	21	NEUTRAL
ENSG00000204371	EHMT2	0	0	22	NEUTRAL
ENSG00000204514	ZNF814	0	0	22	NEUTRAL
ENSG00000204642	HLA-F	0	0	22	NEUTRAL
ENSG00000205213	LGR4	2	2	18	NEUTRAL
ENSG00000205476	CCDC85C	0	1	21	NEUTRAL
ENSG00000205669	ACOT6	0	1	21	NEUTRAL
ENSG00000205838	TTC23L	2	0	20	NEUTRAL
ENSG00000205937	RNPS1	12	0	10	AMP
ENSG00000206344	HCG27	0	0	22	NEUTRAL
ENSG00000206560	ANKRD28	0	0	22	NEUTRAL
ENSG00000213088	DARC	14	0	8	AMP
ENSG00000213213	CCDC183	0	0	22	NEUTRAL
ENSG00000213759	UGT2B11	0	0	22	NEUTRAL
ENSG00000213892	CEACAM16	0	1	21	NEUTRAL
ENSG00000213967	ZNF726	0	1	21	NEUTRAL
ENSG00000213983	AP1G2	1	0	21	NEUTRAL
ENSG00000213988	ZNF90	0	1	21	NEUTRAL
ENSG00000214114	MYCBP	0	3	19	NEUTRAL
ENSG00000214300	SPDYE3	4	0	18	NEUTRAL
ENSG00000214309	MBLAC1	4	0	18	NEUTRAL
ENSG00000214491	SEC14L6	0	6	16	NEUTRAL
ENSG00000214814	FER1L6	9	0	13	NEUTRAL
ENSG00000215021	PHB2	0	1	21	NEUTRAL
ENSG00000215251	FASTKD5	4	0	18	NEUTRAL
ENSG00000215790	SLC35E2	1	2	19	NEUTRAL

Ensembl	Gene	Nb patients AMP	Nb patients DEL	Nb patients NEUTRAL	Majority
ENSG00000216921	AC131097.4	0	1	21	NEUTRAL
ENSG00000218336	TENM3	0	1	21	NEUTRAL
ENSG00000222014	RAB6C	1	0	21	NEUTRAL
ENSG0000223501	VPS52	0	0	22	NEUTRAL
ENSG00000229809	ZNF688	12	0	10	AMP
ENSG00000232859	LYRM9	1	2	19	NEUTRAL
ENSG00000233932	CTXN2	0	3	19	NEUTRAL
ENSG00000239264	TXNDC5	0	1	21	NEUTRAL
ENSG00000241837	ATP5O	2	0	20	NEUTRAL
ENSG00000242259	C22orf39	0	6	16	NEUTRAL
ENSG00000243335	KCTD7	3	0	19	NEUTRAL
ENSG00000243414	TICAM2	0	2	20	NEUTRAL
ENSG00000245848	CEBPA	0	0	22	NEUTRAL
ENSG00000251201	TMED7-TICAM2	0	2	20	NEUTRAL
ENSG00000254004	ZNF260	0	1	21	NEUTRAL
ENSG00000254349	RP11-758M4.1	8	0	14	NEUTRAL
ENSG00000254598	CSNK2A3	2	2	18	NEUTRAL
ENSG00000256229	ZNF486	0	1	21	NEUTRAL
ENSG00000256683	ZNF350	1	0	21	NEUTRAL
ENSG00000257327	RP11-650K20.3	0	0	22	NEUTRAL
ENSG00000257727	CNPY2	0	0	22	NEUTRAL
ENSG00000258315	C17orf49	0	5	17	NEUTRAL
ENSG00000259003	AE000662.92	0	0	22	NEUTRAL
ENSG00000267795	SMIM22	13	0	9	AMP
ENSG00000267918	AL117190.2	0	1	21	NEUTRAL
ENSG00000267970	AC004899.1	4	1	17	NEUTRAL
ENSG00000268080	AC016885.1	9	0	13	NEUTRAL
ENSG00000268923	AL645922.1	0	0	22	NEUTRAL
ENSG00000269047	AC009041.2	12	0	10	AMP
ENSG00000269526	ERVV-1	2	0	20	NEUTRAL
ENSG00000269709	AL109659.1	0	2	20	NEUTRAL
ENSG00000272325	NUDT3	0	0	22	NEUTRAL

Supplemental File 1 /Table S10 : Statistical analysis were conducted to explore the relationship of the PC and ncNAT genes present in the three lists DiffCor, ncNATDiffExp, and VarRatio, and known pronostic factors

For continuous clinicopathological features (age, tumor size, Ki67), a correlation test has been performed for each antisense gene of the 3 lists by computing the Spearman correlation coefficient between the normalized read counts vector and the feature vector. The ratio of antisense genes with an absolute Spearman correlation coefficient greater than 0.6 and a p-value lower than 0.05 has been compared with the same ratio computed with a list of randomly selected antisense genes. For discrete clinicopathological features (T, N, luminal A/B status), a Mann-Whitney test has been performed to compare the read counts distributions between groups of patients. The ratio of antisense genes whose read counts distribution is significantly different between the samples class has been compared with the same ratio computed with a list of randomly selected antisense genes.

		DiffCor	DiffExp	VaRatio – Left	VaRatio – Right	Random
**Continuous factors**	**tumor size**	0,00%	0,00%	0,33%	0,00%	0,08 ± 0,13 %
	**age**	1,54%	0,72%	1,47%	1,50%	0,60 ± 0,36 %
	**Ki67**	0,22%	0,72%	0,00%	1,05%	0,77 ± 0,37 %
**Discrete Factors**	**T**	2,41%	2,16%	3,75%	1,65%	1,58 ± 0,54 %
	**N**	3,07%	4,04%	3,26%	3,01%	2,02 ± 0,58 %
	**Luminal A/B**	1,75%	3,03%	3,75%	3,16%	2,31 ± 0,69 %

	**Supplemental File 1/ Table S11** : Coding genes of the lists DiffCorr, ncNATDiffExp and varRatio (S6-S9) were compared to the Cancer Genes Census from the Cosmic database to extract potential ASO targets				
**Gene Symbol**	**Ensembl PC**	**Somatic**	**Germline**	**Tumour Types (Somatic)**	**Tumour Types (Germline)**
ACSL6	ENSG00000164398	yes		AML, AEL	
AFF1	ENSG00000172493	yes		AL	
AFF3	ENSG00000144218	yes		ALL, T-ALL	
BRCA1	ENSG00000012048	yes	yes	ovarian	breast, ovarian
CAMTA1	ENSG00000171735	yes		epithelioid haemangioendothelioma	
CBFA2T3	ENSG00000129993	yes		AML	
CCND2	ENSG00000118971	yes		NHL,CLL	
CDKN2A	ENSG00000147889	yes	yes	melanoma, multiple other tumour types	melanoma, pancreatic
CEBPA	ENSG00000245848	yes		AML, MDS	
CHD4	ENSG00000111642	yes		uterine serous carcinoma	
CIITA	ENSG00000179583	yes		PMBL, Hodgkin lymphoma	
CRTC3	ENSG00000140577	yes		salivary gland mucoepidermoid	
CTCF	ENSG00000102974	yes		endometrial, breast, head and neck cancer	
DDX6	ENSG00000110367	yes		B-NHL	
EGFR	ENSG00000146648	yes	yes	glioma, NSCLC	NSCLC
ELN	ENSG00000049540	yes		B-ALL	
ETV4	ENSG00000175832	yes		Ewing sarcoma, prostate carcinoma	
FLI1	ENSG00000151702	yes		Ewing sarcoma	
FOXO1	ENSG00000150907	yes		alveolar rhabdomyosarcoma	
GNA11	ENSG00000088256	yes		uveal melanoma, primary central nervous system melanocytic neoplasms	
GNAS	ENSG00000087460	yes		pituitary adenoma, pancreatic intraductal papillary mucinous neoplasm, fibrous dysplasia	
GPC3	ENSG00000147257		yes		Wilms tumour
H3F3A	ENSG00000163041	yes		glioma	

	Supplemental File 1/ Table S11 : Coding genes of the lists DiffCorr, ncNATDiffExp and varRatio (S6-S9) were compared to the Cancer Genes Census from the Cosmic database to extract potential ASO targets				
**Gene Symbol**	**Cancer Syndrome**	**Molecular Genetics**	**Role in Cancer**	**Mutation Types**	**Translo-cation Partner**
ACSL6		Dom		T	ETV6
AFF1		Dom	oncogene	T	KMT2A
AFF3		Dom	oncogene	T	KMT2A, RUNX1
BRCA1	hereditary breast/ovarian cancer	Rec	TSG	D, Mis, N, F, S	
CAMTA1		Dom	TSG	T	WWTR1
CBFA2T3		Dom		T	RUNX1, GLIS2
CCND2		Dom	oncogene	T	IGL
CDKN2A	familial malignant melanoma	Rec	TSG	D, Mis, N, F, S	
CEBPA		Dom	TSG	Mis, N, F	
CHD4		Dom	oncogene	Mis, F, N	
CIITA		Dom	TSG	T	C15orf55, CD274, PDCD1LG2, RALGDS, RUNDC2A, RMI2, BCL6
CRTC3		Dom		T	MAML2
CTCF		Dom	TSG	Mis, N	
DDX6		Dom		T	IGH
EGFR	familial lung cancer	Dom		A, O, Mis	
ELN		Dom		T	PAX5
ETV4		Dom		T	EWSR1, TMPRSS2, DDX5, KLK2, CANT1
FLI1		Dom		T	EWSR1
FOXO1		Dom	oncogene/TSG	T	PAX3, PAX7
GNA11		Dom		Mis	
GNAS		Dom		Mis	
GPC3	Simpson-Golabi-Behmel syndrome	Rec/X		T, D, Mis, N, F, S	
H3F3A		Dom		Mis	

	**Supplemental File 1/ Table S11** : Coding genes of the lists DiffCorr, ncNATDiffExp and varRatio (S6-S9) were compared to the Cancer Genes Census from the Cosmic database to extract potential ASO targets		
**Gene Symbol**	**Other Germline Mut**	**Other Syndrome**	**Synonyms**
ACSL6			FACL6,23305,ACSL6,ENSG00000164398,KIAA0837,LACS5,A
AFF1			MLLT2,AFF1,4299,ENSG00000172493,P51825,AF-4,PBM1
AFF3			MLLT2-like,3899,AFF3,ENSG00000144218,LAF4,P51826
BRCA1			BRCAI,BRCA1,672,ENSG00000012048,IRIS,BRCC1,RNF53,PSCP
CAMTA1			ENSG00000171735,23261,CAMTA1,Q9Y6Y1,KIAA0833
CBFA2T3			ETO2,CBFA2T3,863,ENSG00000129993,ZMYND4,MTGR2,M
CCND2			KIAK0002,894,CCND2,ENSG00000118971,P30279,MGC1027
CDKN2A			CDK4I,CDKN2A,1029,ENSG00000147889,P42771,p14,CMM2,ARF,TP16,p16,MTS1,p19,p16INK4a,INK4a,CDKN2,p16INK4,p
CEBPA			CEBP,1050,CEBPA,C/EBP-alpha,P49715
CHD4			Mi2-BETA,CHD4,1108,ENSG00000111642,Mi-2b,DKFZp686E06161,Q14839
CIITA			P33076,4261,CIITA,ENSG00000179583,MHC2TA,C2TA
CRTC3			FLJ21868,64784,CRTC3,ENSG00000140577,TORC3
CTCF		Mental retardation, autosomal dominant 21	P49711,10664,CTCF,ENSG00000102974
DDX6			P54,DDX6,1656,ENSG00000110367,HLR2,P26196,RCK,FLJ3
EGFR			mENA,EGFR,1956,ENSG00000146648,P00533,ERBB1,ERBB
ELN	yes	Supravalvular Aortic Stenosis, Cutis laxa , Williams-Beuren Syndrome	ENSG00000049540,2006,ELN,WS,WBS,SVAS,FLJ43523,FLJ38671
ETV4			PEA3,ETV4,2118,ENSG00000175832,E1AF,E1A-F,PEAS3
FLI1			SIC-1,2313,FLI1,ENSG00000151702,Q01543,EWSR2
FOXO1			Q12778,FOXO1A,2308,FOXO1,ENSG00000150907,FKH1,FKHR,FOXO1
GNA11			GNA-11,GNA11,2767,ENSG00000088256
GNAS	yes	McCune-Albright syndrome; pseudohypoparathyroidism, type IA	RP4-543J19_4,2778,GNAS,ENSG00000087460,NESP55,NESP,GSP,POH,GNASXL,GNAS1,C20orf45,dJ309F20_1_1,SCG6,GPSA,Gnas,dJ806M20_3_3,XL2,MGC33735,XL,PHP1A,GSA,AHO,XLalphas,PHP1B
GPC3			OCI-5,GPC3,2719,ENSG00000147257,SGBS,SGB,DGSX,SDYS,P51654,AC002420_1,SGBS1
H3F3A			H3F3A,P84243,H3F3A,H3_3B,MGC87783,MGC87782,H3F3,H3_3A,ENSG00000163041,3020

Gene Symbol	Ensembl PC	Somatic	Germline	Tumour Types (Somatic)	Tumour Types (Germline)
HIF1A	ENSG00000100644	yes		endometrioid carcinoma, glioblastoma, colorectal, renal, lung, pancreatic	
HLF	ENSG00000108924	yes		ALL	
HNF1A	ENSG00000135100	yes	yes	hepatic adenoma, hepatocellular	hepatic adenoma, hepatocellular carcinoma
HOXC11	ENSG00000123388	yes		AML	
HOXC13	ENSG00000123364	yes		AML	
IL6ST	ENSG00000134352	yes		hepatocellular carcinoma	
KDR	ENSG00000128052	yes		NSCLC, angiosarcoma	
KDSR	ENSG00000119537	yes		B-NHL	
KMT2C	ENSG00000055609	yes		medulloblastoma	
SPEN	ENSG00000065526	yes		SMZL, adenoid cystic carcinoma, DLBCL	
KTN1	ENSG00000126777	yes		papillary thyroid	
LEF1	ENSG00000138795	yes		B-ALL, T-ALL, eyelid sebaceous carcinoma, AML, lymphomas	
LIFR	ENSG00000113594	yes		salivary adenoma	
LPP	ENSG00000145012	yes		lipoma, leukaemia	
LZTR1	ENSG00000099949	yes	yes	glioblastoma	glioblastoma, schwannoma
MAP2K1	ENSG00000169032	yes		NSCLC, melanoma, colorectal	
MAP3K13	ENSG00000073803	yes		breast	
MDM2	ENSG00000135679	yes		sarcoma, glioma, colorectal, other tumour types	
MKL1	ENSG00000196588	yes		acute megakaryocytic leukaemia	
MSH2	ENSG00000095002	yes	yes	colorectal, endometrial, ovarian	colorectal, endometrial, ovarian
MSH6	ENSG00000116062	yes	yes	colorectal	colorectal, endometrial, ovarian
MTCP1	ENSG00000214827	yes		T cell prolymphocytic leukaemia	
MYH11	ENSG00000133392	yes		AML	
NCKIPSD	ENSG00000213672	yes		ALL	
NFIB	ENSG00000147862	yes		adenoid cystic carcinoma, lipoma	
PDE4DIP	ENSG00000178104	yes		MPN	
PIK3CA	ENSG00000121879	yes		colorectal, gastric, glioblastoma, breast	
POT1	ENSG00000128513	yes		CLL	
PPFIBP1	ENSG00000110841	yes		Spitzoid tumour, inflammatory myofibroblastic tumour	
PPP6C	ENSG00000119414	yes		melanoma	
PTPRB	ENSG00000127329	yes		angiosarcoma	

Gene Symbol	Cancer Syndrome	Molecular Genetics	Role in Cancer	Mutation Types	Translo-cation Partner
HIF1A		Dom	oncogene	Mis, F, N	
HLF		Dom	oncogene	T	TCF3
HNF1A	familial hepatic adenoma	Rec		Mis, F	
HOXC11		Dom	oncogene	T	NUP98
HOXC13		Dom	oncogene	T	NUP98
IL6ST		Dom		O	
KDR		Dom		Mis	
KDSR		Dom		T	IGK
KMT2C		Rec		N	
SPEN		Rec		F, Mis, N	
KTN1		Dom		T	RET
LEF1			oncogene/TSG	Mis, N	
LIFR		Dom		T	PLAG1
LPP		Dom		T	HMGA2, KMT2A, C12orf9
LZTR1	Schwannomatosis-2	Dom	TSG	Mis, F, N	
MAP2K1		Dom	oncogene	Mis	
MAP3K13		Rec		F	
MDM2		Dom	oncogene	A	
MKL1		Dom		T	RBM15
MSH2	hereditary non-polyposis colorectal cancer	Rec		D, Mis, N, F, S	
MSH6	hereditary non-polyposis colorectal cancer	Rec		Mis, N, F, S	
MTCP1		Dom		T	TRA
MYH11		Dom		T	CBFB
NCKIPSD		Dom		T	KMT2A
NFIB		Dom		T	MYB, HGMA2
PDE4DIP		Dom		T	PDGFRB
PIK3CA		Dom	oncogene	Mis	
POT1		Dom		Mis, N	
PPFIBP1		Dom		T	ROS1, ALK
PPP6C		Dom		Mis, N	
PTPRB		Rec		N, Mis, S, F	

Gene Symbol	Other Germline Mut	Other Syndrome	Synonyms
HIF1A			Q16665,3091,HIF1A,ENSG00000100644,MOP1,HIF1-ALPHA,PASD8,HIF-1alpha
HLF			Q16534,3131,HLF,ENSG00000108924
HNF1A	yes	Maturity-onset diabetes of the young, TYPE III	HNF1,6927,HNF1A,ENSG00000135100,TCF1,MODY3,HNF1A,P20823,LFB1
HOXC11			HOX3H,3227,HOXC11,ENSG00000123388,O43248
HOXC13			HOX3G,3229,HOXC13,ENSG00000123364,P31276,HOX3
IL6ST			CDw130,3572,IL6ST,ENSG00000134352,P40189,IL6R-beta,GP130-RAPS,GP130,CD130
KDR			VEGFR2,3791,KDR,ENSG00000128052,P35968,VEGFR,CD3
KDSR			Q06136,KDSR,2531,ENSG00000119537,FVT1
KMT2C			ENSG00000055609,KMT2C,MLL3,58508
SPEN			Q96T58,SPEN,23013,ENSG00000065526,SHARP,RP1-134O19_1,MINT,KIAA0929
KTN1			Q86UP2,KTN1,3895,ENSG00000126777,KIAA0004,CG1
LEF1			TCF1ALPHA,51176,LEF1,ENSG00000138795,Q9UJU2,DKFZp586H0919
LIFR			SJS2,LIFR,3977,ENSG00000113594,SWS,STWS,CD118
LPP			Q93052,4026,LPP,ENSG00000145012
LZTR1	yes	Noonan syndrome 10	ENSG00000099949,8216,LZTR1,Q8N653,TCFL2,MGC21205,LZTR-1
MAP2K1	yes	Cardiofaciocutaneous syndrome	MAPKK1,5604,MAP2K1,ENSG00000169032,Q02750,MEK1,MKK1,PRKMK1
MAP3K13			MGC133196,9175,MAP3K13,ENSG00000073803,LZK
MDM2			HDM2,4193,MDM2,ENSG00000135679,HDMX,MGC71221,hdm2,Q00987
MKL1			BSAC,57591,MKL1,ENSG00000196588,Q969V6,MRTF-A
MSH2			COCA1,4436,MSH2,ENSG00000095002,HNPCC,FCC1,HNPCC1,P43246
MSH6			GTBP,MSH6,P52701,2956,ENSG00000116062,HSAP,HNPCC5
MTCP1			C6_1B,MTCP1,4515,ENSG00000214827,GS1-273L24_4
MYH11			ENSG00000133392,MYH11,4629
NCKIPSD			AF3P21,NCKIPSD,51517,ENSG00000213672,SPIN90,WASLBP,Q9NZQ3,WISH,ORF1
NFIB			RP11-280O24_2,4781,NFIB,ENSG00000147862,NFIB2,NFI-RED,NFIB3
PDE4DIP			ENSG00000178104,PDE4DIP,9659
PIK3CA			MGC142161,5290,PIK3CA,ENSG00000121879,P42336,MGC142163,p110-alpha,PI3K
POT1			DKFZP586D211,POT1,25913,ENSG00000128513,hPot1,DKFZ
PPFIBP1			L2,8496,PPFIBP1,ENSG00000110841,hSGT2,hSgt2p
PPP6C			O00743,PPP6C,5537,ENSG00000119414
PTPRB			ENSG00000127329,PTPRB,5787,MGC142023,DKFZp686H15164,MGC59935,PTPB,HPTP-BETA,P23467,HPTPB,R-PTP-

Gene Symbol	Ensembl PC	Somatic	Germline	Tumour Types (Somatic)	Tumour Types (Germline)
PTPRT	ENSG00000196090	yes		HNSCC, colorectal cancer, gastric cancer, lung cancer, melanoma	
RAP1GDS1	ENSG00000138698	yes		T-ALL	
RB1	ENSG00000139687	yes	yes	retinoblastoma, sarcoma, breast, small cell lung carcinoma	retinoblastoma, sarcoma, breast, small cell lung carcinoma
RECQL4	ENSG00000160957		yes		osteosarcoma, skin basal cell, skin sqamous cell
RSPO2	ENSG00000147655	yes		colorectal	
RUNX1	ENSG00000159216	yes		AML, pre B-ALL, T-ALL	
STAG2	ENSG00000101972	yes		bladder carcinoma, glioblastoma, melanoma, Ewing's sarcoma, myeloid neoplasms	
TAL1	ENSG00000162367	yes		lymphoblastic leukaemia/biphasic	
TCEA1	ENSG00000187735	yes		salivary adenoma	
TCL1A	ENSG00000100721	yes		T-CLL	
TFEB	ENSG00000112561	yes		renal cell carcinoma (childhood epithelioid)	
TSHR	ENSG00000165409	yes	yes	toxic thyroid adenoma	thyroid adenoma
UBR5	ENSG00000104517	yes		mantle cell lymphoma, gastric, colorectal	
VTI1A	ENSG00000151532	yes		colorectal	
WT1	ENSG00000184937	yes	yes	Wilms tumour, desmoplastic small round cell tumour	Wilms tumour
XPC	ENSG00000154767		yes		skin basal cell, skin squamous cell, melanoma
ZBTB16	ENSG00000109906	yes		APL	
ZFHX3	ENSG00000140836	yes		endometrial, gastric, prostate	

Gene Symbol	Cancer Syndrome	Molecular Genetics	Role in Cancer	Mutation Types	Translo-cation Partner
PTPRT			TSG	Mis, N	
RAP1GDS1		Dom		T	NUP98
RB1	familial retinoblastoma	Rec	TSG	D, Mis, N, F, S	
RECQL4	Rothmund-Thompson syndrome	Rec		N, F, S	
RSPO2		Dom		T	EIF3E
RUNX1		Dom	oncogene/TSG	T	RPL22, MECOM, CBFA2T3, RUNX1T1, ETV6, AFF3
STAG2		Rec		Mis, N, F, S	
TAL1		Dom		T	TRD, STIL
TCEA1		Dom		T	PLAG1
TCL1A		Dom		T	TRA
TFEB		Dom		T	ALPHA
TSHR		Dom		Mis	
UBR5		Rec		F, N, Mis, S	
VTI1A		Dom		T	TCF7L2
WT1	Denys-Drash syndrome, Frasier syndrome, familial Wilms tumour	Rec		D, Mis, N, F, S, T	EWSR1
XPC	xeroderma pigmentosum (C)	Rec	TSG	Mis, N, F, S	
ZBTB16		Dom		T	RARA
ZFHX3		Dom	TSG	Mis, N	

Gene Symbol	Other Germline Mut	Other Syndrome	Synonyms
PTPRT			KIAA0283,11122,PTPRT,ENSG00000196090,O14522,RPTPrho,RP5-1121H13_2
RAP1GDS1			P52306,5910,RAP1GDS1,MGC118861,MGC118859,GDS1,ENSG00000138698
RB1			OSRC,ENSG00000139687,RB1,5925,RB,P06400
RECQL4			RecQ4,9401,RECQL4,ENSG00000160957,RECQ4,RTS
RSPO2			Q6UXX9,RSPO2,340419,ENSG00000147655,MGC43342,MG
RUNX1			PEBP2A2,RUNX1,861,ENSG00000159216,Q01196,AML1,CBFA2,AMLCR1
STAG2			RP11-517O1_1,10735,STAG2,ENSG00000101972,DKFZp781H1753,SA2,SA-2,FLJ25871,DKFZp686P168,bA517O1_1
TAL1			TCL5,TAL1,6886,ENSG00000162367,SCL,P17542,tal-1
TCEA1			ENSG00000187735,6917,TCEA1
TCL1A			P56279,TCL1A,8115,ENSG00000100721,TCL1
TFEB			TCFEB,TFEB,7942,ENSG00000112561,P19484,AlphaTFEB
TSHR	yes	Hereditary nonautoimmune hyperthyroidism; subclinical hypothyroidism	hTSHR-I,7253,TSHR,ENSG00000165409,MGC75129,LGR3
UBR5			EDD,UBR5,51366,ENSG00000104517,EDD1,KIAA0896,HYD,MGC57263,DD5,O95071,FLJ11310
VTI1A			ENSG00000151532,Vti1-rp2,MVti1,143187,VTI1A
WT1			WAGR,7490,WT1,ENSG00000184937,WT33,GUD,WIT-2
XPC			XP3,7508,XPC,ENSG00000154767,XPCC
ZBTB16			Q05516,7704,ZBTB16,ENSG00000109906,ZNF145,PLZF
ZFHX3			ATBT,463,ZFHX3,ENSG00000140836,Q15911,ZFHX3

	**Supplemental File 1/ Table S12. Picard metrics** Picard quality control analyses. BCTP annotation corresponds to tumors and BCTN to adjacent non-malignant tissues, the following numbers are the patient's ID.				
**Sample ID**	**PF_BASES**	**PF_ALIGNED _BASES**	**INCORRECT____ STRAND_READS**	**PCT_RIBOSOMAL_ BASES**	**PCT_CODING _BASES**
**BCTN12-02**	7,79E+009	6,62E+009	1,02E+006	7,48%	6,56%
**BCTP12-02**	7,06E+009	5,85E+009	9,05E+005	0,65%	1,14%
**BCTN12-03**	7,90E+009	6,63E+009	1,22E+006	4,25%	3,54%
**BCTP12-03**	7,21E+009	6,28E+009	7,34E+005	4,11%	5,78%
**BCTN13-02**	5,75E+009	5,45E+009	6,01E+005	18,23%	12,80%
**BCTP13-02**	4,97E+009	4,76E+009	5,49E+005	21,11%	14,63%
**BCTN13-03**	7,24E+009	7,00E+009	8,99E+005	22,06%	20,14%
**BCTP13-03**	2,81E+009	2,68E+009	3,22E+005	17,07%	15,86%
**BCTN13-05**	7,02E+009	6,77E+009	8,37E+005	14,28%	10,93%
**BCTP13-05**	6,86E+009	6,60E+009	9,71E+005	18,14%	18,12%
**BCTN13-07**	3,98E+009	3,76E+009	4,86E+005	19,92%	17,16%
**BCTP13-07**	1,88E+010	1,81E+010	2,47E+006	13,25%	14,13%
**BCTN13-10**	1,12E+010	1,07E+010	1,37E+006	12,87%	22,45%
**BCTP13-10**	9,38E+009	9,02E+009	1,01E+006	6,15%	11,91%
**BCTN13-11**	3,69E+009	3,44E+009	4,08E+005	10,62%	12,94%
**BCTP13-11**	1,28E+010	1,20E+010	1,13E+006	5,64%	10,71%
**BCTN13-13**	6,89E+009	5,98E+009	8,64E+005	9,00%	6,23%
**BCTP13-13**	7,87E+009	6,68E+009	1,18E+006	6,20%	6,85%
**BCTN13-17**	5,05E+009	4,40E+009	6,77E+005	10,43%	7,41%
**BCTP13-17**	8,25E+009	7,43E+009	7,96E+005	12,97%	10,58%
**BCTN14-25**	8,02E+009	7,21E+009	9,95E+005	7,25%	7,90%
**BCTP14-25**	8,32E+009	7,78E+009	9,65E+005	10,00%	12,32%
**BCTN14-31**	8,44E+009	7,44E+009	1,04E+006	6,66%	7,57%
**BCTP14-31**	8,65E+009	7,26E+009	1,33E+006	2,04%	4,07%
**BCTN14-33**	7,45E+009	6,40E+009	8,02E+005	2,72%	3,68%
**BCTP14-33**	1,07E+010	1,01E+010	8,88E+005	3,58%	11,69%
**BCTN14-34**	8,82E+009	7,47E+009	1,23E+006	5,89%	5,61%
**BCTP14-34**	6,82E+009	6,19E+009	6,28E+005	5,55%	7,71%
**BCTN14-35**	7,71E+009	7,17E+009	1,19E+006	7,06%	7,59%
**BCTP14-35**	9,08E+009	8,53E+009	1,15E+006	9,19%	12,27%
**BCTN14-36**	8,72E+009	8,38E+009	9,37E+005	12,30%	15,95%
**BCTP14-36**	6,83E+009	6,52E+009	1,13E+006	3,13%	9,58%
**BCTN14-38**	9,57E+009	9,26E+009	1,17E+006	11,68%	20,67%
**BCTP14-38**	8,57E+009	8,11E+009	8,09E+005	11,13%	12,91%
**BCTN14-41**	8,73E+009	8,24E+009	1,02E+006	18,02%	20,43%
**BCTP14-41**	9,27E+009	8,56E+009	1,20E+006	12,08%	18,78%
**BCTN15-01**	8,71E+009	8,43E+009	7,52E+005	15,73%	13,35%
**BCTP15-01**	1,03E+010	1,01E+010	8,73E+005	20,79%	12,94%
**BCTN15-02**	9,05E+009	8,63E+009	8,67E+005	25,72%	15,30%
**BCTP15-02**	9,42E+009	9,06E+009	9,80E+005	11,06%	18,12%
**BCTN15-03**	1,15E+010	1,10E+010	1,11E+006	15,19%	13,65%
**BCTP15-03**	9,69E+009	9,35E+009	8,08E+005	11,08%	11,79%
**BCTN15-04**	1,27E+010	1,16E+010	1,38E+006	10,43%	10,86%
**BCTP15-04**	1,15E+010	1,08E+010	1,85E+006	2,02%	5,34%

	**Supplemental File 1/ Table S12. Picard metrics** Picard quality control analyses. BCTP annotation corresponds to tumors and BCTN to adjacent non-malignant tissues, the following numbers are the patient's ID.			
**Sample ID**	**PCT_UTR_BASES**	**PCT_coding + UTR_bases**	**PCT_INTRONIC_BASES**	**PCT_INTERGENIC_BASES**
**BCTN12-02**	15,46%	22,02%	41,16%	29,61%
**BCTP12-02**	12,81%	13,95%	46,45%	38,98%
**BCTN12-03**	13,50%	17,04%	44,37%	34,44%
**BCTP12-03**	16,11%	21,89%	47,86%	26,22%
**BCTN13-02**	33,18%	45,98%	28,80%	7,44%
**BCTP13-02**	24,99%	39,62%	32,10%	7,53%
**BCTN13-03**	31,03%	51,18%	22,91%	4,19%
**BCTP13-03**	21,84%	37,70%	38,79%	6,81%
**BCTN13-05**	16,03%	26,96%	41,70%	17,31%
**BCTP13-05**	22,63%	40,76%	32,81%	8,52%
**BCTN13-07**	24,21%	41,37%	29,65%	9,35%
**BCTP13-07**	17,96%	32,09%	39,39%	15,49%
**BCTN13-10**	37,88%	60,33%	23,21%	3,91%
**BCTP13-10**	30,91%	42,83%	40,02%	11,16%
**BCTN13-11**	37,06%	50,00%	29,61%	10,06%
**BCTP13-11**	53,87%	64,58%	23,71%	6,35%
**BCTN13-13**	14,74%	20,97%	43,00%	27,23%
**BCTP13-13**	15,57%	22,42%	48,02%	23,59%
**BCTN13-17**	17,31%	24,72%	39,47%	25,61%
**BCTP13-17**	19,74%	30,32%	38,61%	18,33%
**BCTN14-25**	17,62%	25,52%	47,97%	19,45%
**BCTP14-25**	25,93%	38,25%	42,86%	9,25%
**BCTN14-31**	16,36%	23,93%	45,20%	24,34%
**BCTP14-31**	15,23%	19,30%	44,39%	34,36%
**BCTN14-33**	22,34%	26,02%	42,51%	28,81%
**BCTP14-33**	54,95%	66,64%	24,04%	5,88%
**BCTN14-34**	15,54%	21,15%	41,77%	31,37%
**BCTP14-34**	34,22%	41,93%	35,63%	17,03%
**BCTN14-35**	11,47%	19,05%	41,22%	32,80%
**BCTP14-35**	19,97%	32,23%	40,15%	18,67%
**BCTN14-36**	21,41%	37,37%	42,91%	7,71%
**BCTP14-36**	14,80%	24,38%	40,92%	31,66%
**BCTN14-38**	24,58%	45,25%	35,71%	7,58%
**BCTP14-38**	29,34%	42,24%	39,94%	7,05%
**BCTN14-41**	25,70%	46,13%	28,33%	7,97%
**BCTP14-41**	26,98%	45,76%	31,29%	11,25%
**BCTN15-01**	20,58%	33,93%	45,41%	5,29%
**BCTP15-01**	17,21%	30,15%	44,84%	4,60%
**BCTN15-02**	21,79%	37,08%	30,67%	6,87%
**BCTP15-02**	24,27%	42,39%	41,76%	5,11%
**BCTN15-03**	19,70%	33,35%	44,12%	7,69%
**BCTP15-03**	18,88%	30,67%	51,44%	7,06%
**BCTN15-04**	47,78%	58,64%	21,82%	9,61%
**BCTP15-04**	20,16%	25,50%	39,91%	32,64%

Supplemental File 1/ Figure S13 : Picard metrics Summary. Picard quality control analyses : Average values found in adjacent non-malignant and tumoral tissues. Mean values obtained in non-malignant and tumoral tissues are similar.

PF_BASES	PF_BASE S	PF_ALIGNED_ BASES	INCORRECT___ _STRAND_REA DS	PCT_RIBOSOMAL_ BASES	PCT_CODING_ BASES	PCT_UTR_ BASES	PCT_coding + UTR_bases	PCT_INTRONIC_ BASES	PCT_INTERGENIC _BASES
**Average adj. No-malignant tissue**	7,65E+009	7,04E+009	9,08E+005	11,64%	11,42%	21,97%	33,39%	35,28%	15,59%
**Average tumor**	8,88E+009	8,26E+009	1,03E+006	9,41%	11,24%	24,47%	35,71%	39,31%	15,80%

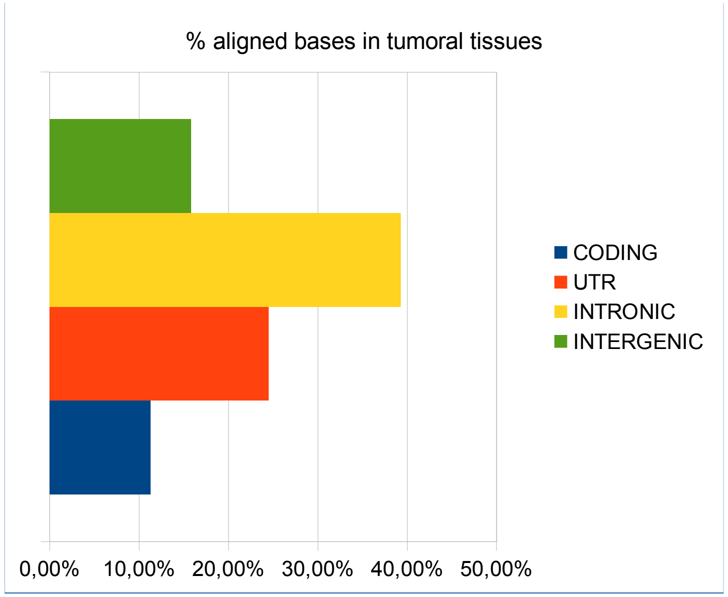

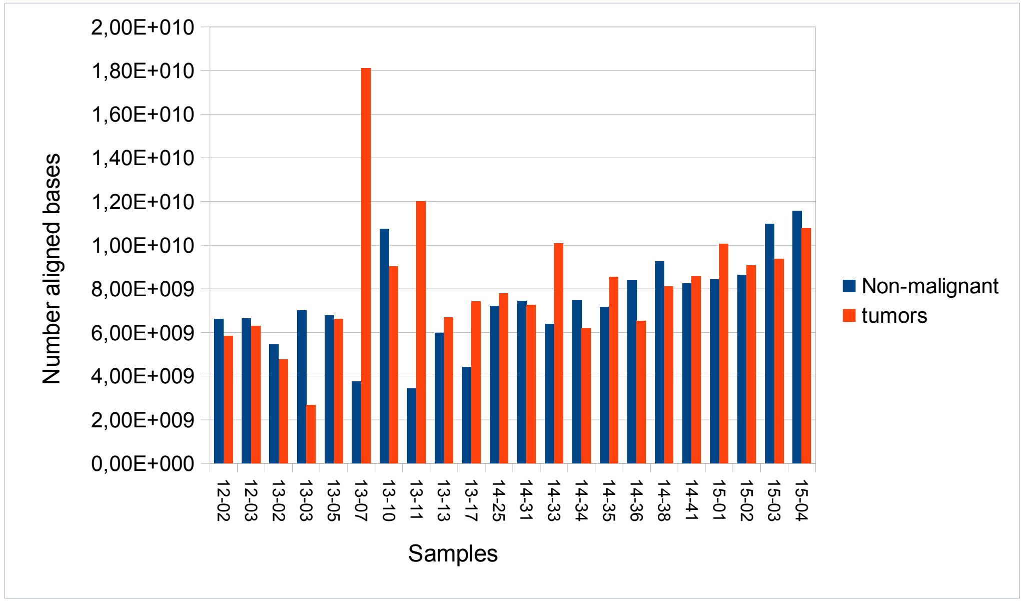

Supplemental File 1/ Figure S14. Data Quality assessment : The Strand Specificity %, calculated as the ratio of sequencing reads mapping to the incorrect strands, was computed for all samples with htseq-count. The patient who presented an outlier value for stand specificity in her tumoral tissue (indicated in red) was excluded for further analysis.

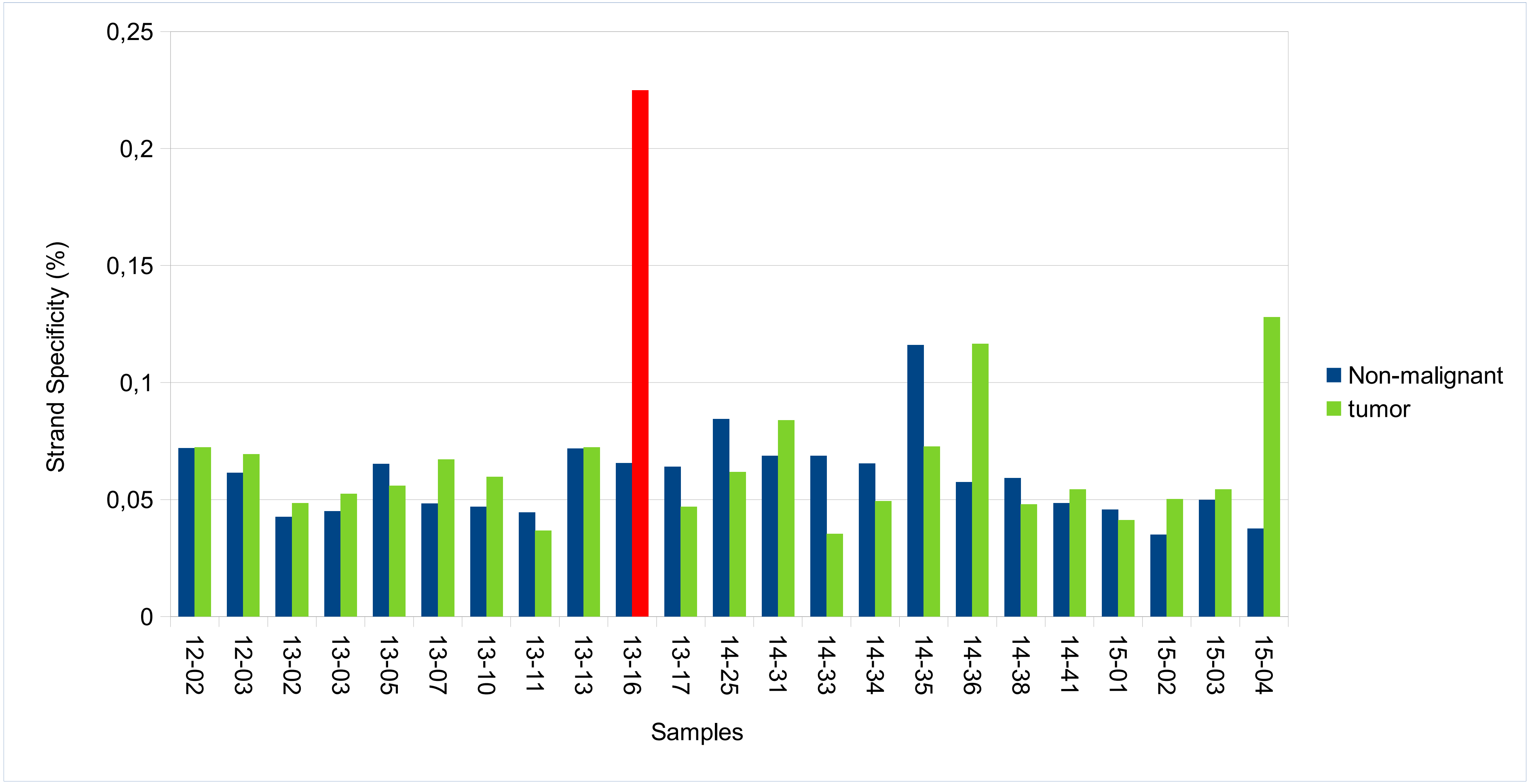

Supplemental File 1/ Figure S15 : Data Quality assessment : MA-plots representing the log2 fold change versus mean expression of the whole dataset values computed with DESq2. Values in red are statistically significant differntially expressed between healthy and tumoral tissues

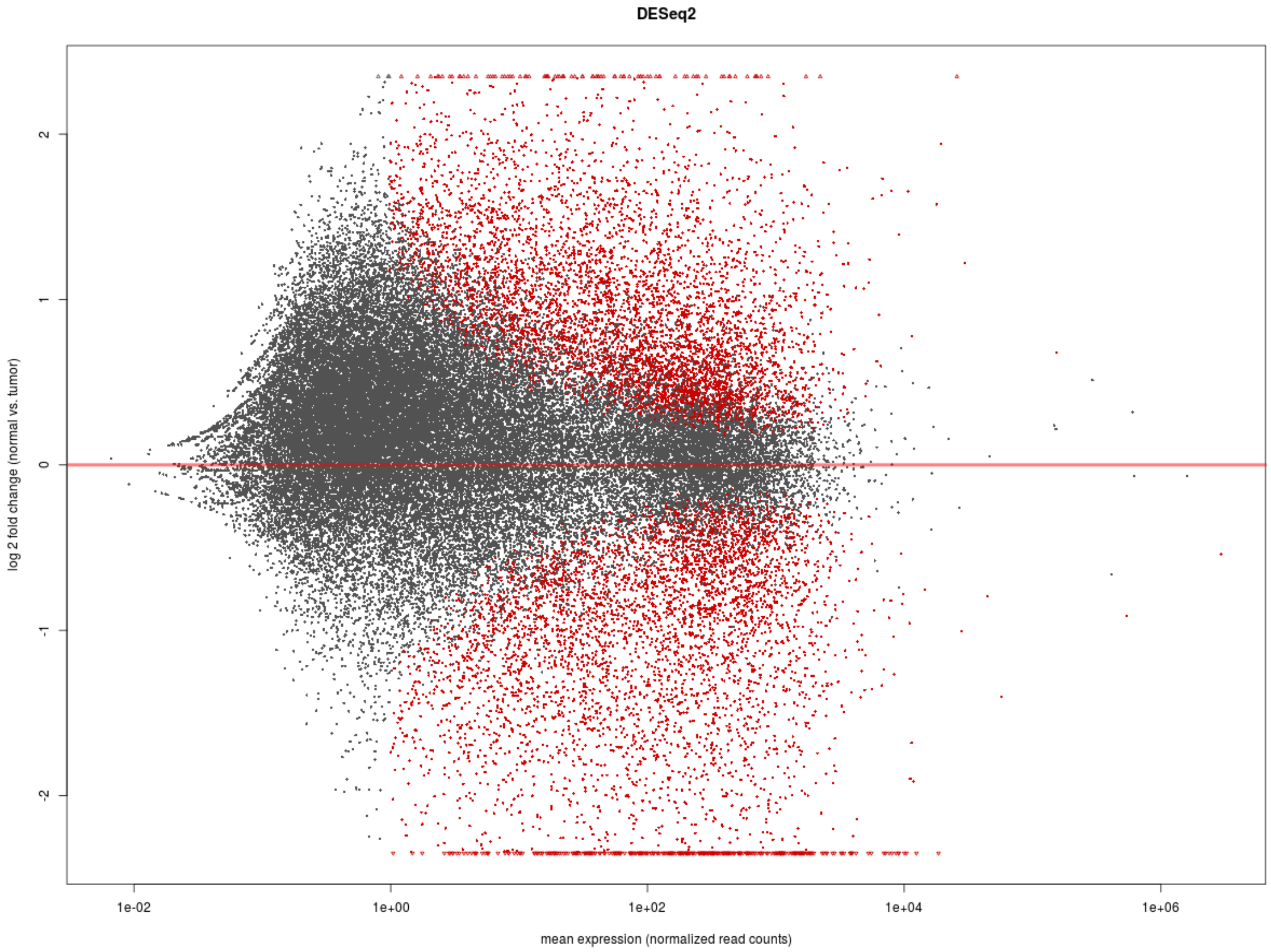

Supplemental File 1/ Figure S16. Data Quality assessment : boxplot of Cook's distances for all samples were computed with DESeq2

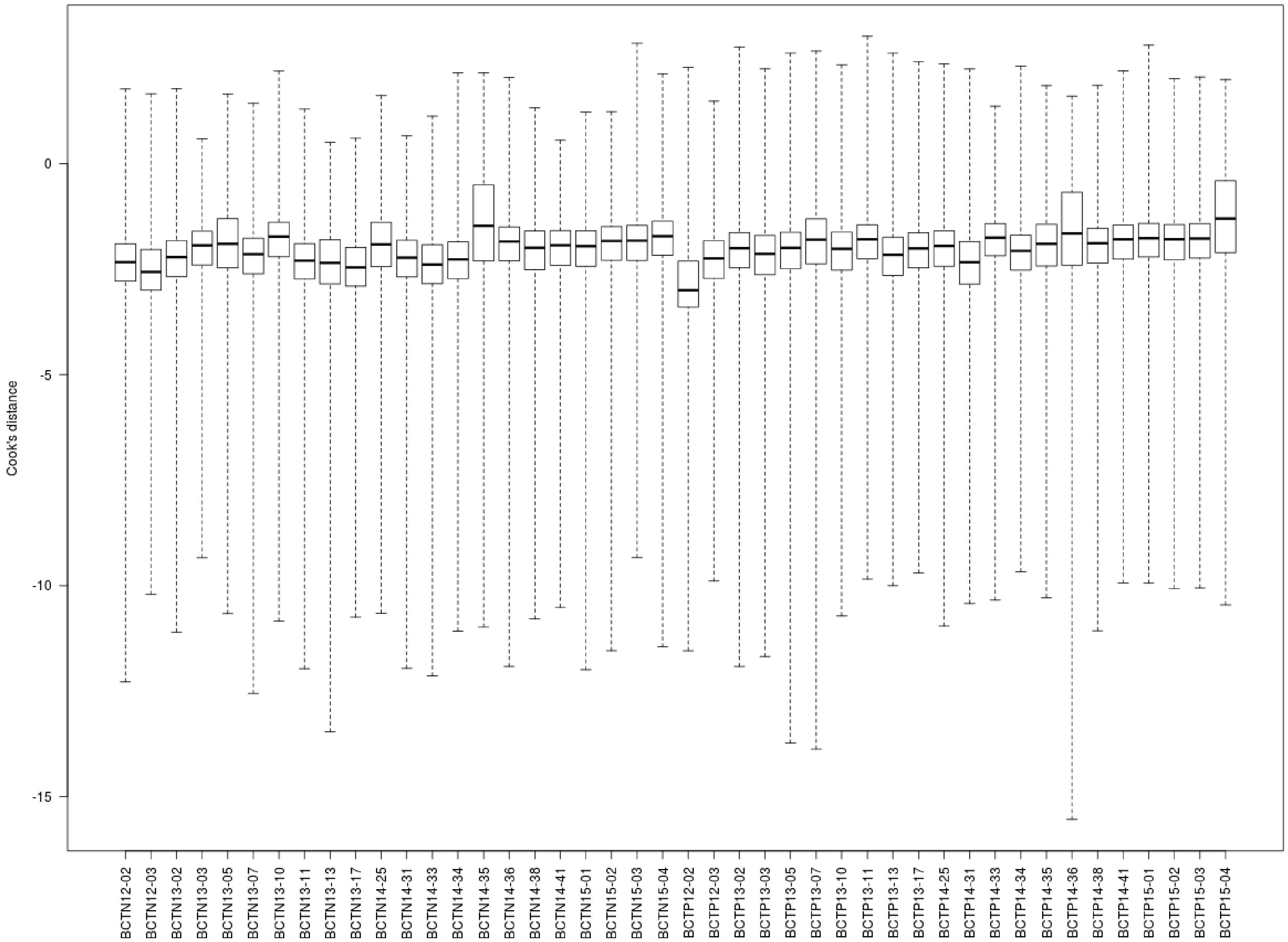

Supplemental File 1/ Figure S17. Independent filtering using the mean of normalized read counts as filter statistic (DESeq2). The optimal threshold is set at 33%. Each dot represents a gene. Genes are ranked along their mean expression value at the y-axis. The x-axis represents the p-value of the differential expression test performed with DESeq2. Genes with a mean expression value lower than the set threshold (at the left of the green vertical line) are removed.

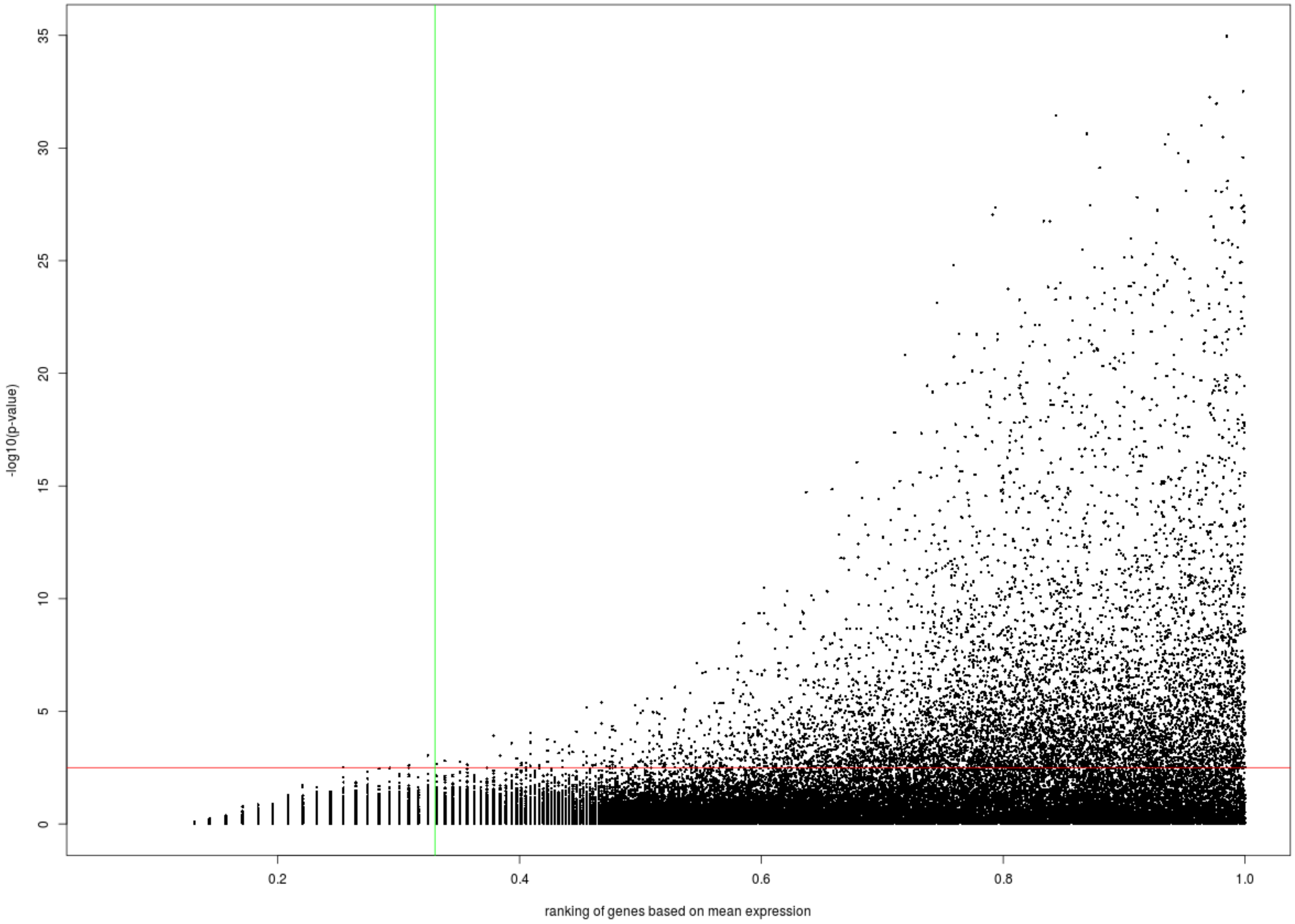

Supplemental File 1/ Figure S18 : Data Quality assessment : a principal component plot analysis was computed with the normalized read counts matrix. Adjacent non-malignant and tumoral tissues are segregated.

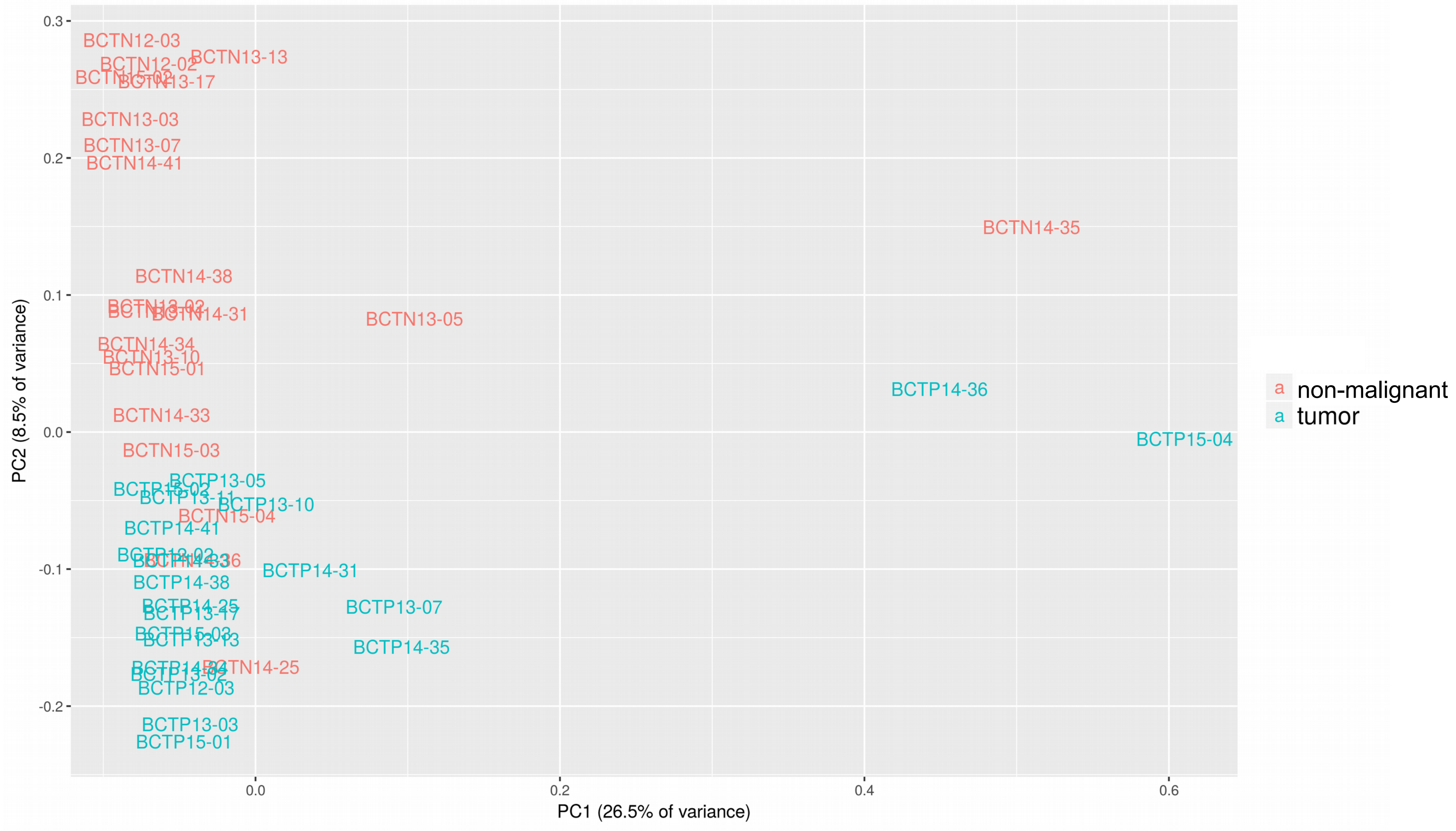

Supplemental File 1 : Additional Material and Methods

DNA/RNA/miRNA extraction

DNA, RNA and miRNA were simultaneously extracted using All Prep DNA/RNA/miRNA Universal kit (Qiagen, Belgium) according to the manufacturer protocol. The RNA quality was assessed using a BioAnalyzer (Agilent, Belgium).

TruSeq® Stranded Total RNA by Illumina® and next generation sequencing

A step of chemical fragmentation generated RNA fragment of 180 pb. This step was adapted according to the RNA quality as described in the manufacturer's protocol. The syntheses of first and second strand of cDNA were performed with hexamer random primers. The 3'-ends of cDNA were adenylated and subjected to adapter ligation. A 12 cycles PCR was performed to amplify the libraries. The quality and the size of cDNA libraries were assessed using Bioanalyzer Agilent Chip DNA 1000. Only libraries from 290bp to 300pb were used, and 14 pmol final cDNA libraries were loaded on a Illumina HiSeq 2000 for cluster generation and paired-end sequencing of 2x100 bp. Four samples were multiplexed on one flow cell.

Array CGH

Array comparative genomic hybridization was performed in the healthy and tumorous tissues of the 22 patients using the Agilent's 60 K microarray platform (G4827A-031746; Agilent Technologies, Santa Clara, CA, USA) according to the manufacturer's instructions. The arrays were scanned with SureScan High Resolution Microarray Scanner (Agilent Technologies, Santa Clara, CA, USA). Data and images were imported using the Feature Extraction V.9.5.3.1 Software and results were analyzed with CytoGenomics software v2.5 (Agilent Technologies, Santa Clara, CA, USA). The Aberration Detection Methods 2 algorithm (ADM-2) was used with a cut-off 6.0, followed by a filter to select regions with three or more adjacent probes and a minimum average log2 ratio ± 0.25, was used to detect copy number changes. The quality of each experiment was assessed by the measurement of the derivative log ratio spread with CytoGenomics software v2.0. Genomic positions were based on the UCSC February 2009 human reference sequence (hg19) (NCBI build 37 reference sequence assembly). Filtering of copy number changes was carried out using the BENCHlab CNV software (Cartagenia, Leuven, Belgium).

Quantitative RT-qPCR

Reverse transcription was performed using the Reverse Aid H Minus kit (LifeTechnologies, Belgium) from 100 ng of total RNA using random hexamer primers in case of coding genes and using a target specific primers coupled to an unrelated synthetic DNA oligonucleotide in case of NAT.

Quantitative PCR were performed using specific 6-FAM/ZEN/IBFQ probes (IDT, Belgium) with Kapa Probe Fast qPCR Master Mix (Sopachem, Belgium) on a LightCycler 480 apparatus (Roche). In case of coding gene amplification, the primers were designed according to standard procedure. In case of NAT gene amplification, a primer specific to the target NAT and a primer specific to the synthetic oligonucleotide added during the reverse transcription are used to increase the stand specificity of the amplification.

The relative expression was calculated using the standard curves methods, using β2-microglobuline as endogenous standard.

ADAMTS9-AS and ADAMTS9-S primers sequences

Transcripts (Ensembl ref)	Exon	Primers	Sequence (5'→3')
ADAMTS9- AS2 (ENST00000485174)	3	ADAMTS9-AS2_RT	CTCCTGGACTATGTCCGGGAACTTTACACCTGTTGCGGGGAA
		ADAMTS9 AS2_qPCR_Probe	GGCCTCACATAAACCCCACCACACCTCTGTCCTGG
		ADAMTS9AS_qPCR_F	GCAGGTAAGAAACCTGAACTGCGT
		ADAMTS9AS_qPCR_R	CTCCTGGACTATGTCCGGGAAC
ADAMTS9-S (ENST00000498707)	2-3	ADAMTS9-S_qPCR_F	AAGCGGAACTCAAGCACTGTTTC
		ADAMTS9-S_qPCR_R	ATCCCCATCATGAGACCGGAATGT
